# Transcription factor co-expression mediates lineage priming for embryonic and extra-embryonic differentiation

**DOI:** 10.1016/j.stemcr.2023.12.002

**Published:** 2024-01-11

**Authors:** Alba Redó-Riveiro, Jasmina Al-Mousawi, Madeleine Linneberg-Agerholm, Martin Proks, Marta Perera, Nazmus Salehin, Joshua M. Brickman

**Affiliations:** 1 reNEW UCPH – The Novo Nordisk Foundation Center for Stem Cell Medicine, Faculty of Health and Medical Sciences, University of Copenhagen, Blegdamsvej 3B, 2200 Copenhagen N, Denmark

**Keywords:** Transcription, Enhancer, Endoderm, Epiblast, Blastocyst, Pluripotency, Cooperativity, Embryonic Stem Cells, nEnd, Lineage Priming

## Abstract

In early mammalian development, cleavage stage blastomeres and inner cell mass (ICM) cells co-express embryonic and extra-embryonic transcriptional determinants. Using a protein-based double reporter we identify an embryonic stem cell (ESC) population that co-expresses the extra-embryonic factor GATA6 alongside the embryonic factor SOX2. Based on single cell transcriptomics, we find this population resembles the unsegregated ICM, exhibiting enhanced differentiation potential for endoderm while maintaining epiblast competence. To relate transcription factor binding in these cells to future fate, we describe a complete enhancer set in both ESCs and naive extra-embryonic endoderm stem cells and assess SOX2 and GATA6 binding at these elements in the ICM-like ESC sub-population. Both factors support cooperative recognition in these lineages, with GATA6 bound alongside SOX2 on a fraction of pluripotency enhancers and SOX2 alongside GATA6 more extensively on endoderm enhancers, suggesting that cooperative binding between these antagonistic factors both supports self-renewal and prepares progenitor cells for later differentiation.

## Highlights

• A compendium of *in vitro* enhancers for naive pluripotency and naive extra-embryonic endoderm
• Unsegregated ICM-like cells arise in KOSR media, which co-express SOX2 and GATA6
• DP cells can contribute to both embryonic epiblast and extra-embryonic endoderm
• In DP cells, SOX2 mobilizes to GATA6 sites, mainly at naive extra-embryonic endoderm enhancers

## Graphical abstract

**Figure:**
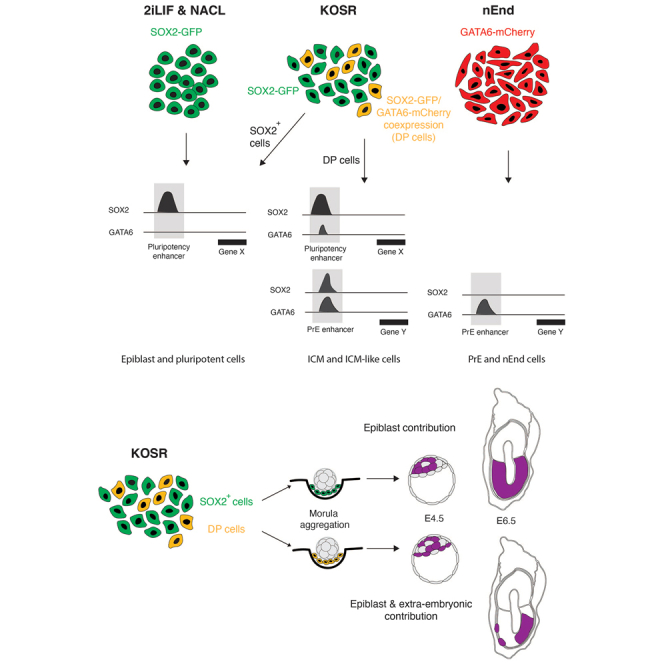

## Teaser

Brickman and colleagues show that an unsegregated ICM-like population arises spontaneously in naive ESC culture in KOSR media. These cells resemble the E3.5 ICM and can contribute to both epiblast and extra-embryonic endoderm. At the chromatin level, SOX2 binds cooperatively with GATA6, and co-bound peaks sit preferentially at endoderm enhancers.

## Introduction

How do progenitor cells sit at the cusp of two lineages, remaining stable as cell types, but simultaneously prepared for differentiation toward multiple fates? In early mammalian embryos, the progenitors of the embryonic epiblast and extra-embryonic primitive endoderm (PrE) stably express antagonistic transcription factors (TFs) that will eventually drive epiblast and PrE lineage specification. Instead of undergoing spontaneous differentiation to both lineages, these cells exist stably across several cell cycles *in vivo* (Dietrich and Hiiragi, 2007). Here, we ask the question of how these cells express antagonistic factors and what function this might have in development and differentiation, focusing on how endoderm and epiblast enhancers become primed in different *in vitro* conditions.

Naive embryonic stem cells (ESCs) are pluripotent cells derived from the inner cell mass (ICM) of the mammalian blastocyst, able to both self-renew and generate all the lineages of the future embryo, but not the extra-embryonic lineages (Morgani et al., 2017; Nichols and Smith, 2011). ESCs can be cultured in a range of conditions, including defined media that support slightly different sub-populations along the spectrum of lineage specification. Culture in serum with the cytokine leukemia inhibitory factor (LIF) or defined basal media supplemented with Activin A, a GSK3 inhibitor (CHIR99021) and LIF (NACL) (Anderson et al., 2017) produce cells primed toward both an epiblast and endoderm identity. By contrast, cells cultured with a MEK inhibitor (PD0325901), CHIR99021 and LIF (2iLIF) (Ying et al., 2008) homogeneously express markers related to epiblast identity and contain a small sub-population that co-express both epiblast protein and extra-embryonic RNA (Morgani et al., 2013). This isolated subpopulation is experimentally totipotent (Riveiro and Brickman, 2020). Another subpopulation shown to exhibit experimental totipotency are 2-cell-like cells, a rare subpopulation that arises spontaneously in ESC culture and expresses factors from the 2-cell (2C) stage embryo (Genet and Torres-Padilla, 2020).

In this paper, we explore how the co-expression of epiblast and PrE factors influence differentiation and TF occupancy. We identify spontaneously arising ESCs that co-express SOX2 and GATA6, where these two factors govern an early ICM-like state. Single cell RNA sequencing (scRNA-seq) revealed that these cells, grown in KnockOut Serum Replacement media (KOSR), are poised for both embryonic and extra-embryonic differentiation. Based on multiple enhancer sets generated from ESC and PrE or naive extra-embryonic endoderm (nEnd) states *in vitro*, we found that SOX2 is recruited to a subset of PrE enhancers and, to a lesser extent, GATA6 to pluripotency enhancers. These findings suggest that the cooperative binding of SOX2 and GATA6 primes enhancer states, potentially setting up competence for differentiation.

## Results

### KOSR promotes early ICM-like cells in culture

**Figure 1:**
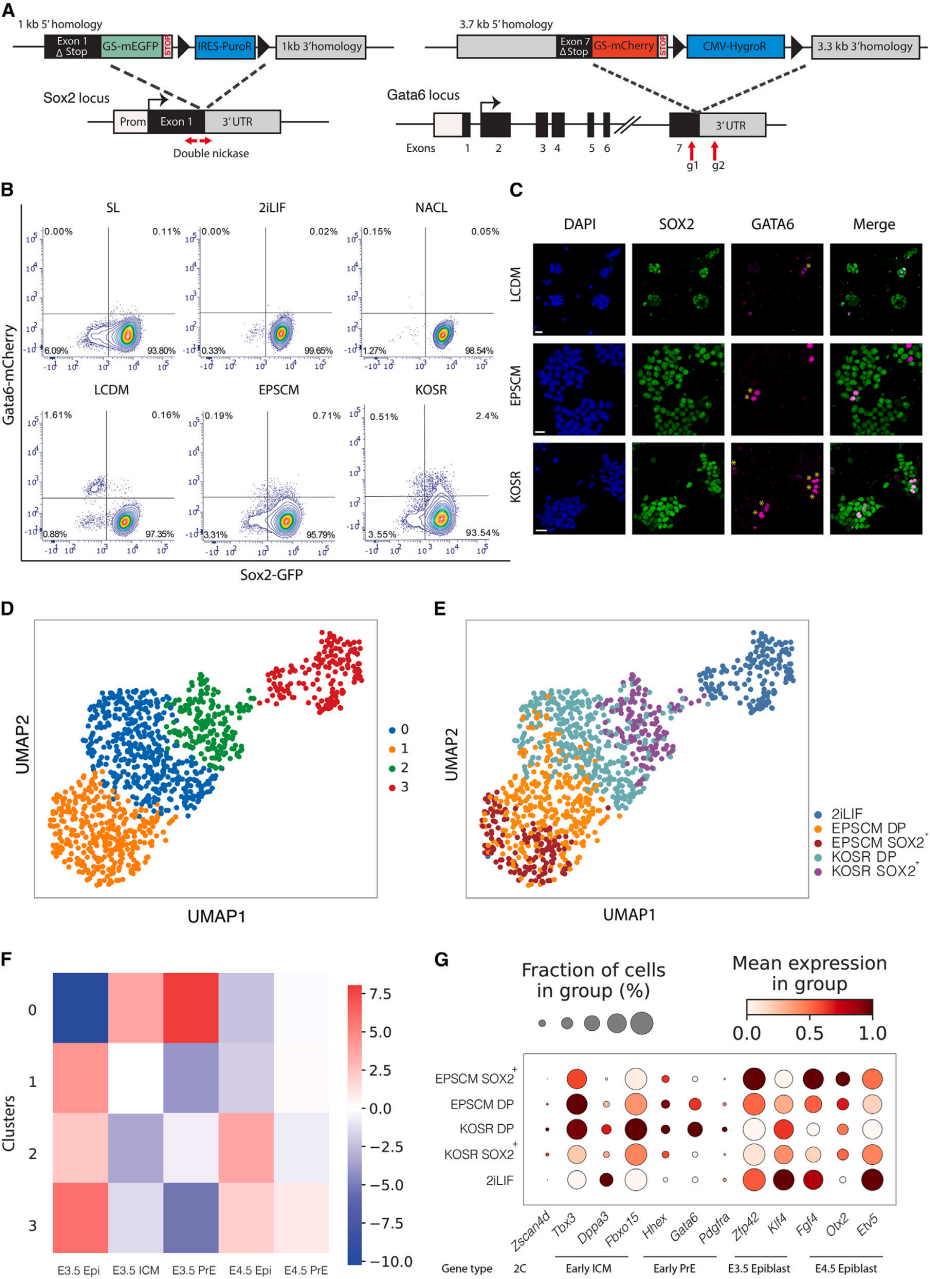
GATA6 and SOX2 expression report on an ICM-like sub-population in certain culture states for naive pluripotency (A) Schematic drawing of the SGGC double reporter. (B) Flow cytometry contour plots of the SGGC cell lines in the stated media conditions. SL, serum/LIF. (C) Immunofluorescence images of SOX2 and GATA6 in the stated conditions. Scale bar, 30 μm, yellow stars indicate co-expression. (D) UMAP of scRNA-seq dataset where coloring represents Louvain clustering. (E) UMAP of scRNA-seq dataset showing the different cell populations sequenced. (F) Heatmap showing normalized residuals post-χ^2^ test comparing differences in proportions of the Seurat clusters mapped to *in vivo* scRNA-seq of pre-implantation blastocysts (Nowotschin et al., 2019). Color scale represents the normalized deviation of observed from expected proportions. (G) Dot plot graph showing the log normalized average expression of selected genes across different media conditions. The average expression is marked by the color scale and the percentage of expression by the size of the circle.

While we had previously described the co-expression of epiblast/ICM TFs with extra-embryonic RNA (Morgani et al., 2013), the co-expression of lineage opposing TFs in ESC culture is rare. To identify conditions that support a co-expressing sub-population, we generated a protein-based double reporter SOX2-GFP/GATA6-mCherry (SGGC) ESC line, with the endogenous epiblast and pluripotency factor SOX2 fused to GFP and the PrE TF GATA6 fused to mCherry (Figures 1A and S1A–S1E). We confirmed that SOX2-GFP was expressed when SGGC ESCs were cultured under naive conditions in 2iLIF and that GATA6-mCherry was expressed following differentiation toward PrE (Anderson et al., 2017) (Figures S1F–S1G). We then explored whether a GATA6/SOX2 double-positive (DP) population could be trapped in different culture conditions, including 2iLIF, NACL, and KOSR (Anderson et al., 2017; Garcia-Gonzalo and Izpisúa Belmonte, 2008; Ying et al., 2008). We also tested two culture conditions reported to produce ESCs with enhanced or expanded potential stem cell media (EPSCM) (Yang et al., 2017a), and extended pluripotent stem cell media containing LIF, CHIR99021, DiM, and MiH (LCDM) (Yang et al., 2017b). Cells cultured in EPSCM and KOSR contained a modest fraction of DP cells (1%–5%), while none of the other culture conditions significantly supported this population (Figure 1B). We confirmed these findings by immunostaining (Figure 1C), indicating that both EPSCM and KOSR cultures can support a small stable DP sub-population.

To determine the nature of DP cells in EPSCM and KOSR and what their equivalent *in vivo* cell state might be, we performed scRNA-seq of SGGC cells in KOSR and EPSCM using MARS-seq2 (Keren-Shaul et al., 2019). Cells were cultured in KOSR, EPSCM or 2iLIF for at least 4 passages. DP and SOX2-GFP single positive (SOX2^+^) cells were isolated by fluorescence-activated cell sorting (FACS) alongside 2iLIF control cells. After pre-processing and quality filtering, our dataset comprised 1,139 cells and 21,590 genes. Using principal component analysis (PCA), the separation of the datasets for PC1 is driven by positive expression of 2C genes (*Zscan4*, *Dux*, and *Tcstv3*), while PC2 is driven by all three culture conditions (Figures S2A and S2B).

To assess the identity of these populations, we visualized the data using Uniform Manifold Approximation and Projection (UMAP) and used unsupervised clustering to define a total of four clusters (Figure 1D). KOSR and EPSCM SOX2^+^ cells cluster independently from each other, with the KOSR SOX2^+^ cells found uniquely in cluster 2, sitting between 2iLIF (cluster 3) and the KOSR DP cells (Figure 1E). KOSR DP cells are all found within a single cluster, cluster 0, whereas ESPSC DP cells are split between clusters 1 and 0 (Figure 1E). Cluster 0 also contains the majority of GATA6-positive cells, a significant proportion of which express *Sox2* mRNA. Pluripotency markers such as *Zfp42* are expressed throughout the four clusters (Figure S2C). To establish the identity of cells and clusters derived in specific culture conditions, we integrated our data with *in vivo* data from the blastocyst (Nowotschin et al., 2019) and found that only cluster 0 resembled the ICM and early PrE (Figure 1F), and, while there are few cells in this cluster from EPSC, 84% of the cells are KOSR DP. While both the EPSCM DP and KOSR DP exhibit some ICM marker expression (Figure S2D), we observed significant upregulation of both early ICM (*Tbx3*, *Dppa3*, and *Fbxo15*) and endoderm (*Gata6*, *Pdgfra*, and *Hhex*) genes specifically in KOSR DP cells compared with EPSCM DP cells (Figures 1G and S2E).

Gene Ontology (GO) analysis of differentially expressed genes between KOSR DP and EPSC DP revealed an enrichment of terms related to metabolism. KOSR DP and EPSCM DP cell transcriptomes were enriched for processes such as hypoxia and mitochondrial activity and pathways related to glycolysis and pyruvate metabolism, respectively (Figures S2F and S2G). In comparison with KOSR SOX2^+^, KOSR DP cells were enriched for genes related to oxidative phosphorylation (e.g., *Cox5a* and *Cox6c*) and lipid metabolism (e.g., *Cpt1a* and *Slc25a20*), as well as regulation of cell death processes and p53 activity (Figures S2H, S2I, and S3A–S3C). Oxidative phosphorylation and lipid metabolism are characteristic of the pre-implantation ICM, which utilizes these energy sources before shifting to a glycolytic metabolism in the later epiblast, peaking at implantation (E4.5–E5.0) (Leese, 2012).

Taken together, these analyses suggest that ESC culture in KOSR best traps a DP SOX2-GATA6 co-expressing population with ICM-like characteristics, and that, of the tested conditions, KOSR is the best candidate for exploring the molecular events that underlie endoderm and epiblast priming *in vivo*.

### KOSR DP cells are dynamic and primed for PrE differentiation

To study the behavior of the DP population in KOSR, we performed live imaging of steady-state KOSR culture for 72 h, a time period sufficient to enable DP cells to arise and revert to single SOX2^+^ or GATA6^+^ cells. Based on lineage tracking of individual cells and their descendants, we found that when DP cells arise, they maintain expression of both GATA6-mCherry and SOX2-GFP for around two cell cycles (∼36 h) (Figure S4A). DP cells divide into distinct daughter cells, with 57% of them maintaining their phenotype, 28% converting to SOX2^+^, and 15% into GATA6^+^ (Figure S4B). The division rate of SOX2^+^ cells is fastest and the GATA6^+^ cells is slowest, with the DP population intermediate between the two, suggesting that these media conditions are optimal for epiblast expansion. Consistent with this, the relative number of cells undergoing cell death is slightly lower in the SOX2^+^ cells (23% in DP vs. 16% in SOX2^+^) (Figure S4C, Video S1).

Video S1. Timelapse of SOX2 and GATA6 expressing ESCs in KOSR culture

Since DP cells (both EPSCM and KOSR DP cells) have an overall longer cell cycle, we determined the distribution of cell cycle stages in the different populations in the scRNA-seq dataset. We observed that the DP populations of both EPSCM and KOSR have more cells in the G1 and G2 phases compared with the control 2iLIF cells and the SOX2^+^ sister cells (Figure S4D). As cells primed for PrE differentiation have been shown to stay for longer in G1 (Coronado et al., 2013; Perera et al., 2022), this could explain why DP cells possess a longer G1 phase. An increase in the G2 phase may be linked to an increase in cell death in the DP cells, as G2 arrest normally precedes apoptosis (Pietenpol and Stewart, 2002).

**Figure 2:**
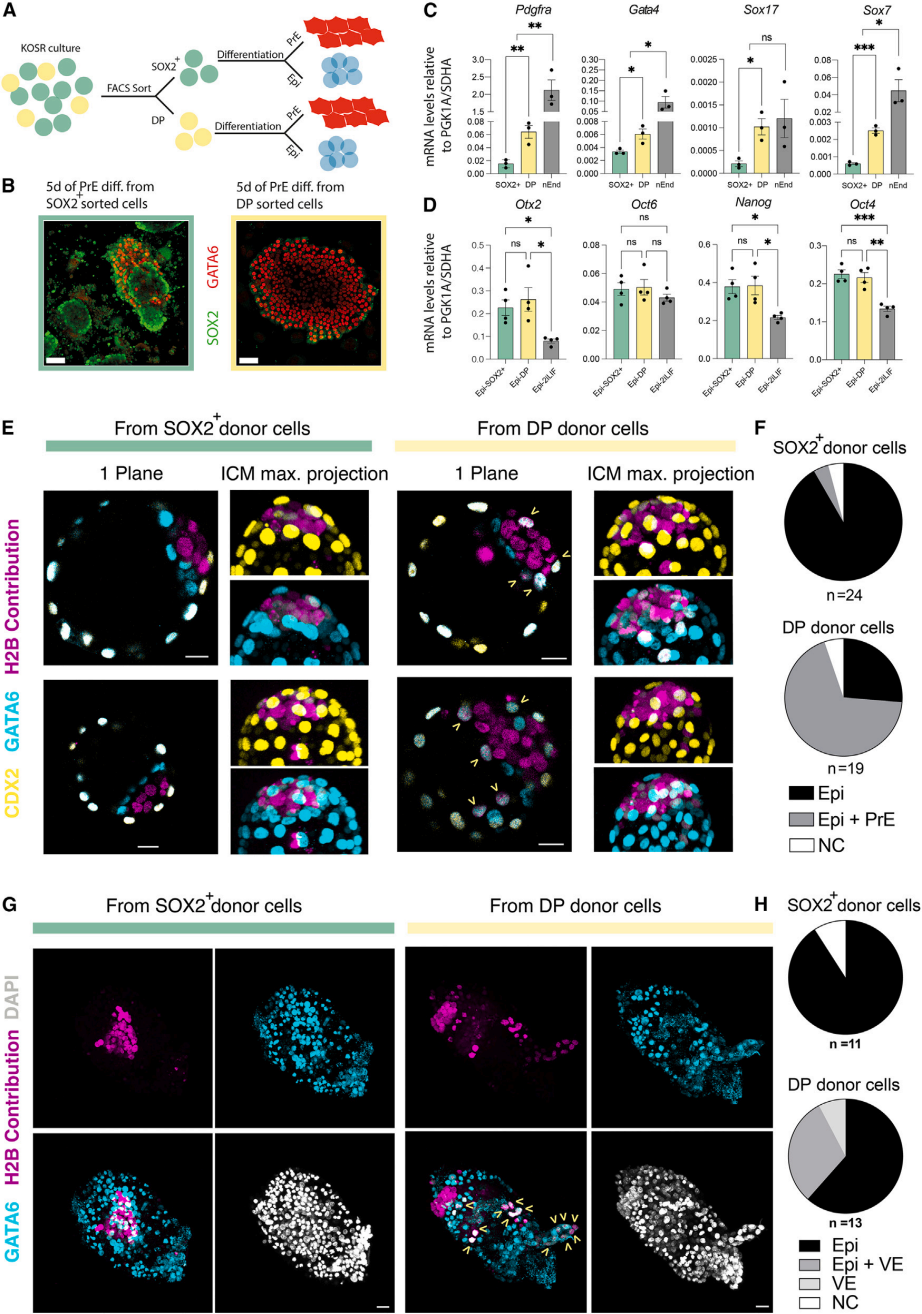
GATA6 and SOX2 DP ESCs have ICM-like bipotency for extra-embryonic endoderm and epiblast differentiation (A) Representative drawing of the PrE and Epi-like differentiations performed after sorting KOSR cells into SOX2^+^ and DP cells by FACS. (B) Representative images of the sorted SOX2^+^ (green frame) and DP (yellow frame) after 5 days (5d) in PrE differentiation media (PrE diff.). Scale bar, 50 μm. (C) Relative mRNA levels of different PrE genes after 5 days of differentiation from sorted SOX2^+^ (green bar) and from DP cells (yellow bar). nEnd control values in gray bar. Dots represent three individual differentiations from different individual clones. Columns show mean ± SEM. Statistics show unpaired t tests. Ns, non-significant. ^∗^p < 0.05; ^∗∗^p < 0.01; ^∗∗∗^p < 0.001. (D) Relative mRNA levels of different Epi genes after 3 days of Epi-like differentiation from sorted SOX2^+^ or DP cells. Dots represent four individual differentiations using two independent clones. Gray columns show control values of four individual Epi-like differentiations starting from a 2iLIF unsorted population. Columns show mean ± SEM. Statistics show one-way ANOVA. ns, non-significant ^∗^p < 0.05; ^∗∗^p < 0.01. (E) Immunofluorescence images showing one plane or a zoomed section of the ICM (maximum projection) of E4.5 blastocysts showing the H2B-tagged contribution from SOX2^+^ or DP donor cells after morula aggregation assay. H2B contribution in magenta, CDX2 staining in yellow, and GATA6 staining in cyan. Scale bar, 20 μm. Arrows show contributing donor cells co-stained with GATA6 and not CDX2, meaning PrE contribution. (F) Quantification of the E4.5 contribution after morula aggregation assay. n = 24 E4.5 blastocysts from SOX2^+^ donor cells and 19 E4.5 blastocysts from DP donor cells. Epi, epiblast contribution; Epi+PrE, epiblast and PrE contribution; NC, no contribution. (G) Immunofluorescence images showing a maximum projection of a representative E6.5 embryo showing the H2B-tagged contribution from SOX2^+^ or DP donor cells after morula aggregation assay. H2B contribution in magenta. GATA6 staining in cyan. Arrows show contributing donor cells co-stained with GATA6 (extra-embryonic VE contribution). DAPI in white. Scale bar, 30 μm. (H) Quantification of the E6.5 contribution after morula aggregation assay. n = 11 E6.5 embryos from SOX2^+^ donor cells and 13 E6.5 embryos from DP donor cells. Epi, epiblast contribution; Epi+VE, epiblast and VE contribution; VE, VE contribution; NC, no contribution.

Given the dynamic nature of the DP population, we reasoned that spontaneously occurring ICM-like cells could represent an intermediate in PrE differentiation that is also capable of giving rise to epiblast. Therefore, ICM-like DP cells should exhibit an enhanced capacity or bias to undergo PrE differentiation, relative to single SOX2^+^ ESCs, but this should not be at the expense of a decrease in the efficiency with which they differentiate to epiblast. To test this hypothesis, we assessed the relative efficiency of KOSR DP and SOX2^+^ cells to differentiate into lineages of the preimplantation embryo (Figures 2A and S4E). When directed toward PrE (Anderson et al., 2017), sorted DP cells rapidly produce robust SOX2^–^/GATA6^+^ monolayers within 5 days, whereas SOX2^+^ cells only partially differentiate (Figures 2B and 2C). In contrast, the epiblast (Figure 2D) or trophectoderm (Figures S4E and S4F) differentiation efficiency of these two populations is not significantly different. Finally, we also found that individual SOX2^+^ and DP cells have a similar capacity to support the expansion of undifferentiated colonies in clonal assays (Figures S4E, S4G, and S4H). Since DP cells can readily differentiate to Epi-like and PrE, mimicking ICM identity, we performed morula aggregation with H2B-miRFP670 tagged SOX2^+^- and DP-sorted cells and analyzed their contribution to host blastocysts. As expected, SOX2^+^ cells extensively contributed solely to the epiblast (22/24 embryos), while DP cells were found in both the epiblast and PrE (13/19 embryos) (Figures 2E and 2F). We transferred aggregates to pseudo-pregnant mice and further assessed the contribution at E6.5. Here, we found that SOX2^+^ donor cells contributed only to the epiblast (10/11 embryos), whereas DP cells contributed to both the extra-embryonic visceral endoderm (VE), marked with GATA6 staining, and epiblast (4/13 Epi and VE, 1/13 VE, and 8/13 Epi contribution) (Figures 2G and 2H). Taken together, these observations support the existence of a transient ICM-like state in KOSR culture.

### Co-expression of SOX2 and GATA6 induces changes to canonical binding

**Figure 3:**
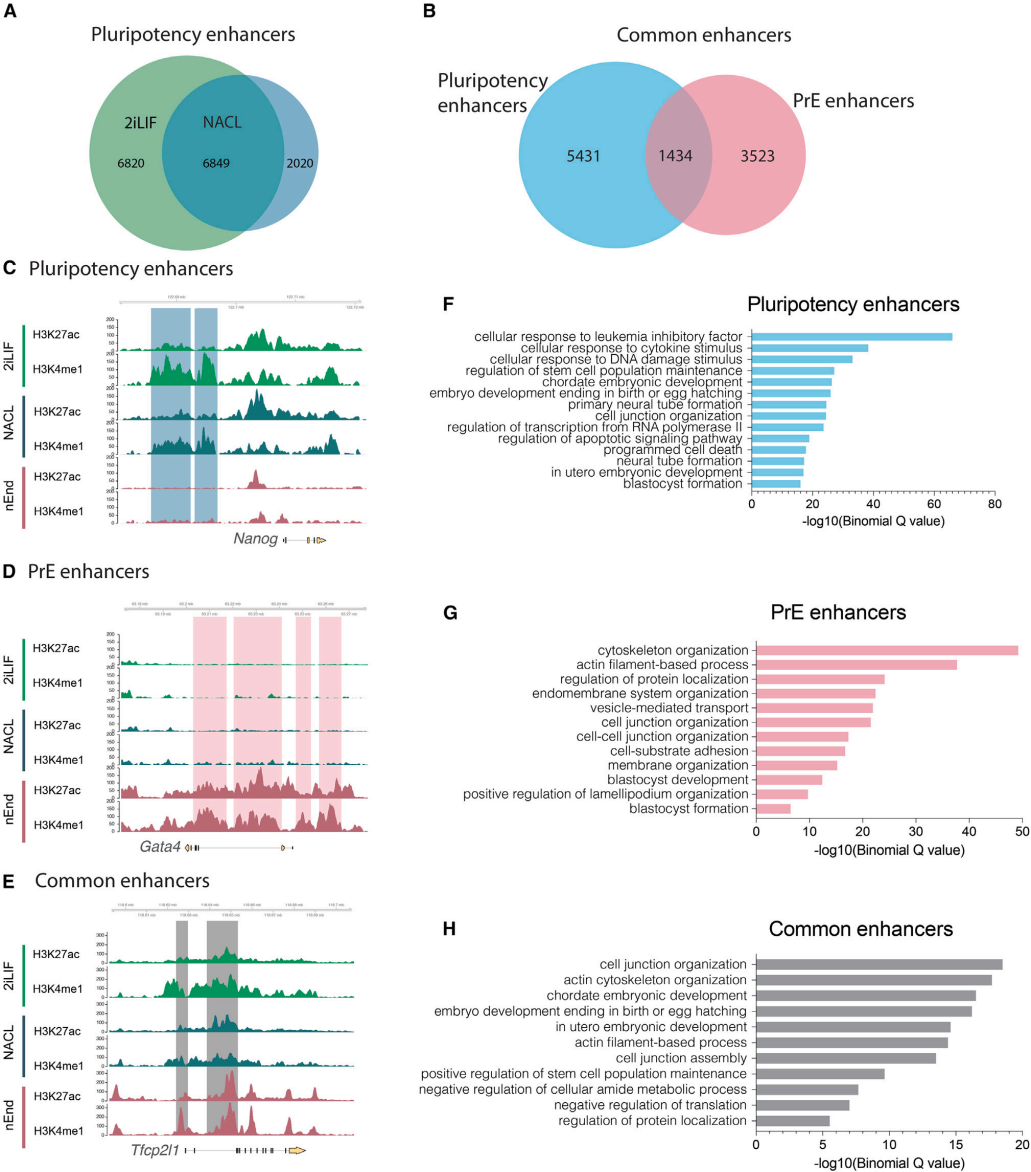
Enhancers governing self-renewing identity in naive endoderm and pluripotency (A) Euler diagram defining an intersect of pluripotency enhancers: enhancers active in both 2iLIF and NACL. (B) Euler diagram comparing pluripotency enhancers (intersect from A) with PrE enhancers. (C–E) Genome browser tracks of H3K27ac and H3K4me1 across conditions 2iLIF, NACL, and nEnd of loci *Nanog* (C), *Gata4* (D), and *Tfcp2l1* (E) with examples of pluripotency enhancers (blue), PrE enhancers (pink), and common enhancers (gray) defined in (B). (F–H) GO terms of the biological processes of pluripotency enhancers (F), PrE enhancers, (G) and common enhancers (H).

To determine how these two antagonistic TFs could be co-expressed in KOSR and whether they might influence each other’s binding or activity, we sought to identify the extent to which they recognize the same target in different populations. While considerable effort has been invested into understanding the pluripotency or epiblast network (Li and Belmonte, 2017), by comparison, little has been invested into PrE. To provide a framework by which we can understand the nature of this lineage bifurcation, we first established the enhancer network in differentiated PrE cells and compared it with the epiblast, as recapitulated in different pluripotent culture conditions. We assessed the enhancer network in nEnd stem cells and compared it with two distinct pluripotent conditions: 2iLIF and NACL. NACL is a defined pluripotent culture system where cells exhibit similar heterogeneity as conventional serum-containing media. In addition, NACL media is composed of the same set of cytokines as used for nEnd culture, but differs only in its base media (N2B27). We identified cell-type-specific enhancers by profiling the co-occupancy of the histone modifications H3K27ac and H3K4me1, the combination of which denotes enhancer activity (Calo and Wysocka, 2013) using Cleavage Under Targets & Release Using Nuclease (CUT&RUN) (Skene and Henikoff, 2017) (Figures 3A–3E). Based on the combination of these marks in two clonal cell lines in both pluripotent conditions, we identified 6,849 active pluripotency enhancers (Figures 3A–3C). In nEnd, we found 4,957 active PrE enhancers, with 1,434 of these being shared by both lineages, referred to as “common enhancers” (Figures 3B, 3D, and 3E). Motif analysis indicated that these regulatory regions are cell type specific as we identified specific groups of motifs enriched in each enhancer set. We observed that the PrE subset was highly enriched for GATA motifs, while the pluripotency subset was enriched for the OCT, NANOG, ESRRB, and KLF motifs. The common enhancer subset features a strong KLF signature, consistent with its expression in both lineages (Morgani and Brickman, 2015; Nowotschin et al., 2019) (Figure S4I). GO analysis for Biological Processes for the closest gene to the different enhancer subsets suggest that pluripotency enhancers were associated with LIF response and embryo development terms (Figure 3F), the PrE enhancers with membranes and adhesion (Figure 3G), and common enhancers represented general cellular processes (Figure 3H).

**Figure 4:**
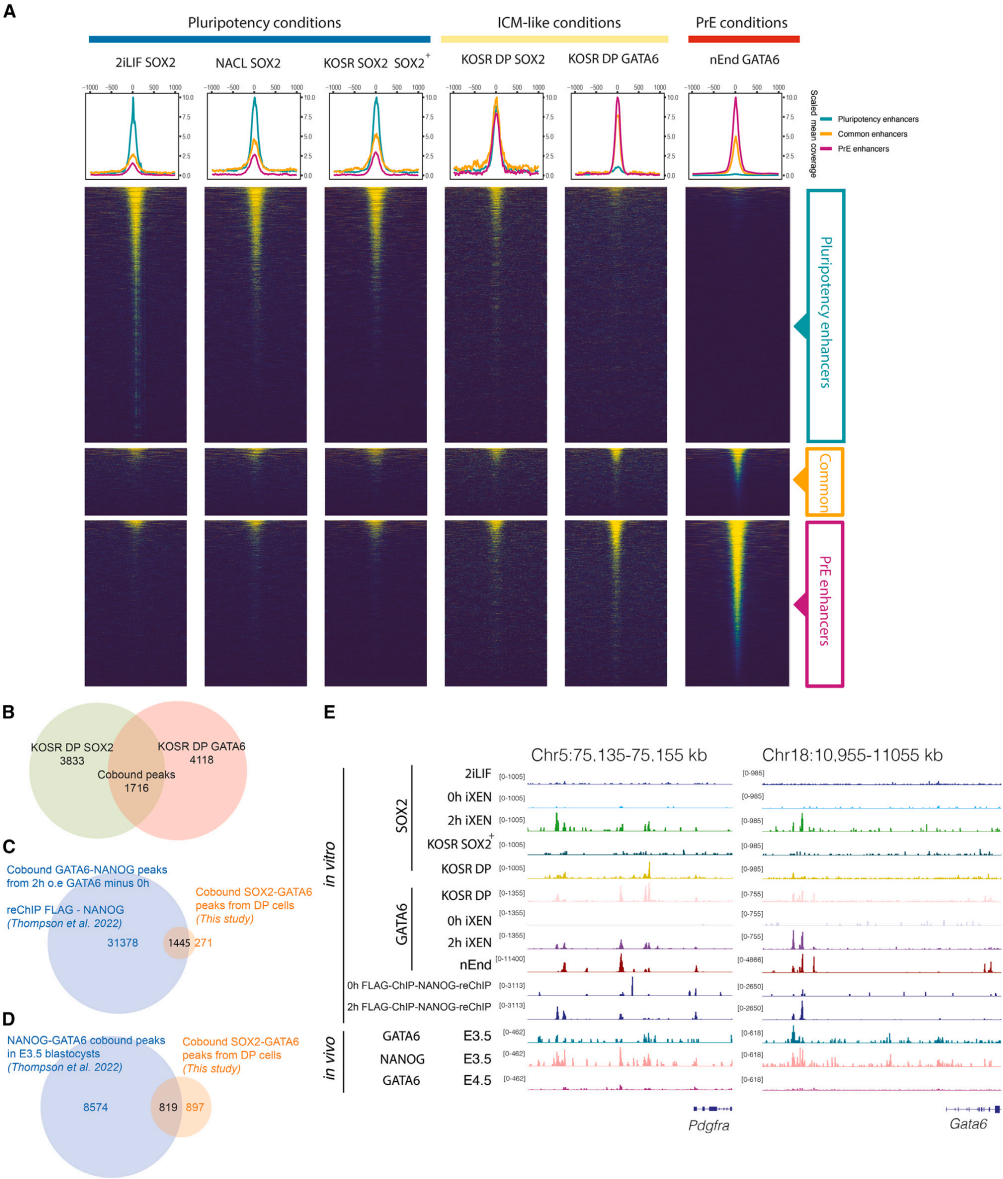
Reciprocal lineage priming by GATA6 and SOX2 (A) Scaled heatmaps showing SOX2 and GATA6 binding at the pluripotency, common, or PrE enhancer subsets defined previously, in the conditions: 2iLIF, NACL, KOSR sorted cells, and nEnd. (B) Euler diagram of SOX2 and GATA6 peaks in KOSR DP cells. (C) Euler diagram showing the overlap of the GATA6-FLAG chromatin immunoprecipitation (ChIP) with NANOG reChIP (Thompson et al. 2022) with our SOX2-GATA6 co-bound peaks in DP cells. (D) Euler diagram projecting the NANOG-GATA6 co-bound peaks from the *in vivo* data of the E3.5 blastocysts (Thompson et al. 2022) with the SOX2-GATA6 co-bound peaks in KOSR DP cells. (E) Genome browser tracks of SOX2 and GATA6 comparing data from this study (2iLIF, KOSR, and nEnd tracks) with Thompson et al. (2022) (0 h and 2 h of iXEN induction, the GATA-FLAG ChIP with NANOG reChIP and the *in vivo* E3.5 and E4.5 blastocysts tracks). *Pdgfra* and *Gata6* loci. Tracks are scaled accordingly with antibody and experiment. All the *in vitro* SOX2 experiments are in the same scale, the *in vitro* GATA6 experiments are also in the same scale (apart from nEnd due to the extreme difference in PrE genes expression). FLAG-NANOG reChIP tracks are on the same scale, as well as the *in vivo* data.

Having established the enhancer networks in both lineages, we assessed TF binding at both enhancer sets in both the final cell states (ESC and nEnd) and in the DP and in SOX2^+^ populations cultured in KOSR. CUT&RUN was used to profile GATA6 in nEnd and DP populations and SOX2 to analyse NACL, 2iLIF and SOX2+ populations. To determine if SOX2 and GATA6 binding shifts globally, we compared SOX2 and GATA6 binding on pluripotency, PrE, and common enhancer sets (Figure 4A). While we observed a decrease in SOX2 binding to pluripotency enhancers in DP cells, we observed a significant recruitment of SOX2 to PrE enhancers alongside GATA6, suggesting that SOX2 binding moves toward potential GATA6 sites. We also observed a smaller acquisition of GATA6 peaks at pluripotency enhancers, slightly greater than GATA6 binding the same elements in nEnd (Figure 4A). We detect 1,716 peaks co-bound by SOX2 and GATA6 in the DP cells (Figure 4B), and 416 of these peaks (24%) sit at enhancers that we previously defined. The major cluster of co-bound peaks at enhancers (50%) sit at PrE enhancers, while only 28% of these are found at pluripotency enhancers and 22% at common enhancers. Taken together, this suggests that SOX2 is recruited to sites with GATA6 occupancy; when this takes place at an enhancer, it is predominantly at PrE enhancers. Given the PrE bias in occupancy, we analyzed the closest genes regulated *in vivo* (Boroviak et al., 2018) to these SOX2-GATA6 co-bound peaks and observed a 2-fold enrichment of PrE genes over epiblast genes. Moreover, the co-bound regions, regardless of their affiliation to lineage specific genes, contain twice as many GATA6 motifs as those found for SOX2 (Tables S1 and S2). Only around 10% of the closest genes to the co-bound peaks are significantly upregulated when DP cells are compared with SOX2^+^ single-positive cells (specifically, 4.9% for epiblast genes and 11.7% for PrE genes) (Tables S1 and S2), suggesting that lineage priming is not occurring at the transcriptional level.

While our data on co-binding is not based on sequential precipitation, we took advantage of a recent study that explored a similar co-binding phenomena by sequential chromatin immunoprecipitation in response to GATA6 induction in ESCs to drive differentiation to iXEN cells (Thompson et al., 2022). We observed a significant percentage of overlap of our binding for SOX2 and GATA6 in DP cells with this study’s early timepoints for GATA6 induction (Figure 4E; and Table S3). Here, we found that between 70% and 80% of our DP peaks overlap with the 2- and 4-h overexpression time points for the factor GATA6 and 50%–60% for SOX2. We observe an impressive 84% overlap of our co-bound peaks with the NANOG-GATA6 co-bound peaks at 2 h based on sequential precipitation (GATA6-FLAG-NANOG) (Figures 4C and 4E), suggesting that our SOX2 and GATA6 are indeed binding at the same sites. We also observe a 48% overlap of our DP GATA6-SOX2 co-bound peaks with their NANOG-GATA6 co-bound peaks in the *in vivo* E3.5 blastocysts (Figure 4D). Moreover, assessment of these data also suggests that the preference of SOX2/GATA6 binding for PrE enhancers in our data may be a general property of cooperative binding between endoderm and epiblast determinants, as *in vivo* ICM GATA6-NANOG co-bound peaks also appear to more often bind in our endoderm enhancers (48%) compared with their presence at pluripotency ones (33%).

## Discussion

In this paper, we found that ICM-like expression of endoderm and epiblast TFs prepare differentiation not only for their own lineage, but for the opposing lineage as well. Cooperative binding interactions between SOX2 and GATA6 simultaneously prepare enhancers for activation, while maintaining these genes in a primed status enables rapid response in lineage bifurcation. Multipotent progenitors must maintain tight control of lineage commitment to ensure correctly proportioned embryonic development. Lineage-specific inducer genes, such as *Gata6* and *Sox2*, which are co-expressed in early ICM cells (Dietrich and Hiiragi, 2007; Morgani and Brickman, 2015), could prime later differentiation based on their ability to stabilize each other’s binding and maintain cells with the ability to kick start either lineage-specific gene regulatory network.

DP cells can readily differentiate toward epiblast or PrE, while their sister cells in culture (SOX2^+^ cells) seem to be biased toward epiblast. Transcriptionally and metabolically, DP cells also better approximate the E3.5 unsegregated ICM, whereas the SOX2^+^ cells resemble the E4.5 epiblast. In agreement with Posfai et al. (2021), we observe that cells cultured in EPSCM have a more epiblast-like signature. Given that the ICM can sustain the co-expression of these factors over multiple cell divisions, the wiring of these cooperative interactions between cross-lineage TFs are likely to be stable; we find *in vitro* ICM-like cells can sustain this state through cell division as well. A recent study based on GATA6 over-expression describes an *in vitro* model for the ICM based on early time points of GATA6-mediated reprogramming of naive ESCs (Thompson et al., 2022). However, here we manage to capture a small population of cells that spontaneously enter this state in self-renewing culture without artificial manipulation of gene expression. The ability to sort this population at a steady state enabled us to correlate differentiation competence with TF co-occupancy. Further work is required to properly understand the ICM’s environment, which in turn will lead to better ways to robustly expand these cells *in vitro*. But even as a small population, these cells seem to represent a genuine stem cell model that indefinitely maintains embryonic and extra-embryonic bipotency.

Steady-state culture pluripotency is supported by a set of enhancers that contain motifs for pluripotency factors, such as OCT4, SOX2, NANOG, KLFs, and ESRRB, while in nEnd the enhancers feature mostly GATA motifs. Common enhancers, which have H3K27ac and H3K4me1 present in both nEnd and naive cultures, are enriched for KLF motifs. While we observe GATA6 and SOX2 binding to all these enhancer sets, we find an almost identical bias in our data as that derived from the E3.5 ICM *in vivo* (Thompson et al., 2022) for PrE enhancers, as well as 2-fold increase in GATA6 motifs over SOX2 motifs. This suggests that GATA6 binds its consensus sites with a relatively high affinity and actively recruits SOX2, possibly via protein-protein interaction, rather than the other way round. This would seem to contrast its role in ESCs, where residence time data and *in vivo* imaging studies suggest that SOX2 drives OCT4 binding (Chen et al., 2014; White et al., 2016). Alternatively, this might reflect the ability of SOX2 to recognize lower affinity elements, like those recognized by SOX17, only in the presence of GATA6. Moreover, we observed the presence of SOX sites in the proximity of GATA in the PrE, but not the other way round. However, if this is the case, why should the presence of GATA6 still facilitate both endoderm and epiblast differentiation? Perhaps the relative level of free SOX2 is a key determinant of epiblast differentiation, and the presence of GATA6 titrates SOX2 away from epiblast enhancers and OCT4, maintaining a threshold concentration that can be pushed toward either endoderm or epiblast.

In hematopoietic differentiation, the co-expression of antagonistic lineage specifiers is thought to maintain progenitor populations at the apex of two lineages in a process referred to as multi-lineage priming (Graf and Enver, 2009; Hu et al., 1997). Here we find that cooperativity between GATA6 and SOX2 leads to alterations in their binding in DP cells, such that they are sitting at sites found in both lineage-specific enhancer sets. Presumably, there are either insufficient levels of these factors to drive differentiation or perhaps the expression of these two TFs in the absence of lineage determining signaling is insufficient to promote differentiation (Hamilton et al., 2019; Knudsen and Brickman, 2020). As there is little transcription of genes close to co-bound peaks, lineage-specific signaling may be required to drive transcription and occupancy by both TFs creates a situation in which the genes they regulate are in a ready response mode. That TFs bind to enhancers and prepare them for transcription in response to signaling to encode potency or remain bound following a signal to safeguard plasticity suggests that they are determinants of potential rather than actuality (Hamilton et al., 2019; Knudsen et al., 2023; Wong et al., 2023). In this instance, these factors bind together to enhancers that can drive transcription in either cell type, providing a head start for either lineage, but without triggering differentiation. Thus, multi-lineage priming may be about manipulating threshold distributions for lineage specification, exploiting antagonistic factors with interactions that can be manifest at the level of cooperative binding. In this way, these factors support cells in a precarious balance poised for either of two opposing fates that can be readily induced by alterations to signaling that promote lineage specific transcription.

## Experimental procedures

### Resource availability

#### Corresponding author

Further information and requests for resources and reagents should be directed to the corresponding author Joshua M Brickman joshua.brickman@sund.ku.dk.

#### Materials availability

Reagents generated in this study are available upon reasonable request to the corresponding author.

#### Data and code availability

scRNA-seq and CUT&RUN data that support the findings of this study have been deposited in NCBI GEO under accession number GSE227889 (https://www.ncbi.nlm.nih.gov/geo/query/acc.cgi?acc=GSE227889).

The full scRNA-seq analysis can be found at https://github.com/brickmanlab/riveiro-et-al-2023/.

Previously published Nowotschin et al., 2019 and Thompson et al., 2022 datasets that were used here are available in the NCBI GEO under accession numbers GSE123046 and GSE181104.

### ESC culture

ESCs were generated using E14JU ESCs from the 129/Ola background. ESC lines were maintained in serum/LIF Canham et al. (2010), 2iLIF (Ying et al., 2008), NACL (Anderson et al., 2017), KOSR (Martin Gonzalez et al., 2016), EPSCM (Yang et al., 2017a), and LCDM (Yang et al., 2017b) as previously described.

### Generation of SGGC ESC lines

SOX2-GFP ESCs (Anderson et al., 2017) clone SG16 was used to further target with a GATA6-mCherry construct using CRISPR-Cas technology. The construct contains mCherry tagged immediately after exon 7 of the *Gata6* locus, just before the STOP codon, plus 3,000-bp homology arms. We obtained three clones (B9, B12 and E1) that were successfully integrated.

SGGC cells were verified by performing immunostaining for SOX2, GATA6, mCherry and GFP, western blot, locus Sanger sequencing to screen for unwanted mutations generated by CRISPR, karyotyping, and Southern blot (Figures S1A–S1E). Resistance cassettes can be easily removed using Cre-mediated recombination; however, we decided to use the original clones with the resistance cassettes included. All three clones give the same reproducible results. All three clones were used for scRNA-seq. Clones B9 and B12 presented the highest amount of DP cells, so these two clones were used in all CUT&RUN, differentiation, and morula aggregation experiments. Detailed characterization of the SGGC cell line is described in the figure legends and supplemental information.

### Flow cytometry and FACS

Cells were analyzed using an LSR Fortessa flow cytometer (BD Biosciences) with FACSDiva (BD Biosciences) software. Plots were generated using FCS Express 6.0 (DeNovo Software). Cells were sorted by SOX2^+^ or DP populations using a BD FACS Aria III (BD FACSDiva Software version 8) with a 100-μm nozzle. Further details are contained in the supplemental information.

### Differentiation

ESCs were cultured for four passages in KOSR prior to differentiation, isolated by FACS for DP or SOX2^+^ expression, and seeded at the same cell density. Upon attachment, media was replaced for specific differentiation conditions. For nEnd differentiation, we plated 6 × 10^4^ cells/cm^2^ in gelatinized plates and cultured the cells in RACL media as previously described (Anderson et al., 2017; Linneberg-Agerholm et al., 2019). For Epi-like differentiation, we plated 20 × 10^4^ cells/cm^2^ in fibronectin-coated plates and cultured the cells in Epi-like media for 3 days (Hayashi et al., 2011).

For trophoblast stem cell differentiation, we plated 10 × 10^4^ cells/cm^2^ and then transferred to trophoblast stem cell medium for a total of 6 days (Tanaka et al., 1998). Antibody staining, RT-PCR, and alkaline phosphatase are described in the supplemental information.

### scRNA-seq

Cells were sorted directly into 384-well plates containing lysis buffer, which includes the first RT primer and RNase inhibitor, then immediately frozen and later processed by the MARS-seq2 protocol (Keren-Shaul et al., 2019). scRNA-seq libraries were sequenced using Illumina NextSeq500 at a median sequencing depth of 225,000 reads per single cell. Pre-processing was done using the nfcore/marsseq pipeline (Proks et al., 2023) with the following command: nextflow run nf-core/marsseq -r 1.0.3 -profile ku_sund_dangpu –with-tower --genome mm10 --velocity --input SCR_20221006/raw/samplesheet.csv --outdir/scratch/ALBA_SB2/.

Both *in vivo* (Nowotschin et al., 2019) and *in vitro* datasets were independently processed using SCANPY (v1.9.3) (Wolf et al., 2018). The MARS-seq2 *in vitro* dataset was filtered to include cells containing between 1,000 and 45,000 unique molecular identifiers (UMIs) representing between 1,400 and 7,500 genes. The reference *in vivo* dataset was filtered to exclude cells with fewer than 10 UMIs and 10 genes and to only contain cells annotated as originating from E3.5 and E4.5 mouse stages. Empty control wells labeled as zero and ERCC-genes were also discarded. The filtered *in vitro* and *in vivo* datasets contained 1,139 cells and 1,006 cells, respectively. Raw counts were then depth normalized and Log1p transformed. For downstream analysis, highly variable genes were identified and a reduced dimension UMAP representation was computed for first 30 PCAs, followed by Leiden unsupervised clustering, which estimated four clusters with a set resolution of 0.4. The top differentially expressed genes were identified using a Benjamini-Hochberg-corrected t test, with the following cutoffs (log_2_(fold change) > 1 and adjusted p < 0.05). Mapping of the *in vitro* data onto the *in vivo* mouse E3.5 and E4.5 dataset was performed using the ‘ingest’ function of SCANPY. PCA of this dataset, and subsequent UMAP dimension reduction, was performed using an intersection of the top highly variable genes. The χ^2^ test, implemented in SciPy (Virtanen et al., 2020), was used to determine significance for differences in observed proportions of experimental conditions or clusters. The seaborn library (Waskom, 2021) was used for visualization of normalized residuals.

### Time-lapse imaging and cell tracking

SGGCH2B ESC lines (SGGC lines tagged with H2B-miRFP670) were cultured in KOSR media, on 8-well slides (Ibidi) and imaged every 15 min across 72 h. mCherry, GFP, and CY5 fluorescent light channels were recorded in 5% CO_2_, 20% O_2_ at 37°C under a Deltavision Widefield Screening microscope. ESCs were seeded at 5,000 cells/cm^2^ 24 h before the beginning of the time lapse in KOSR. We performed manual cell tracking using Imaris v9.5 (Bitplane). Nuclei were segmented using the H2B marker. We measured the SOX2-GFP and GATA6-mCherry fluorescence intensities of a circular area of a 50-μm diameter inside the segmented nuclei. For each area measured, we took the median fluorescence intensity as the measure for that given data point. Intensity measurements were linked to its time point and lineage, allowing us to infer the division time for each cell that was tracked, as well as the expression level of both SOX2 and GATA6 in each time point. Only cells with completed cell cycle information were used for calculating the transition analysis. A total of 63 individual tracks of 72 h have been tracked.

### CUT&RUN

KOSR, nEnd, 2iLIF, or NACL cells were grown in their respective media for at least four passages. A minimum of 100,000 cells were sorted to proceed with the CUT&RUN protocol. CUT&RUN was performed using an in house purified MNase and following the published protocol (Janssens and Henikoff, 2019). Library preparation was performed following this published protocol (Liu, 2019).

Reads were pre-processed using Cutadapt (Martin, 2011), Bowtie2 (Langmead and Salzberg, 2012), PICARD 2, SAMtools (Li et al., 2009), BEDtools (Quinlan and Hall, 2010), and DeepTools (Ramírez et al., 2016). All downstream data analysis was performed using BEDtools, SAMtools, DeepTools, Fluff (Georgiou and van Heeringen, 2016), Integrative Genome Viewer (IGV 2.16.0) (Robinson et al., 2011), SEACR (Meers et al., 2019), HOMER (Heinz et al., 2010), and RStudio (RStudio, 2016). Peaks were called using SEACR with the parameters: ‘relaxed’ for TFs and ‘Stringent’ for histone marks. Peaks were called against a negative IgG control, generated in each experiment for each condition. Further details can be found in the supplemental methods.

### Chimera assays

For chimera assays, H2B-miRFP670 tagged clones SGGCH2B B9.A and B12.B were used. Cells were sorted for SOX2^+^ or DP as previously described. Further details about morula aggregation protocol can be found in supplemental methods. Animal work was carried in accordance with European legislation. All work was authorized by and carried out under Project License 2018-15-0201-01520 and 2023-15-0201-01513 issued by the Danish Regulatory Authority.

## Supplemental information

Document S1. Supplemental experimental procedures and Figures S1–S4

Table S1. SOX2 and GATA6 binding Table shows a list of the closest Epi genes to the co-bound peaks for SOX2 and GATA6 in DP cells. It also shows how many SOX2 and GATA6 motifs we find in these regions. Genes colored in yellow are differentially upregulated in DP over SOX2+ cells in the scRNA-seq. The ones colored in green are differentially upregulated in SOX2+ over DP cells.

Table S2. Characteriza5on of DP SOX2 and GATA6 co-bound regions Table shows a list of the closest PrE genes to the co-bound peaks for SOX2 and GATA6 in DP cells. It also shows how many SOX2 and GATA6 motifs we find in these regions. Genes colored in yellow are differentially upregulated in DP over SOX2+ cells in the scRNA-seq. The ones colored in green are differentially upregulated in SOX2+ over DP cells.

Table S3. Comparison of this study to Thompson et al. 2022 Table compares SOX2 and GATA6 binding peaks from our study with the SOX2 and GATA6 peaks of the GATA6 overexpression time course from Thompson et al., (2022).

Document S2. Article plus supplemental information

## Footnotes

Supplemental information can be found online at https://doi.org/10.1016/j.stemcr.2023.12.002.

## References

[bib1] Anderson K.G.V., Hamilton W.B., Roske F.V., Azad A., Knudsen T.E., Canham M.A., Forrester L.M., Brickman J.M. (2017). Insulin fine-tunes self-renewal pathways governing naive pluripotency and extra-embryonic endoderm. Nat. Cell Biol..

[bib3] Boroviak T., Stirparo G.G., Dietmann S., Hernando-Herraez I., Mohammed H., Reik W., Smith A., Sasaki E., Nichols J., Bertone P. (2018). Single cell transcriptome analysis of human, marmoset and mouse embryos reveals common and divergent features of preimplantation development. Dev. Camb. Engl..

[bib4] Calo E., Wysocka J. (2013). Modification of Enhancer Chromatin: What, How, and Why?. Mol. Cell.

[bib5] Canham M.A., Sharov A.A., Ko M.S.H., Brickman J.M. (2010). Functional Heterogeneity of Embryonic Stem Cells Revealed through Translational Amplification of an Early Endodermal Transcript. PLoS Biol..

[bib6] Chen J., Zhang Z., Li L., Chen B.-C., Revyakin A., Hajj B., Legant W., Dahan M., Lionnet T., Betzig E. (2014). Single-Molecule Dynamics of Enhanceosome Assembly in Embryonic Stem Cells. Cell.

[bib7] Coronado D., Godet M., Bourillot P.-Y., Tapponnier Y., Bernat A., Petit M., Afanassieff M., Markossian S., Malashicheva A., Iacone R. (2013). A short G1 phase is an intrinsic determinant of naïve embryonic stem cell pluripotency. Stem Cell Res..

[bib8] Dietrich J.-E., Hiiragi T. (2007). Stochastic patterning in the mouse pre-implantation embryo. Dev. Camb. Engl..

[bib9] Garcia-Gonzalo F.R., Izpisúa Belmonte J.C. (2008). Albumin-Associated Lipids Regulate Human Embryonic Stem Cell Self-Renewal. PLoS One.

[bib10] Genet M., Torres-Padilla M.-E. (2020). The molecular and cellular features of 2-cell-like cells: a reference guide. Dev. Camb. Engl..

[bib11] Georgiou G., van Heeringen S.J. (2016). fluff: exploratory analysis and visualization of high-throughput sequencing data. PeerJ.

[bib12] Graf T., Enver T. (2009). Forcing cells to change lineages. Nature.

[bib13] Hamilton W.B., Mosesson Y., Monteiro R.S., Emdal K.B., Knudsen T.E., Francavilla C., Barkai N., Olsen J.V., Brickman J.M. (2019). Dynamic lineage priming is driven via direct enhancer regulation by ERK. Nature.

[bib14] Hayashi K., Ohta H., Kurimoto K., Aramaki S., Saitou M. (2011). Reconstitution of the Mouse Germ Cell Specification Pathway in Culture by Pluripotent Stem Cells. Cell.

[bib15] Heinz S., Benner C., Spann N., Bertolino E., Lin Y.C., Laslo P., Cheng J.X., Murre C., Singh H., Glass C.K. (2010). Simple Combinations of Lineage-Determining Transcription Factors Prime cis-Regulatory Elements Required for Macrophage and B Cell Identities. Mol. Cell.

[bib16] Hu M., Krause D., Greaves M., Sharkis S., Dexter M., Heyworth C., Enver T. (1997). Multilineage gene expression precedes commitment in the hemopoietic system. Genes Dev..

[bib17] Janssens D., Henikoff S. (2019).

[bib18] Keren-Shaul H., Kenigsberg E., Jaitin D.A., David E., Paul F., Tanay A., Amit I. (2019). MARS-seq2.0: an experimental and analytical pipeline for indexed sorting combined with single-cell RNA sequencing. Nat. Protoc..

[bib19] Knudsen T.E., Brickman J.M. (2020). Can a Cell Put Its Arms around a Memory?. Cell Stem Cell.

[bib20] Knudsen T.E., Hamilton W.B., Proks M., Lykkegaard M., Linneberg-Agerholm M., Nielsen A.V., Perera M., Malzard L.L., Trusina A., Brickman J.M. (2023). A bipartite function of ESRRB can integrate signaling over time to balance self-renewal and differentiation. Cell Syst..

[bib21] Langmead B., Salzberg S.L. (2012). Fast gapped-read alignment with Bowtie 2. Nat. Methods.

[bib22] Leese H.J. (2012). Metabolism of the preimplantation embryo: 40 years on. Reproduction.

[bib23] Li M., Belmonte J.C.I. (2017). Ground rules of the pluripotency gene regulatory network. Nat. Rev. Genet..

[bib24] Li H., Handsaker B., Wysoker A., Fennell T., Ruan J., Homer N., Marth G., Abecasis G., Durbin R., 1000 Genome Project Data Processing Subgroup (2009). The Sequence Alignment/Map format and SAMtools. Bioinformatics.

[bib25] Linneberg-Agerholm M., Wong Y.F., Herrera J.A.R., Monteiro R.S., Anderson K.G.V. (2019). Naïve human pluripotent stem cells respond to Wnt, Nodal and LIF signalling to produce expandable naïve extra-embryonic endoderm. Development.

[bib26] Liu N. (2019).

[bib27] Martin M. (2011). Cutadapt removes adapter sequences from high-throughput sequencing reads. EMBnet. j..

[bib28] Martin Gonzalez J., Morgani S.M., Bone R.A., Bonderup K., Abelchian S., Brakebusch C., Brickman J.M. (2016). Embryonic Stem Cell Culture Conditions Support Distinct States Associated with Different Developmental Stages and Potency. Stem Cell Rep..

[bib29] Meers M.P., Tenenbaum D., Henikoff S. (2019). Peak calling by Sparse Enrichment Analysis for CUT&RUN chromatin profiling. Epigenet. Chromatin.

[bib30] Morgani S.M., Brickman J.M. (2015). LIF supports primitive endoderm expansion during pre-implantation development. Development.

[bib31] Morgani S.M., Canham M.A., Nichols J., Sharov A.A., Migueles R.P., Ko M.S.H., Brickman J.M. (2013). Totipotent Embryonic Stem Cells Arise in Ground-State Culture Conditions. Cell Rep..

[bib32] Morgani S., Nichols J., Hadjantonakis A.-K. (2017). The many faces of Pluripotency: *in vitro* adaptations of a continuum of *in vivo* states. BMC Dev. Biol..

[bib33] Nichols J., Smith A. (2011). The origin and identity of embryonic stem cells. Development.

[bib34] Nowotschin S., Setty M., Kuo Y.-Y., Liu V., Garg V., Sharma R., Simon C.S., Saiz N., Gardner R., Boutet S.C. (2019). The emergent landscape of the mouse gut endoderm at single-cell resolution. Nature.

[bib35] Perera M., Nissen S.B., Proks M., Pozzi S., Monteiro R.S., Trusina A., Brickman J.M. (2022).

[bib36] Pietenpol J.A., Stewart Z.A. (2002). Cell cycle checkpoint signaling:: Cell cycle arrest versus apoptosis. Toxicology.

[bib37] Posfai E., Schell J.P., Janiszewski A., Rovic I., Murray A., Bradshaw B., Yamakawa T., Pardon T., El Bakkali M., Talon I. (2021). Evaluating totipotency using criteria of increasing stringency. Nat. Cell Biol..

[bib38] Proks M., Herrera J.A.R., Sedzinski J., Brickman J.M. (2023).

[bib39] Ramírez F., Ryan D.P., Grüning B., Bhardwaj V., Kilpert F., Richter A.S., Heyne S., Dündar F., Manke T. (2016). deepTools2: a next generation web server for deep-sequencing data analysis. Nucleic Acids Res..

[bib40] Riveiro A.R., Brickman J.M. (2020). From pluripotency to totipotency: an experimentalist’s guide to cellular potency. Development.

[bib41] Robinson J.T., Thorvaldsdóttir H., Winckler W., Guttman M., Lander E.S., Getz G., Mesirov J.P. (2011). Integrative genomics viewer. Nat. Biotechnol..

[bib42] Skene P.J., Henikoff S. (2017). An efficient targeted nuclease strategy for high-resolution mapping of DNA binding sites. Elife.

[bib43] Tanaka S., Kunath T., Hadjantonakis A.-K., Nagy A., Rossant J. (1998). Promotion of Trophoblast Stem Cell Proliferation by FGF4. Science.

[bib44] Thompson J.J., Lee D.J., Mitra A., Frail S., Dale R.K., Rocha P.P. (2022). Extensive co-binding and rapid redistribution of NANOG and GATA6 during emergence of divergent lineages. Nat. Commun..

[bib45] Virtanen P., Gommers R., Oliphant T.E., Haberland M., Reddy T., Cournapeau D., Burovski E., Peterson P., Weckesser W., Bright J. (2020). SciPy 1.0: fundamental algorithms for scientific computing in Python. Nat. Methods.

[bib46] Waskom M. (2021). seaborn: statistical data visualization. J. Open Source Softw..

[bib47] White M.D., Angiolini J.F., Alvarez Y.D., Kaur G., Zhao Z.W., Mocskos E., Bruno L., Bissiere S., Levi V., Plachta N. (2016). Long-Lived Binding of Sox2 to DNA Predicts Cell Fate in the Four-Cell Mouse Embryo. Cell.

[bib48] Wolf F.A., Angerer P., Theis F.J. (2018). SCANPY: large-scale single-cell gene expression data analysis. Genome Biol..

[bib49] Wong Y.F., Kumar Y., Proks M., Herrera J.A.R., Rothová M.M., Monteiro R.S., Pozzi S., Jennings R.E., Hanley N.A., Bickmore W.A., Brickman J.M. (2023). Expansion of ventral foregut is linked to changes in the enhancer landscape for organ-specific differentiation. Nat. Cell Biol..

[bib50] Yang J., Ryan D.J., Wang W., Tsang J.C.H., Lan G., Masaki H., Gao X., Antunes L., Yu Y., Zhu Z. (2017). Establishment of mouse expanded potential stem cells. Nature.

[bib51] Yang Y., Liu B., Xu J., Wang J., Wu J., Shi C., Xu Y., Dong J., Wang C., Lai W. (2017). Derivation of Pluripotent Stem Cells with In Vivo Embryonic and Extraembryonic Potency. Cell.

[bib52] Ying Q.L., Wray J., Nichols J., Batlle-Morera L., Doble B., Woodgett J., Cohen P., Smith A. (2008). The ground state of embryonic stem cell self-renewal. Nature.

[bib2] Quinlan A.R., Hall I.M. (2010). BEDTools: a flexible suite of utilities for comparing genomic features. Bioinformatics..

