# Supplementary material for: Transcription factor co-expression mediates lineage priming for embryonic and extra-embryonic differentiation
Source: Stem Cell Reports. 2024 Jan 11;19(2):174–86. doi: 10.1016/j.stemcr.2023.12.002 (PMC10874857; doi:10.1016/j.stemcr.2023.12.002)
Supplement: Document S1. Supplemental experimental procedures and Figures S1–S4 [file mmc1.pdf]

Stem Cell Reports, Volume *19*

Supplemental Information

Transcription factor co-expression mediates lineage priming for embryonic and extra-embryonic differentiation

Alba Redó-Riveiro, Jasmina Al-Mousawi, Madeleine Linneberg-Agerholm, Martin Proks, Marta Perera, Nazmus Salehin, and Joshua M. Brickman

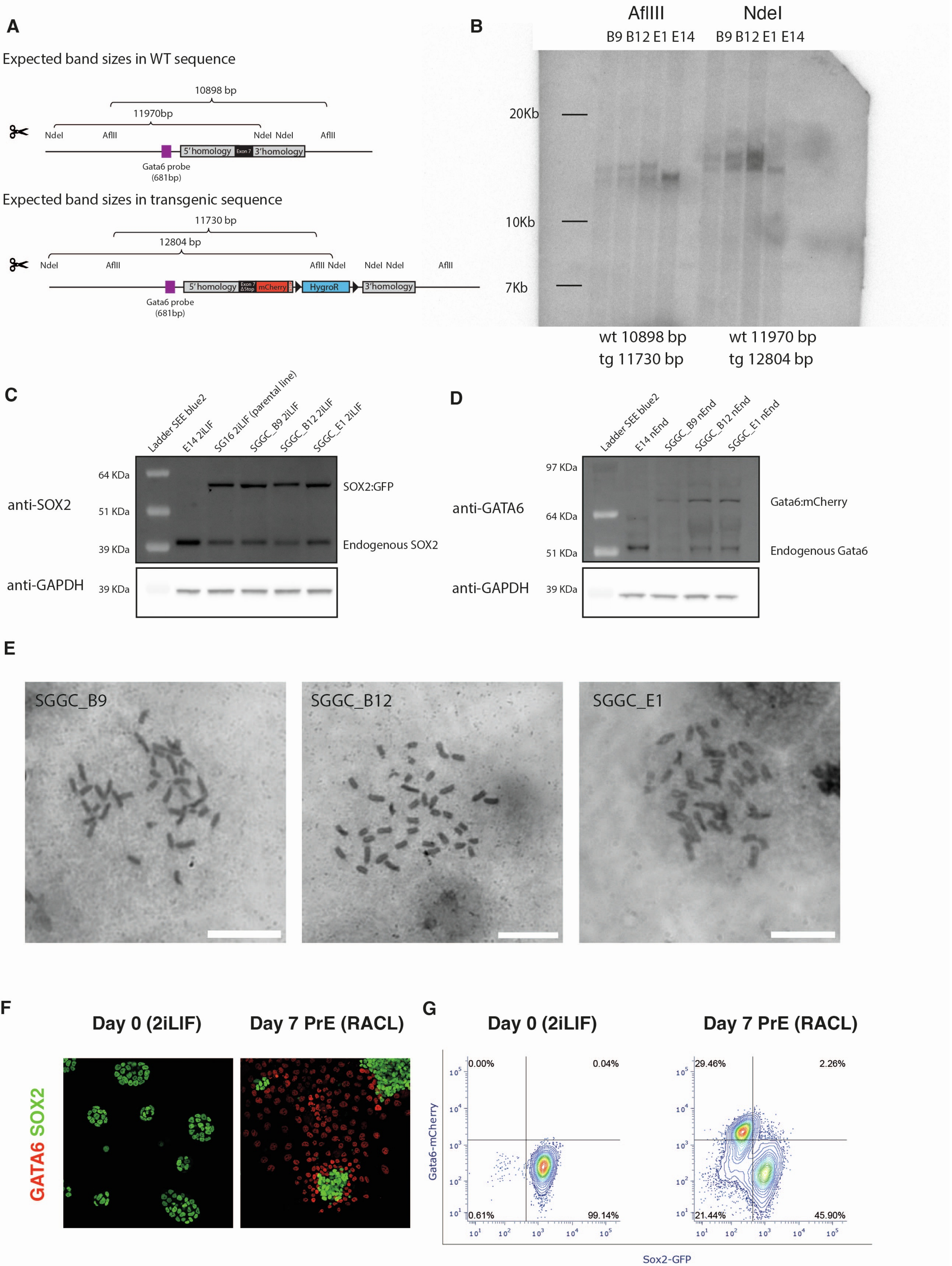
A
Expected band sizes in WT sequence
10898 bp
11970bp
NdeI
AflI
NdeI NdeI
AflII
5'homology
Exon 7
3'homology
Gata6 probe
(681bp)
Expected band sizes in transgenic sequence
11730 bp
12804 bp
NdeI
AflI
AflII NdeI
NdeI NdeI
AflII
5' homology
Exon 7 ΔStop
mCherry
HygroR
3'homology
Gata6 probe
(681bp)
B
AflIII
NdeI
B9 B12 E1 E14
B9 B12 E1 E14
20Kb
10Kb
7Kb
wt 10898 bp
tg 11730 bp
wt 11970 bp
tg 12804 bp
C
Ladder SEE blue2
E14 2iLIF
SG16 2iLIF (parental line)
SGGC_B9 2iLIF
SGGC_B12 2iLIF
SGGC_E1 2iLIF
64 KDa
51 KDa
39 KDa
anti-SOX2
SOX2:GFP
Endogenous SOX2
anti-GAPDH
39 KDa
D
Ladder SEE blue2
E14 nEnd
SGGC_B9 nEnd
SGGC_B12 nEnd
SGGC_E1 nEnd
97 KDa
64 KDa
51 KDa
anti-GATA6
Gata6:mCherry
Endogenous Gata6
anti-GAPDH
39 KDa
E
SGGC_B9
SGGC_B12
SGGC_E1
F
Day 0 (2iLIF)
Day 7 PrE (RACL)
GATA6 SOX2
G
Day 0 (2iLIF)
Day 7 PrE (RACL)
0.00%
0.04%
0.61%
99.14%
29.46%
2.26%
21.44%
45.90%
Gata6-mCherry
Sox2-GFP

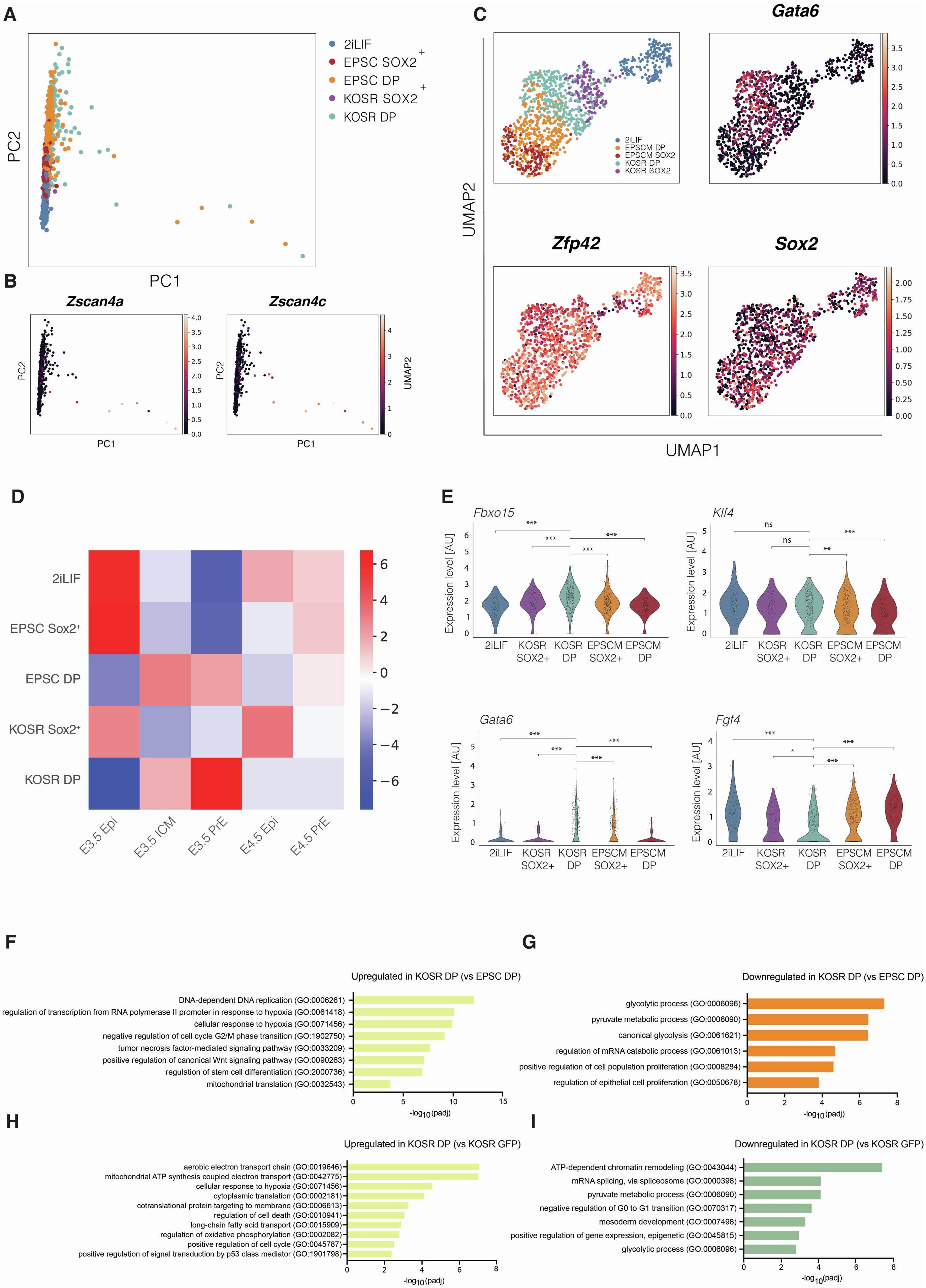
A
2iLIF
EPSC SOX2+
EPSC DP
KOSR SOX2+
KOSR DP
PC2
PC1
B
Zscan4a
Zscan4c
PC2
PC1
C
Gata6
Zfp42
Sox2
UMAP2
UMAP1
2iLIF
EPSCM DP
EPSCM SOX2
KOSR DP
KOSR SOX2
D
2iLIF
EPSC Sox2+
EPSC DP
KOSR Sox2+
KOSR DP
E3.5 Epi
E3.5 ICM
E3.5 PrE
E4.5 Epi
E4.5 PrE
E
Fbxo15
Klf4
Gata6
Fgf4
Expression level [AU]
2iLIF
KOSR SOX2+
KOSR DP
EPSCM SOX2+
EPSCM DP
F
Upregulated in KOSR DP (vs EPSC DP)
DNA-dependent DNA replication (GO:0006261)
regulation of transcription from RNA polymerase II promoter in response to hypoxia (GO:0061418)
cellular response to hypoxia (GO:0071456)
negative regulation of cell cycle G2/M phase transition (GO:1902750)
tumor necrosis factor-mediated signaling pathway (GO:0033209)
positive regulation of canonical Wnt signaling pathway (GO:0090263)
regulation of stem cell differentiation (GO:2000736)
mitochondrial translation (GO:0032543)
-log10(padj)
G
Downregulated in KOSR DP (vs EPSC DP)
glycolytic process (GO:0006096)
pyruvate metabolic process (GO:0006090)
canonical glycolysis (GO:0061621)
regulation of mRNA catabolic process (GO:0061013)
positive regulation of cell population proliferation (GO:0008284)
regulation of epithelial cell proliferation (GO:0050678)
-log10(padj)
H
Upregulated in KOSR DP (vs KOSR GFP)
aerobic electron transport chain (GO:0019646)
mitochondrial ATP synthesis coupled electron transport (GO:0042775)
cellular response to hypoxia (GO:0071456)
cytoplasmic translation (GO:0002181)
cotranslational protein targeting to membrane (GO:0006613)
regulation of cell death (GO:0010941)
long-chain fatty acid transport (GO:0015909)
regulation of oxidative phosphorylation (GO:0002082)
positive regulation of cell cycle (GO:0045787)
positive regulation of signal transduction by p53 class mediator (GO:1901798)
-log10(padj)
I
Downregulated in KOSR DP (vs KOSR GFP)
ATP-dependent chromatin remodeling (GO:0043044)
mRNA splicing, via spliceosome (GO:0000398)
pyruvate metabolic process (GO:0006090)
negative regulation of G0 to G1 transition (GO:0070317)
mesoderm development (GO:0007498)
positive regulation of gene expression, epigenetic (GO:0045815)
glycolytic process (GO:0006096)
-log10(padj)

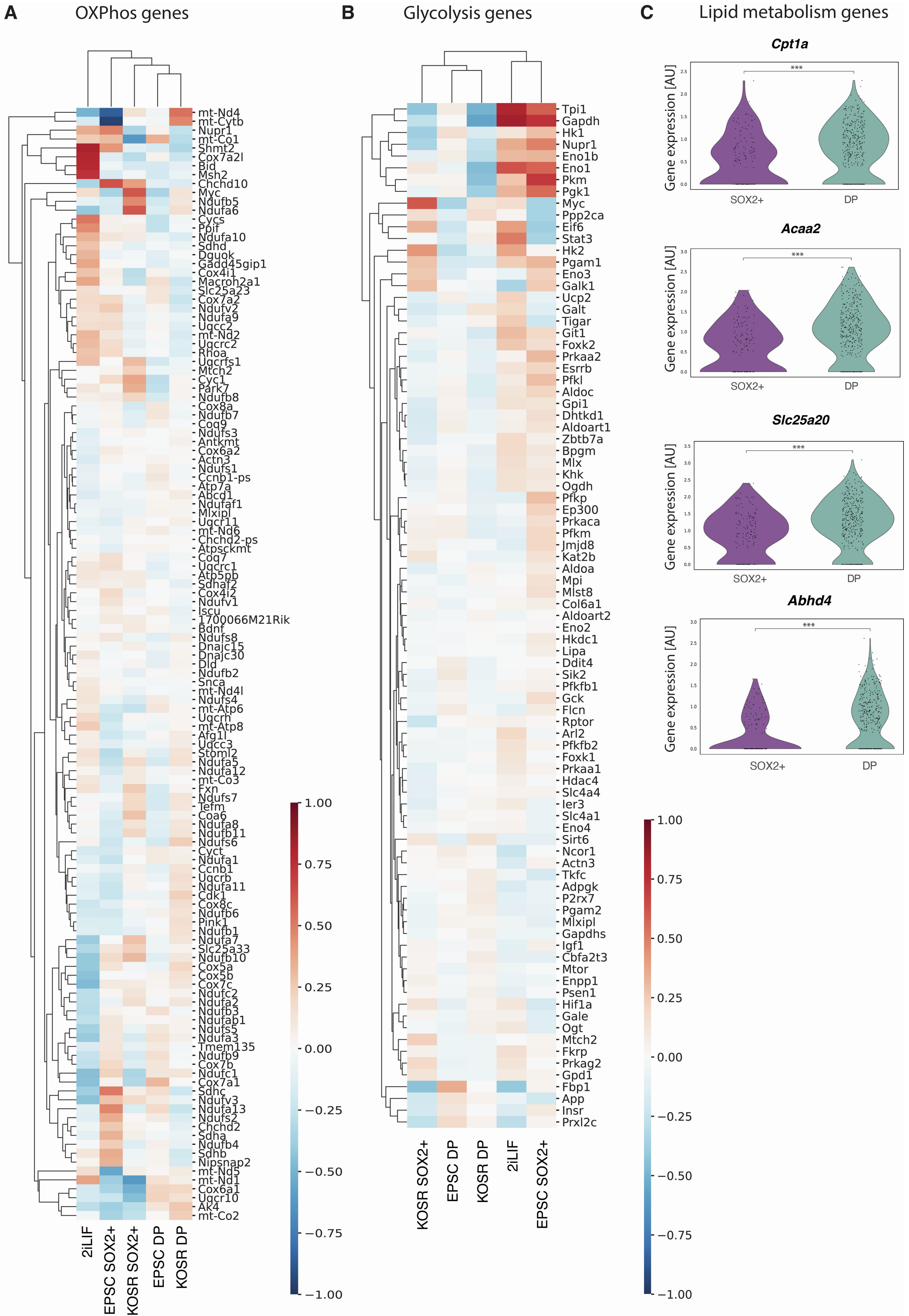
A
OXPhos genes
mt-Nd4
mt-Cytb
Nupr1
mt-Co1
Shmt2
Cox7a2l
Bid
Msh2
Chchd10
Myc
Ndufb5
Ndufa6
Cycs
Ppif
Ndufa10
Sdhd
Dguok
Gadd45gip1
Cox4i1
Macroh2a1
Slc25a23
Cox7a2
Ndufv2
Ndufa9
Uqcc2
mt-Nd2
Uqcrc2
Rhoa
Uqcrfs1
Mtch2
Cyc1
Park7
Ndufb8
Cox8a
Ndufb7
Coq9
Ndufs3
Antkmt
Cox6a2
Actn3
Ndufs1
Ccnb1-ps
Atp7a
Abcd1
Ndufaf1
Mlxipl
Uqcr11
mt-Nd6
Chchd2-ps
Atpsckmt
Coq7
Uqcrc1
Atp5pb
Sdhaf2
Cox4i2
Ndufv1
Iscu
1700066M21Rik
Bdnf
Ndufs8
Dnajc15
Dnajc30
Dld
Ndufb2
Snca
mt-Nd4l
Ndufs4
mt-Atp6
Uqcrh
mt-Atp8
Afg1l
Uqcc3
Stoml2
Ndufa5
Ndufa12
mt-Co3
Fxn
Ndufs7
Tefm
Coa6
Ndufa8
Ndufb11
Ndufs6
Cyct
Ndufa1
Ccnb1
Uqcrb
Ndufa11
Cdk1
Cox8c
Ndufb6
Pink1
Ndufb1
Ndufa7
Slc25a33
Ndufb10
Cox5a
Cox5b
Cox7c
Ndufc2
Ndufa2
Ndufb3
Ndufab1
Ndufs5
Ndufa3
Tmem135
Ndufb9
Cox7b
Ndufc1
Cox7a1
Sdhc
Ndufv3
Ndufa13
Ndufs2
Chchd2
Sdha
Ndufb4
Sdhb
Nipsnap2
mt-Nd5
mt-Nd1
Cox6a1
Uqcr10
Ak4
mt-Co2
2iLIF
EPSC SOX2+
KOSR SOX2+
EPSC DP
KOSR DP
1.00
0.75
0.50
0.25
0.00
−0.25
−0.50
−0.75
−1.00
B
Glycolysis genes
Tpi1
Gapdh
Hk1
Nupr1
Eno1b
Eno1
Pkm
Pgk1
Myc
Ppp2ca
Eif6
Stat3
Hk2
Pgam1
Eno3
Galk1
Ucp2
Galt
Tigar
Git1
Foxk2
Prkaa2
Esrrb
Pfkl
Aldoc
Gpi1
Dhtkd1
Aldoart1
Zbtb7a
Bpgm
Mlx
Khk
Ogdh
Pfkp
Ep300
Prkaca
Pfkm
Jmjd8
Kat2b
Aldoa
Mpi
Mlst8
Col6a1
Aldoart2
Eno2
Hkdc1
Lipa
Ddit4
Sik2
Pfkfb1
Gck
Flcn
Rptor
Arl2
Pfkfb2
Foxk1
Prkaa1
Hdac4
Slc4a4
Ier3
Slc4a1
Eno4
Sirt6
Ncor1
Actn3
Tkfc
Adpgk
P2rx7
Pgam2
Mlxipl
Gapdhs
Igf1
Cbfa2t3
Mtor
Enpp1
Psen1
Hif1a
Gale
Ogt
Mtch2
Fkrp
Prkag2
Gpd1
Fbp1
App
Insr
Prxl2c
KOSR SOX2+
EPSC DP
KOSR DP
2iLIF
EPSC SOX2+
1.00
0.75
0.50
0.25
0.00
−0.25
−0.50
−0.75
−1.00
C
Lipid metabolism genes
Cpt1a
Gene expression [AU]
***
SOX2+
DP
Acaa2
Gene expression [AU]
***
SOX2+
DP
Slc25a20
Gene expression [AU]
***
SOX2+
DP
Abhd4
Gene expression [AU]
***
SOX2+
DP

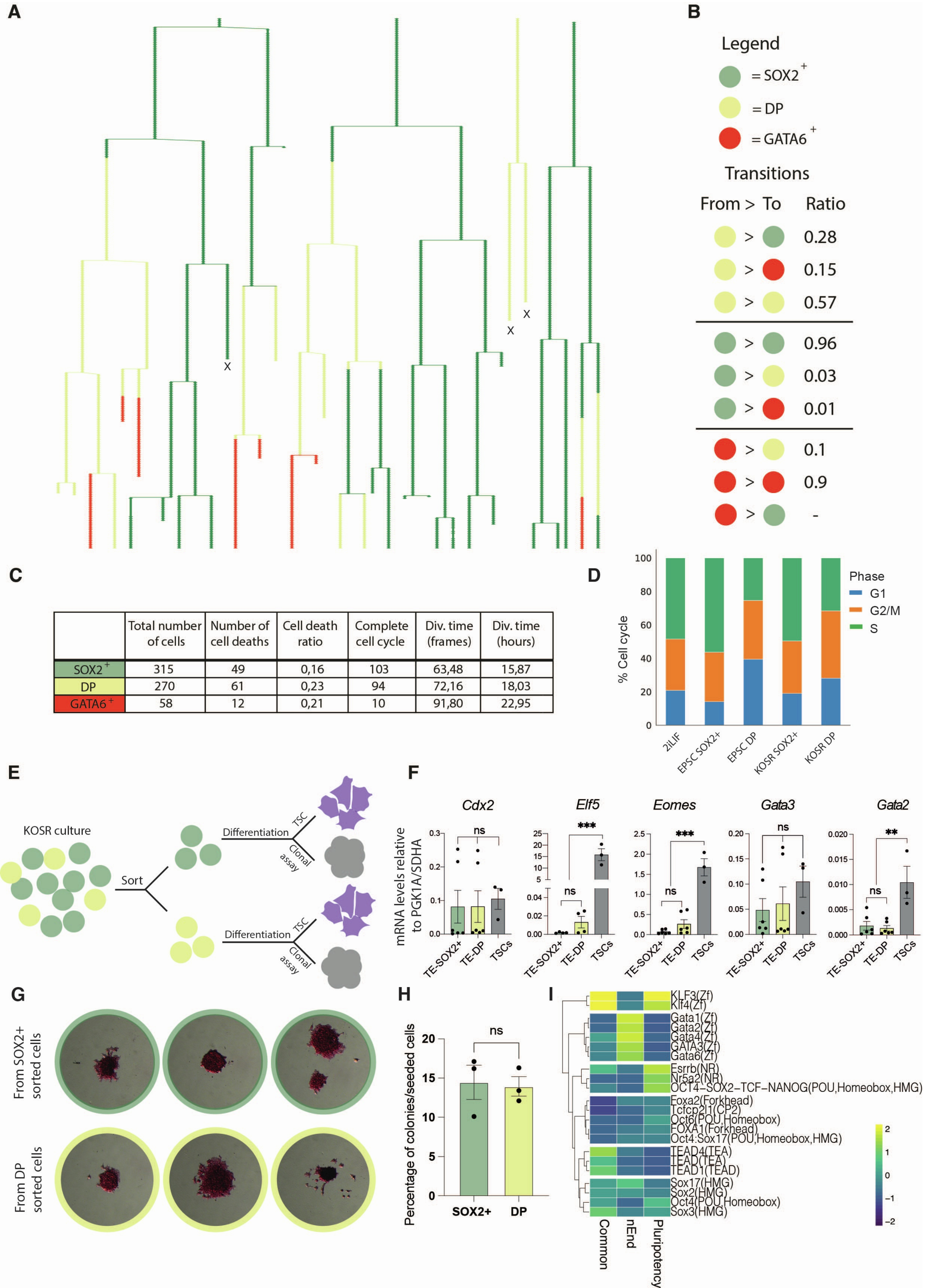

	Total number of cells	Number of cell deaths	Cell death ratio	Complete cell cycle	Div. time (frames)	Div. time (hours)
SOX2+	315	49	0,16	103	63,48	15,87
DP	270	61	0,23	94	72,16	18,03
GATA6+	58	12	0,21	10	91,80	22,95

Supplemental Figure Legends

Fig. S1. Generation of a post-translational double reporter for SOX2 and GATA6. A. Schematic drawing of the WT and transgenic sequences at the *Gata6* locus, including the Gata6 probe used for the Southern blot and the predicted band sizes when cut with enzymes NdeI and AflIII. **B.** Southern blot of the external probe (marked in A) for the Gata6-mCherry construct. 3 correctly targeted SGGC clones (B9, B12 & E1). **C.** Western blot for SOX2 and GAPDH showing two SOX2 bands (endogenous and GFP-tagged) in all the double reporter lines and in the parental line SG16 in 2iLIF conditions. **D.** Western blot for GATA6 and GAPDH showing 2 GATA6 bands (endogenous and mCherry-tagged) in all the double reporter lines in nEnd conditions. **E.** Bright field images of the correct karyotyping of the 3 SGGC clones. Scale bar = 15µm. Modal number for the 3 clones is 40 chromosomes: SGGC B9 at 77% (7/9 spreads) SGGC B12 at 75% (6/6 spreads) and SGGC E1 at 83% (5/6 spreads). **F.** Representative images of the SGGC lines in 2iLIF (naive ESCs) and at day 7 of PrE differentiation (RACL media). **G.** Representative flow cytometry plots of the SGGC cell line in 2iLIF and day 7 of PrE differentiation (RACL media).

Fig. S2. Cell populations identified with the SOX2 and GATA6 double reporter. A. PCA showing the first and second principal components. PC1 separates 2CLCs from the rest. **B.** Expression of 2C genes (*Zscan4c*, *Zscan4d)* projected on PCA plot shown in S2A. **C.** UMAP of single cell RNA-seq of sorted populations (top left) overlain with expression of selected markers (*Gata6, Zfp42* and *Sox2*). **D.** Heatmap showing normalized residuals post-$\chi 2$ test comparing differences in proportions of the sorted cells mapped to *in vivo* scRNA-seq of pre-implantation blastocysts (Nowotschin et. al 2019). Color scale represents the normalized deviation of observed from expected proportions. **E.** Violin plots depicting 4 selected genes from each relevant stage; E3.5 ICM, E3.5 Epi, E3.5 PrE and E4.5 Epi. The plot shows normalized and log-transformed counts. Wilcoxon statistical test used. ns = non-significant * = p-value < 0.05 ** = p-value <0.01 and *** = p-value <0.001. **F-G.** GO term enrichment for KOSR DP cells compared with EPSCM DP cells. **H-I.** GO term enrichment of KOSR DP compared with KOSR SOX2$^+$.

Fig. S3. Metabolic signatures present in DP SOX2 and GATA6 expressing ESCs. A. Heatmap showing oxidative phosphorylation genes expression in the different media conditions. **B**. Heatmap showing glycolysis genes. **C.** Violin plots with different examples of lipid metabolism genes distribution in DP or SOX2$^+$ cells in KOSR media. Mann-Whitney U statistical test. * = p-value 0.01, *** = p-value 0.001

Fig S4. Interconversion and differentiation of SOX2 and GATA6 expressing ESCs. A. Representative dendrogram of 4 initial tracked cells with its descendants (from Video S1). A total of 63 individual tracks of 72h were tracked. The x marks cell death. **B.** Ratios of transitions from one cell state to another. **C.** Table with quantification of the timelapse data. **D.** Distribution of the cell cycle phases in different sorted populations based on scRNA-seq data. For each cell, its cell cycle phase was estimated using CellCycleScoring function from Seurat. The barplot depicts cell cycle phase proportion for different cell stages. **E.** Schematic of TSC differentiation and clonal assay after sorting KOSR cells by FACS. **F.** Relative mRNA levels of TSC markers of the sorted SOX2$^+$ (green bar) or DP (yellow bar) sorted populations after 6 days of TSC differentiation. 2 individual clones were used. Grey bars show control values from Trophoblast stem cells derived from post-implantation embryos. Columns show mean ±

standard error of the mean (SEM). Statistics show unpaired t-tests. ns = non-significant * = p-value < 0.05 ** = p-value <0.01 and *** = p-value <0.001. **G.** Representative images of AP-stained colonies after 1 week of clonal growth from SOX2+ sorted cells (green frame) or DP sorted cells (yellow frame). **H.** Quantification of the AP staining from the clonal assay. **I.** Heatmap of selected motifs across enhancer subsets defined in Figure 3B, with a cutoff of p-value < 0.0005. Scale shows -log10(p-value).

Tables:

Table 1 SOX2 and GATA6 binding. Table shows a list of the closest epiblast genes to the cobound peaks for SOX2 and GATA6 in DP cells. It also shows how many SOX2 and GATA6 motifs we find in these regions. Genes colored in yellow are differentially upregulated in DP over $SOX2^+$ cells in the scRNA-seq. The ones colored in green are differentially upregulated in $SOX2^+$ over DP cells.

Table 2 Characterization of DP SOX2 and GATA6 cobound regions. Table shows a list of the closest PrE genes to the cobound peaks for SOX2 and GATA6 in DP cells. It also shows how many SOX2 and GATA6 motifs we find in these regions. Genes colored in yellow are differentially upregulated in DP over $SOX2^+$ cells in the scRNA-seq. The ones colored in green are differentially upregulated in $SOX2^+$ over DP cells.

Table 3 Comparison of this study to Thompson *et al. 2022*. Table compares SOX2 and GATA6 binding peaks from our study with the SOX2 and GATA6 peaks of the GATA6 overexpression time course from Thompson *et al.* 2022.

Supplemental experimental procedures

Generation of SGGC ESC lines

SOX2:GFP ESCs were plated onto a gelatinised 6-well plates in serum/LIF and lipofected with the linearized Gata6:mCherry-Hygromycin plasmid and the CRISPR plasmid using Lipofectamine2000 (ThermoFisher) following the manufacturer's instructions. After 16 h incubation, the cells were transferred onto 10cm dishes with fresh medium supplemented with Hygromycin to select for colonies successfully integrated. SOX2:GFP ESCs (Anderson et al., 2017) (specifically clone SG16) was used to further target with a Gata6:mCherry construct using CRISPR-Cas technology. The construct contains mCherry tagged immediately after exon 7 of the Gata6 locus, just before the STOP codon and the construct has 3000bp homology arms. We obtained 3 clones (B9, B12 and E1) that were successfully integrated. Clone B9 was generated using CRISPR guide 1 (GCTCTGGCCCTGGCC), which cuts at the last 20nt of the coding sequence of Gata6, and clones B12 and E1 were generated using CRISPR guide 2 (GCACAGAAATCACGCATCGA), which cuts 150 bp after the STOP codon (See Fig. 1A).
SGGC cells were verified by performing immunostaining for SOX2, GATA6, mCherry and GFP, western blot, locus sequencing to screen for unwanted mutations generated by CRISPR, karyotyping and Southern blot. Resistance cassettes can be easily removed using Cre-mediated recombination, however we decided to use the original clones with the resistance cassettes included. All 3 clones give the same reproducible results. All 3 clones were used for the MARSeq scRNA-seq. Clones B9 and B12 presented the highest amount of double positive cells, so these 2 clones were used in all the CUT&RUN, differentiations and morula aggregation experiments.

Karyotyping
SGGC ESCs were expanded until 50-60% confluency, after which they were incubated for 1h in medium containing 10 µg/ml Colcemid (Sigma Aldrich). The medium was collected for separate disposal, and without a PBS washing step, 2 ml of trypsin were added. When the first cells started to detach, the trypsin was inactivated by adding medium. Pelleted cells were resuspended in 2.5 ml hypotonic solution (0.56 % (w/v) KCl and incubated at RT for exactly 6 min. 1 ml of fixative (75 % (v/v) methanol, 25 % (v/v) acetic acid) was added, followed by 1 wash in 1 ml of fixative and 30 min incubation at 4°C. After 2 washes in fixative, cells were carefully resuspended in 200µl fixative and spread onto pre-cleaned poly-L-lysine coated glass slides. Chromosomes were stained for 30 min in filtered 10% Giemsa pH 7.2 solution and imaged using a 63x Oil objective on an inverted Olympus microscope.

Southern blot
Southern Blotting was used to test SGGC clones for correct gene targeting as previously described (Southern, 2006). Gata6:mCherry construct integration was confirmed using both an internal (mCherry) and an external probe. DNA for the external probe was cut using AflIII and NdeI. The DNA for mCherry internal probe was cut using HindIII (Fig. S1A-B). Hybridization of the probe happened at 60°C O/N. The blots were left in the exposure cassette for 48h and developed using a high-resolution Typhoon scanner.

Flow cytometry and FACS
Cells were washed in PBS and brought to single cell suspension using Accutase. Cells were resuspended in 10% FCS/PBS with DAPI (1:10,000) to exclude dead cells. Cells were analyzed using a LSR Fortessa flow cytometer (BD Biosciences) and the FACSDiva (BD Biosciences) software. Cells were sorted by SOX2^{+} or DP by FACS using a BD FACS Aria III (BD Biosciences) with a 100µm nozzle and 20psi sheath pressure. The gate between positive and negative populations were set based on the negative population of control ESCs (both E14 and parental line SG16). A forward scatter (FSC) and side scatter (SSC) was used to define a homogeneous population, FSC-H/FSC-W gates were used to exclude doublets, and dead cells were excluded based on DAPI staining. Plots were generated using FCS Express 6.0 (DeNovo Software).

Alkaline Phosphatase Staining
ESCs were plated at clonal density and cultured for 7 days. Alkaline phosphatase staining was carried out as per manufacturer's instructions. Colonies were imaged using a Leica M165 C microscope.

ESC and embryo immunostaining
ESCs were cultured in 8-wells slides (Ibidi) and immunostaining was carried out as previously described (Canham et al., 2010). Primary antibodies are listed in the resource table. Secondary antibodies used are from the Alexa Fluor series (Molecular Probes, ThermoFisher), all 1:2000. Blastocysts were stained by anti-CDX2 (BioGenex MU392A-UC) used at 1:200. Both for blastocyst and E6.5 embryos anti-GATA6 (Cell Signalling Technologies, 5851) was used at 1:200. Both mESCs and embryos were imaged using a confocal Leica SP8.

Quantitative PCR with reverse transcription (RT-qPCR)
Total RNA was collected using the RNeasy Mini Kit (Qiagen). 1µg of total RNA was used for first strand synthesis using SuperScript III reverse transcriptase according to the manufacturer's

instructions. cDNA corresponding to 10ng total RNA was used for RT–qPCR analysis using the Roche LC480 and target amplification was detected with the Universal Probe Library system.

scRNA-seq

Cells were sorted directly into 384-well plates containing lysis buffer which includes the first RT primer and RNase inhibitor, immediately frozen and later processed by the MARS-seq2 protocol (Keren-Shaul et al., 2019). scRNA-seq libraries were sequenced using an Illumina NextSeq500 at a median sequencing depth of 225,000 reads per single cell. Pre-processing was done using nfcore/marsseq pipeline (Proks et al., 2023) with following command: nextflow run nf-core/marsseq -r 1.0.3 -profile ku_sund_dangpu –with-tower --genome mm10 --velocity --input SCR_20221006/raw/samplesheet.csv --outdir /scratch/ALBA_SB2/ .

Time-lapse imaging and cell tracking

SGGCH2B ESC lines were cultured in KOSR media, on 8-well slides (ibidi) and imaged every 15 minutes across 72h. mCherry, GFP and CY5 fluorescent light channels were recorded in 5% CO_2, 20% O_2 at 37°C under a Deltavision Widefield Screening microscope. ESCs were seeded at 5000 cells/cm^2 24h before the beginning of the time lapse in KOSR. We performed manual cell tracking using Imaris v9.5 (Bitplane). Nuclei were segmented using the H2B marker tagged with far red. We measured the SOX2-GFP and GATA6-mCherry fluorescence intensities of a circular area of 50 µm diameter inside the segmented nuclei. For each area measured, we took the median fluorescence intensity as the measure for that given data point. Intensity measurements were linked to its time point and lineage, allowing us to infer the division time for each cell that was tracked, as well as the expression level of both SOX2 and GATA6 in each time point. Only cells with completed cell cycle information were used for calculating the transition analysis. A total of 63 individual tracks of 72h have been tracked.

CUT&RUN

KOSR, nEnd, 2iLIF or NACL cells were grown in their respective media for at least 4 passages. KOSR and nEnd cells were sorted by FACS using a BD FACS Aria III. From KOSR we sorted $SOX2^+$ and DP cells. From nEnd we sorted $GATA6^+$ cells. A minimum of 100,000 cells were sorted to proceed with the CUT&RUN protocol. CUT&RUN was performed using an in house purified MNase and following the published protocol (Janssens and Henikoff, 2019). Library preparation was performed following this published protocol (Liu, 2019). The quality of the CUT&RUN libraries was assessed using an Agilent Fragment Analyzer and quantified using Qubit. Sequencing was performed on an Illumina NextSeq 550, up to 48 uniquely barcoded samples were pooled on a High Output flow cell. Samples are sequenced using paired end sequencing. Antibodies used: Sox2-Active Motif 39843, Gata6-Cell Signaling 5851, H3K27ac Abcam-ab4729, H3K4me1 Abcam-ab8895. All used at 1:100 concentration.

Downstream analysis of CUT&RUN

Reads were trimmed to 21bp with Cutadapt (Martin, 2011), mapped to the GRCm38/mm10 mouse reference genome with Bowtie 2 (Langmead and Salzberg, 2012), with options -X 1000 –no- mixed –no-discordant. Only uniquely mapped reads with MAPKQ>10 were kept using SAMtools v1.9 (Li et al., 2009). Bam files were sorted, deduplicated with Picard v2.9.1 and indexed. Bam files for TFs were subset to contain a maximum fragment length of 150 bp, whereas for histone modifications, all reads were considered. Bedgraphs were generated using bedtools v2.27.1. (Ar and Im, 2010). Bigwig files were generated with bamCoverage

v3.2.0 from DeepTools (Ramírez et al., 2016) and the RPGC option (-bs=1 –normalizeUsing RPKM). Peaks were called using SEACR (Meers et al., 2019), parameters; ’relaxed’ for transcription factors and ‘Stringent’ for histone marks. Peaks were called against a negative IgG control, generated in each experiment for each condition. GREAT was used to annotate peaks to nearest genes and perform GO enrichment (McLean et al., 2010). Motif analysis was performed on select genomic regions using HOMER findMotifsGenome.pl with options -size 75 -mask (Heinz et al., 2010). Genome browser tracks were visualized using Gviz/Fluff (Hahne and Ivanek, 2016). Global TF occupancy across enhancer subsets was plotted by first calculating the coverage at each enhancer summit +/- 1 kB using Deeptools computeMatrix (Ramírez et al., 20) and visualized in R using custom scaling that equalized TF signal across enhancer subsets to assesses the relative TF distribution on enhancers within each condition.

Chimera assays

For chimera assays, H2B:miRFP670 tagged clones SGGCH2B B9.A and B12.B were used. Cells were sorted for $SOX2^+$ or DP as previously described. Using an aggregation needle (Type DN-09, BLS Ltd., Hungary), 10 to 12 wells were made in 35mm tissue culture Petri dishes. The wells were covered with microdrops of KOSM and mineral oil. The plate was left in the incubator for 1h at 37°C and 5% CO2 to buffer the medium to the appropriate pH. Live morulae (E2.5) were flushed from the uterus of superovulated C57BL/6NRj female mice by the transgenics platform facility and cultured in KSOM.

DP or $SOX2^+$ sorted cells were placed at the bottom of the wells and 1 morula was added on top.

Embryos were either cultured for 3 days *in vitro* to the equivalent of E4.5 *in vivo* or transferred to pseudopregnant CD1 females (RjOrl:SWISS) (12 weeks old) for further development, and embryos were harvested at E6.5. Animal work was carried in accordance with European legislation. All work was authorized by and carried out under Project License 2018-15-0201-01520 and 2023-15-0201-01513 issued by the Danish Regulatory Authority.

qPCR primers

Gata6F	GGTCTCTACAGCAAGATGAATGG
Gata6R	TGGCACAGGACAGTCCAAG
Gata4 F	TTCGCTGTTTCTCCCTCAAG
Gata4 R	CAATGTTAACGGGTTGTGGA
Gata3 F	TTATCAAGCCCAAGCGAAG
Gata3 R	TGGTGGTGGTCTGACAGTTC
Dab2 F	TCTCAGCCTGCATCTTCTGA
Dab2 R	TTTGCTCATCTGGATAGTCATCAT
Nanog F	cctccagcagatgcaagaa
Nannog R	gcttgcacttcatcctttgg
Oct4 F	GTTGGAGAAGGTGGAACCAA
Oct4 R	CTCCTTCTGCAGGGCTTTC
Oct6 F	catttttcgtttcgttttaccc
Oct6 R	gagcgcagaccctctctg
Otx2 F	ggcctcactttgttctgacc
Otx2 R	aaatcaacttgccagaatcca
Pdgfra F	AAGACCTGGGCAAGAGGAAC
Pdgfra R	GAACCTGTCTCGATGGCACT
Sox7 F	gcggagctcagcaagatg
Sox7 R	gggtctcttctgggacagtg
Sox17 F	CACAACGCAGAGCTAAGCAA
Sox17 R	CGCTTCTCTGCCAAGGTC
Cdx2 F	CACCATCAGGAGGAAAAGTGA
Cdx2 R	CTGCGGTTCTGAAACCAAAT
Elf5 F	GCAGCTCTGCAGCATGAC
Elf5 R	TTCAGCATCATTGAAAAAGGAG
Eomes F	AAGCTCAAGAAAGGAAACATGC
Eomes R	ACCGGCACCAAACTGAGA
Gata2 F	CTAGGAGTTTGGCTGAAAGGG
Gata2 R	CTCTGCCCAGATTGCCACTG

Antibodies

ANTIBODY	BRAND	# ID	CONCENTRATION	USE
SOX2	Active motif	39843	1:1000	CUT&RUN
GATA6	Cell Signaling	5851	1:1000	CUT&RUN, IF
H3K27ac	Abcam	ab4729	1:1000	CUT&RUN
H3K4me1	Abcam	ab8895	1:1000	CUT&RUN
SOX2	Santa Cruz	sc17320	1:200	IF
CDX2	BioGenex	MU-392A-UC	1:200	IF
