# Supplementary material for: Transcription factor co-expression mediates lineage priming for embryonic and extra-embryonic differentiation
Source: Stem Cell Reports. 2024 Jan 11;19(2):174–86. doi: 10.1016/j.stemcr.2023.12.002 (PMC10874857; doi:10.1016/j.stemcr.2023.12.002)
Supplement: Document S2. Article plus supplemental information [file mmc6.pdf]

Stem Cell Reports
Report
OPEN ACCESS

Transcription factor co-expression mediates lineage priming for embryonic and extra-embryonic differentiation

Alba Redó-Riveiro,[1] Jasmina Al-Mousawi,[1,2] Madeleine Linneberg-Agerholm,[1] Martin Proks,[1] Marta Perera,[1] Nazmus Salehin,[1] and Joshua M. Brickman[1,*]

[1]reNEW UCPH – The Novo Nordisk Foundation Center for Stem Cell Medicine, Faculty of Health and Medical Sciences, University of Copenhagen, Blegdamsvej 3B, 2200 Copenhagen N, Denmark

[2]Present address: Epigenetics and Neurobiology Unit, European Molecular Biology Laboratory (EMBL), Via Ramarini 32, Monterotondo 00015, Italy

*Correspondence: joshua.brickman@sund.ku.dk

https://doi.org/10.1016/j.stemcr.2023.12.002

SUMMARY

In early mammalian development, cleavage stage blastomeres and inner cell mass (ICM) cells co-express embryonic and extra-embryonic transcriptional determinants. Using a protein-based double reporter we identify an embryonic stem cell (ESC) population that co-expresses the extra-embryonic factor GATA6 alongside the embryonic factor SOX2. Based on single cell transcriptomics, we find this population resembles the unsegregated ICM, exhibiting enhanced differentiation potential for endoderm while maintaining epiblast competence. To relate transcription factor binding in these cells to future fate, we describe a complete enhancer set in both ESCs and naive extra-embryonic endoderm stem cells and assess SOX2 and GATA6 binding at these elements in the ICM-like ESC sub-population. Both factors support cooperative recognition in these lineages, with GATA6 bound alongside SOX2 on a fraction of pluripotency enhancers and SOX2 alongside GATA6 more extensively on endoderm enhancers, suggesting that cooperative binding between these antagonistic factors both supports self-renewal and prepares progenitor cells for later differentiation.

INTRODUCTION

How do progenitor cells sit at the cusp of two lineages, remaining stable as cell types, but simultaneously prepared for differentiation toward multiple fates? In early mammalian embryos, the progenitors of the embryonic epiblast and extra-embryonic primitive endoderm (PrE) stably express antagonistic transcription factors (TFs) that will eventually drive epiblast and PrE lineage specification. Instead of undergoing spontaneous differentiation to both lineages, these cells exist stably across several cell cycles *in vivo* (Dietrich and Hiiragi, 2007). Here, we ask the question of how these cells express antagonistic factors and what function this might have in development and differentiation, focusing on how endoderm and epiblast enhancers become primed in different *in vitro* conditions.

Naive embryonic stem cells (ESCs) are pluripotent cells derived from the inner cell mass (ICM) of the mammalian blastocyst, able to both self-renew and generate all the lineages of the future embryo, but not the extra-embryonic lineages (Morgani et al., 2017; Nichols and Smith, 2011). ESCs can be cultured in a range of conditions, including defined media that support slightly different sub-populations along the spectrum of lineage specification. Culture in serum with the cytokine leukemia inhibitory factor (LIF) or defined basal media supplemented with Activin A, a GSK3 inhibitor (CHIR99021) and LIF (NACL) (Anderson et al., 2017) produce cells primed toward both an epiblast and endoderm identity. By contrast, cells cultured with a MEK inhibitor (PD0325901), CHIR99021 and LIF (2iLIF) (Ying et al., 2008) homogeneously express markers related to epiblast identity and contain a small sub-population that co-express both epiblast protein and extra-embryonic RNA (Morgani et al., 2013). This isolated subpopulation is experimentally totipotent (Riveiro and Brickman, 2020). Another subpopulation shown to exhibit experimental totipotency are 2-cell-like cells, a rare subpopulation that arises spontaneously in ESC culture and expresses factors from the 2-cell (2C) stage embryo (Genet and Torres-Padilla, 2020).

In this paper, we explore how the co-expression of epiblast and PrE factors influence differentiation and TF occupancy. We identify spontaneously arising ESCs that co-express SOX2 and GATA6, where these two factors govern an early ICM-like state. Single cell RNA sequencing (scRNA-seq) revealed that these cells, grown in KnockOut Serum Replacement media (KOSR), are poised for both embryonic and extra-embryonic differentiation. Based on multiple enhancer sets generated from ESC and PrE or naive extra-embryonic endoderm (nEnd) states *in vitro*, we found that SOX2 is recruited to a subset of PrE enhancers and, to a lesser extent, GATA6 to pluripotency enhancers. These findings suggest that the cooperative binding of SOX2 and GATA6 primes enhancer states, potentially setting up competence for differentiation.

RESULTS

KOSR promotes early ICM-like cells in culture

While we had previously described the co-expression of epiblast/ICM TFs with extra-embryonic RNA (Morgani

This is an open access article under the CC BY license (http://creativecommons.org/licenses/by/4.0/).

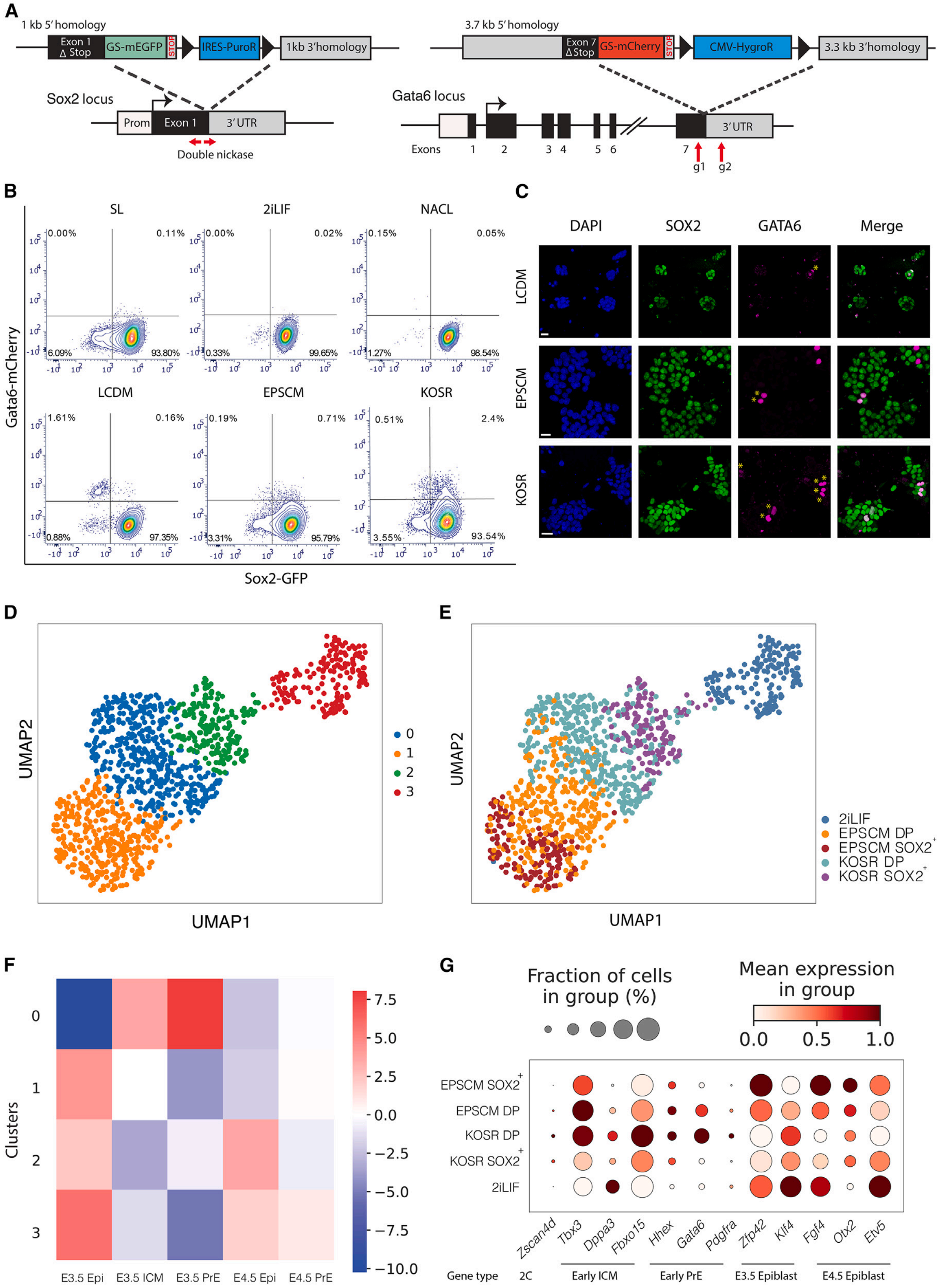

(legend on next page)

et al., 2013), the co-expression of lineage opposing TFs in ESC culture is rare. To identify conditions that support a co-expressing sub-population, we generated a protein-based double reporter SOX2-GFP/GATA6-mCherry (SGGC) ESC line, with the endogenous epiblast and pluripotency factor SOX2 fused to GFP and the PrE TF GATA6 fused to mCherry (Figures 1A and S1A–S1E). We confirmed that SOX2-GFP was expressed when SGGC ESCs were cultured under naive conditions in 2iLIF and that GATA6-mCherry was expressed following differentiation toward PrE (Anderson et al., 2017) (Figures S1F–S1G). We then explored whether a GATA6/SOX2 double-positive (DP) population could be trapped in different culture conditions, including 2iLIF, NACL, and KOSR (Anderson et al., 2017; Garcia-Gonzalo and Izpisúa Belmonte, 2008; Ying et al., 2008). We also tested two culture conditions reported to produce ESCs with enhanced or expanded potential stem cell media (EPSCM) (Yang et al., 2017a), and extended pluripotent stem cell media containing LIF, CHIR99021, DiM, and MiH (LCDM) (Yang et al., 2017b). Cells cultured in EPSCM and KOSR contained a modest fraction of DP cells (1%–5%), while none of the other culture conditions significantly supported this population (Figure 1B). We confirmed these findings by immunostaining (Figure 1C), indicating that both EPSCM and KOSR cultures can support a small stable DP subpopulation.

To determine the nature of DP cells in EPSCM and KOSR and what their equivalent *in vivo* cell state might be, we performed scRNA-seq of SGGC cells in KOSR and EPSCM using MARS-seq2 (Keren-Shaul et al., 2019). Cells were cultured in KOSR, EPSCM or 2iLIF for at least 4 passages. DP and SOX2-GFP single positive (SOX2$^+$) cells were isolated by fluorescence-activated cell sorting (FACS) alongside 2iLIF control cells. After pre-processing and quality filtering, our dataset comprised 1,139 cells and 21,590 genes. Using principal component analysis (PCA), the separation of the datasets for PC1 is driven by positive expression of 2C genes (*Zscan4*, *Dux*, and *Tcstv3*), while PC2 is driven by all three culture conditions (Figures S2A and S2B).

To assess the identity of these populations, we visualized the data using Uniform Manifold Approximation and Projection (UMAP) and used unsupervised clustering to define a total of four clusters (Figure 1D). KOSR and EPSCM SOX2$^+$ cells cluster independently from each other, with the KOSR SOX2$^+$ cells found uniquely in cluster 2, sitting between 2iLIF (cluster 3) and the KOSR DP cells (Figure 1E). KOSR DP cells are all found within a single cluster, cluster 0, whereas ESPSC DP cells are split between clusters 1 and 0 (Figure 1E). Cluster 0 also contains the majority of GATA6-positive cells, a significant proportion of which express *Sox2* mRNA. Pluripotency markers such as *Zfp42* are expressed throughout the four clusters (Figure S2C). To establish the identity of cells and clusters derived in specific culture conditions, we integrated our data with *in vivo* data from the blastocyst (Nowotschin et al., 2019) and found that only cluster 0 resembled the ICM and early PrE (Figure 1F), and, while there are few cells in this cluster from EPSC, 84% of the cells are KOSR DP. While both the EPSCM DP and KOSR DP exhibit some ICM marker expression (Figure S2D), we observed significant upregulation of both early ICM (*Tbx3*, *Dppa3*, and *Fbxo15*) and endoderm (*Gata6*, *Pdgfra*, and *Hhex*) genes specifically in KOSR DP cells compared with EPSCM DP cells (Figures 1G and S2E).

Gene Ontology (GO) analysis of differentially expressed genes between KOSR DP and EPSC DP revealed an enrichment of terms related to metabolism. KOSR DP and EPSCM DP cell transcriptomes were enriched for processes such as hypoxia and mitochondrial activity and pathways related to glycolysis and pyruvate metabolism, respectively (Figures S2F and S2G). In comparison with KOSR SOX2$^+$, KOSR DP cells were enriched for genes related to oxidative phosphorylation (e.g., *Cox5a* and *Cox6c*) and lipid metabolism (e.g., *Cpt1a* and *Slc25a20*), as well as regulation of cell death processes and p53 activity (Figures S2H, S2I, and S3A–S3C). Oxidative phosphorylation and lipid metabolism are characteristic of the pre-implantation ICM, which utilizes these energy sources before shifting to a glycolytic metabolism in the later epiblast, peaking at implantation (E4.5–E5.0) (Leese, 2012).

Taken together, these analyses suggest that ESC culture in KOSR best traps a DP SOX2-GATA6 co-expressing population with ICM-like characteristics, and that, of the tested

Figure 1. GATA6 and SOX2 expression report on an ICM-like sub-population in certain culture states for naive pluripotency
(A) Schematic drawing of the SGGC double reporter.
(B) Flow cytometry contour plots of the SGGC cell lines in the stated media conditions. SL, serum/LIF.
(C) Immunofluorescence images of SOX2 and GATA6 in the stated conditions. Scale bar, 30 μm, yellow stars indicate co-expression.
(D) UMAP of scRNA-seq dataset where coloring represents Louvain clustering.
(E) UMAP of scRNA-seq dataset showing the different cell populations sequenced.
(F) Heatmap showing normalized residuals post-χ^2 test comparing differences in proportions of the Seurat clusters mapped to *in vivo* scRNA-seq of pre-implantation blastocysts (Nowotschin et al., 2019). Color scale represents the normalized deviation of observed from expected proportions.
(G) Dot plot graph showing the log normalized average expression of selected genes across different media conditions. The average expression is marked by the color scale and the percentage of expression by the size of the circle.

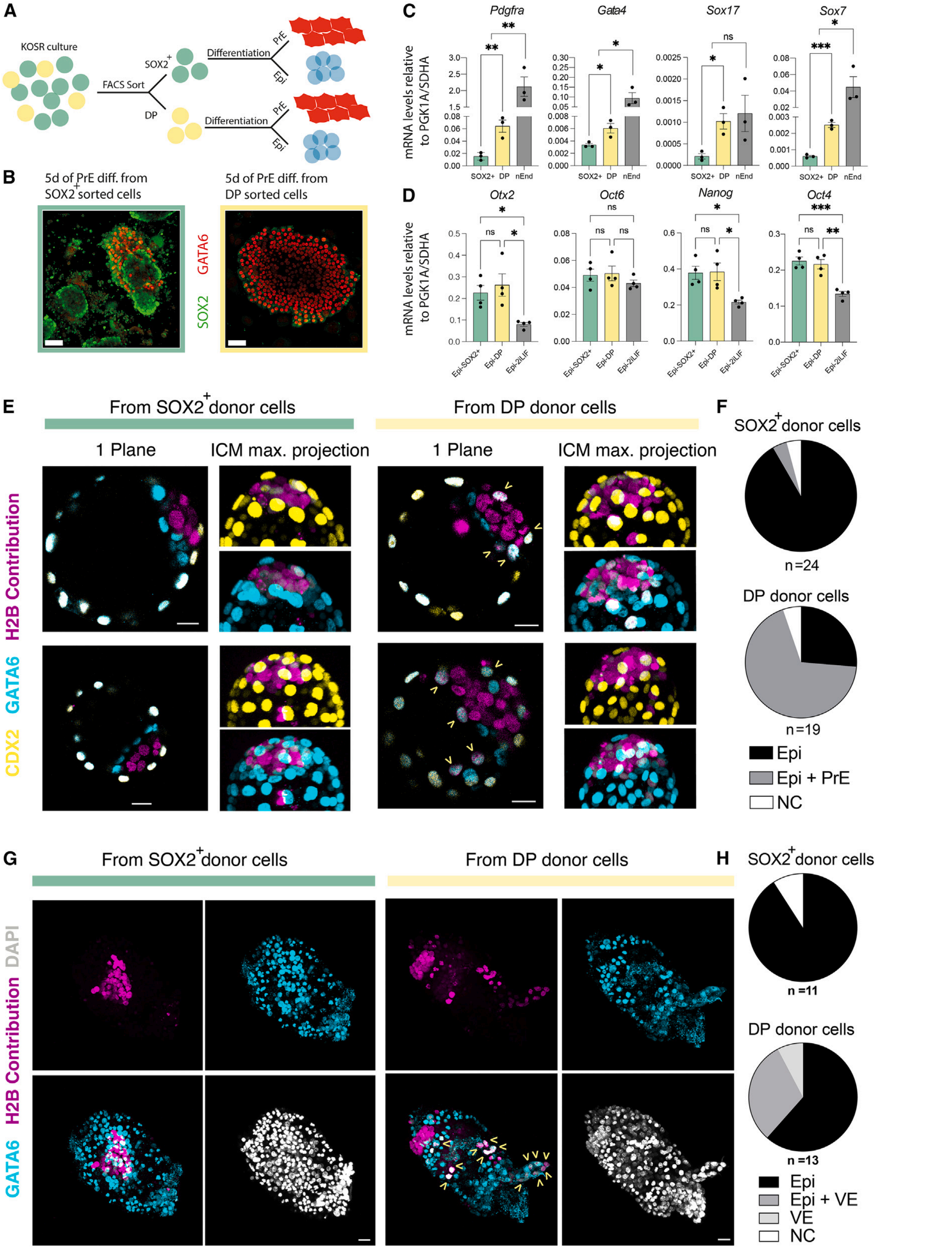

(legend on next page)

conditions, KOSR is the best candidate for exploring the molecular events that underlie endoderm and epiblast priming *in vivo*.

KOSR DP cells are dynamic and primed for PrE differentiation

To study the behavior of the DP population in KOSR, we performed live imaging of steady-state KOSR culture for 72 h, a time period sufficient to enable DP cells to arise and revert to single $SOX2^+$ or $GATA6^+$ cells. Based on lineage tracking of individual cells and their descendants, we found that when DP cells arise, they maintain expression of both GATA6-mCherry and SOX2-GFP for around two cell cycles (~36 h) (Figure S4A). DP cells divide into distinct daughter cells, with 57% of them maintaining their phenotype, 28% converting to $SOX2^+$, and 15% into $GATA6^+$ (Figure S4B). The division rate of $SOX2^+$ cells is fastest and the $GATA6^+$ cells is slowest, with the DP population intermediate between the two, suggesting that these media conditions are optimal for epiblast expansion. Consistent with this, the relative number of cells undergoing cell death is slightly lower in the $SOX2^+$ cells (23% in DP vs. 16% in $SOX2^+$) (Figure S4C, Video S1).

Since DP cells (both EPSCM and KOSR DP cells) have an overall longer cell cycle, we determined the distribution of cell cycle stages in the different populations in the scRNA-seq dataset. We observed that the DP populations of both EPSCM and KOSR have more cells in the G1 and G2 phases compared with the control 2iLIF cells and the $SOX2^+$ sister cells (Figure S4D). As cells primed for PrE differentiation have been shown to stay for longer in G1 (Coronado et al., 2013; Perera et al., 2022), this could explain why DP cells possess a longer G1 phase. An increase in the G2 phase may be linked to an increase in cell death in the DP cells, as G2 arrest normally precedes apoptosis (Pietenpol and Stewart, 2002).

Given the dynamic nature of the DP population, we reasoned that spontaneously occurring ICM-like cells could represent an intermediate in PrE differentiation that is also capable of giving rise to epiblast. Therefore, ICM-like DP cells should exhibit an enhanced capacity or bias to undergo PrE differentiation, relative to single $SOX2^+$ ESCs, but this should not be at the expense of a decrease in the efficiency with which they differentiate to epiblast. To test this hypothesis, we assessed the relative efficiency of KOSR DP and $SOX2^+$ cells to differentiate into lineages of the preimplantation embryo (Figures 2A and S4E). When directed toward PrE (Anderson et al., 2017), sorted DP cells rapidly produce robust $SOX2^-/GATA6^+$ monolayers within 5 days, whereas $SOX2^+$ cells only partially differentiate (Figures 2B and 2C). In contrast, the epiblast (Figure 2D) or trophectoderm (Figures S4E and S4F) differentiation efficiency of these two populations is not significantly different. Finally, we also found that individual $SOX2^+$ and DP cells have a similar capacity to support the expansion of undifferentiated colonies in clonal assays (Figures S4E, S4G, and S4H). Since DP cells can readily differentiate to Epi-like and PrE, mimicking ICM identity, we performed morula aggregation with H2B-miRFP670 tagged $SOX2^+$- and DP-sorted cells and analyzed their contribution to host blastocysts. As expected, $SOX2^+$ cells extensively contributed solely to the epiblast (22/24

Figure 2. GATA6 and SOX2 DP ESCs have ICM-like bipotency for extra-embryonic endoderm and epiblast differentiation

(A) Representative drawing of the PrE and Epi-like differentiations performed after sorting KOSR cells into $SOX2^+$ and DP cells by FACS.

(B) Representative images of the sorted $SOX2^+$ (green frame) and DP (yellow frame) after 5 days (5d) in PrE differentiation media (PrE diff.). Scale bar, 50 μm.

(C) Relative mRNA levels of different PrE genes after 5 days of differentiation from sorted $SOX2^+$ (green bar) and from DP cells (yellow bar). nEnd control values in gray bar. Dots represent three individual differentiations from different individual clones. Columns show mean ± SEM. Statistics show unpaired t tests. Ns, non-significant. $^*p < 0.05$; $^{**}p < 0.01$; $^{***}p < 0.001$.

(D) Relative mRNA levels of different Epi genes after 3 days of Epi-like differentiation from sorted $SOX2^+$ or DP cells. Dots represent four individual differentiations using two independent clones. Gray columns show control values of four individual Epi-like differentiations starting from a 2iLIF unsorted population. Columns show mean ± SEM. Statistics show one-way ANOVA. ns, non-significant $^*p < 0.05$; $^{**}p < 0.01$.

(E) Immunofluorescence images showing one plane or a zoomed section of the ICM (maximum projection) of E4.5 blastocysts showing the H2B-tagged contribution from $SOX2^+$ or DP donor cells after morula aggregation assay. H2B contribution in magenta, CDX2 staining in yellow, and GATA6 staining in cyan. Scale bar, 20 μm. Arrows show contributing donor cells co-stained with GATA6 and not CDX2, meaning PrE contribution.

(F) Quantification of the E4.5 contribution after morula aggregation assay. n = 24 E4.5 blastocysts from $SOX2^+$ donor cells and 19 E4.5 blastocysts from DP donor cells. Epi, epiblast contribution; Epi+PrE, epiblast and PrE contribution; NC, no contribution.

(G) Immunofluorescence images showing a maximum projection of a representative E6.5 embryo showing the H2B-tagged contribution from $SOX2^+$ or DP donor cells after morula aggregation assay. H2B contribution in magenta. GATA6 staining in cyan. Arrows show contributing donor cells co-stained with GATA6 (extra-embryonic VE contribution). DAPI in white. Scale bar, 30 μm.

(H) Quantification of the E6.5 contribution after morula aggregation assay. n = 11 E6.5 embryos from $SOX2^+$ donor cells and 13 E6.5 embryos from DP donor cells. Epi, epiblast contribution; Epi+VE, epiblast and VE contribution; VE, VE contribution; NC, no contribution.

embryos), while DP cells were found in both the epiblast and PrE (13/19 embryos) (Figures 2E and 2F). We transferred aggregates to pseudo-pregnant mice and further assessed the contribution at E6.5. Here, we found that $SOX2^{+}$ donor cells contributed only to the epiblast (10/11 embryos), whereas DP cells contributed to both the extra-embryonic visceral endoderm (VE), marked with GATA6 staining, and epiblast (4/13 Epi and VE, 1/13 VE, and 8/13 Epi contribution) (Figures 2G and 2H). Taken together, these observations support the existence of a transient ICM-like state in KOSR culture.

Co-expression of SOX2 and GATA6 induces changes to canonical binding

To determine how these two antagonistic TFs could be co-expressed in KOSR and whether they might influence each other's binding or activity, we sought to identify the extent to which they recognize the same target in different populations. While considerable effort has been invested into understanding the pluripotency or epiblast network (Li and Belmonte, 2017), by comparison, little has been invested into PrE. To provide a framework by which we can understand the nature of this lineage bifurcation, we first established the enhancer network in differentiated PrE cells and compared it with the epiblast, as recapitulated in different pluripotent culture conditions. We assessed the enhancer network in nEnd stem cells and compared it with two distinct pluripotent conditions: 2iLIF and NACL. NACL is a defined pluripotent culture system where cells exhibit similar heterogeneity as conventional serum-containing media. In addition, NACL media is composed of the same set of cytokines as used for nEnd culture, but differs only in its base media (N2B27). We identified cell-type-specific enhancers by profiling the co-occupancy of the histone modifications H3K27ac and H3K4me1, the combination of which denotes enhancer activity (Calo and Wysocka, 2013) using Cleavage Under Targets & Release Using Nuclease (CUT&RUN) (Skene and Henikoff, 2017) (Figures 3A–3E). Based on the combination of these marks in two clonal cell lines in both pluripotent conditions, we identified 6,849 active pluripotency enhancers (Figures 3A–3C). In nEnd, we found 4,957 active PrE enhancers, with 1,434 of these being shared by both lineages, referred to as "common enhancers" (Figures 3B, 3D, and 3E). Motif analysis indicated that these regulatory regions are cell type specific as we identified specific groups of motifs enriched in each enhancer set. We observed that the PrE subset was highly enriched for GATA motifs, while the pluripotency subset was enriched for the OCT, NANOG, ESRRB, and KLF motifs. The common enhancer subset features a strong KLF signature, consistent with its expression in both lineages (Morgani and Brickman, 2015; Nowotschin et al., 2019) (Figure S4I). GO analysis for Biological Processes for the closest gene to the different enhancer subsets suggest that pluripotency enhancers were associated with LIF response and embryo development terms (Figure 3F), the PrE enhancers with membranes and adhesion (Figure 3G), and common enhancers represented general cellular processes (Figure 3H).

Having established the enhancer networks in both lineages, we assessed TF binding at both enhancer sets in both the final cell states (ESC and nEnd) and in the DP and in $SOX2^{+}$ populations cultured in KOSR. CUT&RUN was used to profile GATA6 in nEnd and DP populations and SOX2 to analyse NACL, 2iLIF and SOX2+ populations. To determine if SOX2 and GATA6 binding shifts globally, we compared SOX2 and GATA6 binding on pluripotency, PrE, and common enhancer sets (Figure 4A). While we observed a decrease in SOX2 binding to pluripotency enhancers in DP cells, we observed a significant recruitment of SOX2 to PrE enhancers alongside GATA6, suggesting that SOX2 binding moves toward potential GATA6 sites. We also observed a smaller acquisition of GATA6 peaks at pluripotency enhancers, slightly greater than GATA6 binding the same elements in nEnd (Figure 4A). We detect 1,716 peaks co-bound by SOX2 and GATA6 in the DP cells (Figure 4B), and 416 of these peaks (24%) sit at enhancers that we previously defined. The major cluster of co-bound peaks at enhancers (50%) sit at PrE enhancers, while only 28% of these are found at pluripotency enhancers and 22% at common enhancers. Taken together, this suggests that SOX2 is recruited to sites with GATA6 occupancy; when this takes place at an enhancer, it is predominantly at PrE enhancers. Given the PrE bias in occupancy, we analyzed the closest genes regulated *in vivo* (Boroviak et al., 2018) to these SOX2-GATA6 co-bound peaks and observed a 2-fold enrichment of PrE genes over epiblast genes. Moreover, the co-bound regions, regardless of their affiliation to lineage specific genes, contain twice as many GATA6 motifs as those found for SOX2 (Tables S1 and S2). Only around 10% of the closest genes to the co-bound peaks are significantly upregulated when DP cells are compared with $SOX2^{+}$ single-positive cells (specifically, 4.9% for epiblast genes and 11.7% for PrE genes) (Tables S1 and S2), suggesting that lineage priming is not occurring at the transcriptional level.

While our data on co-binding is not based on sequential precipitation, we took advantage of a recent study that explored a similar co-binding phenomena by sequential chromatin immunoprecipitation in response to GATA6 induction in ESCs to drive differentiation to iXEN cells (Thompson et al., 2022). We observed a significant percentage of overlap of our binding for SOX2 and GATA6 in DP cells with this study's early timepoints for GATA6 induction (Figure 4E; and Table S3). Here, we found that between 70% and 80% of our DP peaks overlap with the 2- and 4-h

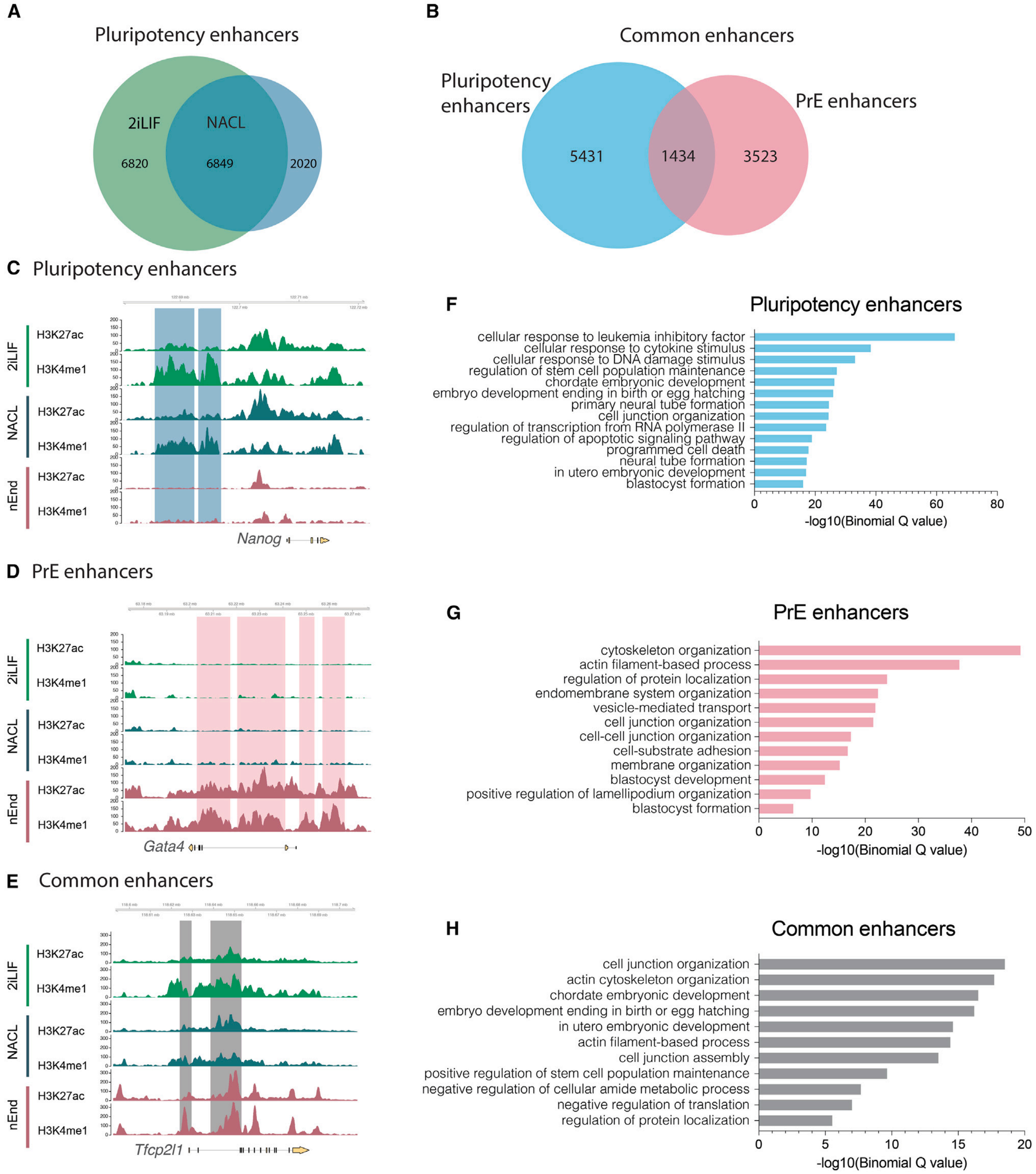

Figure 3. Enhancers governing self-renewing identity in naive endoderm and pluripotency

(A) Euler diagram defining an intersect of pluripotency enhancers: enhancers active in both 2iLIF and NACL.
(B) Euler diagram comparing pluripotency enhancers (intersect from A) with PrE enhancers.
(C–E) Genome browser tracks of H3K27ac and H3K4me1 across conditions 2iLIF, NACL, and nEnd of loci *Nanog* (C), *Gata4* (D), and *Tfcp2l1* (E) with examples of pluripotency enhancers (blue), PrE enhancers (pink), and common enhancers (gray) defined in (B).
(F–H) GO terms of the biological processes of pluripotency enhancers (F), PrE enhancers, (G) and common enhancers (H).

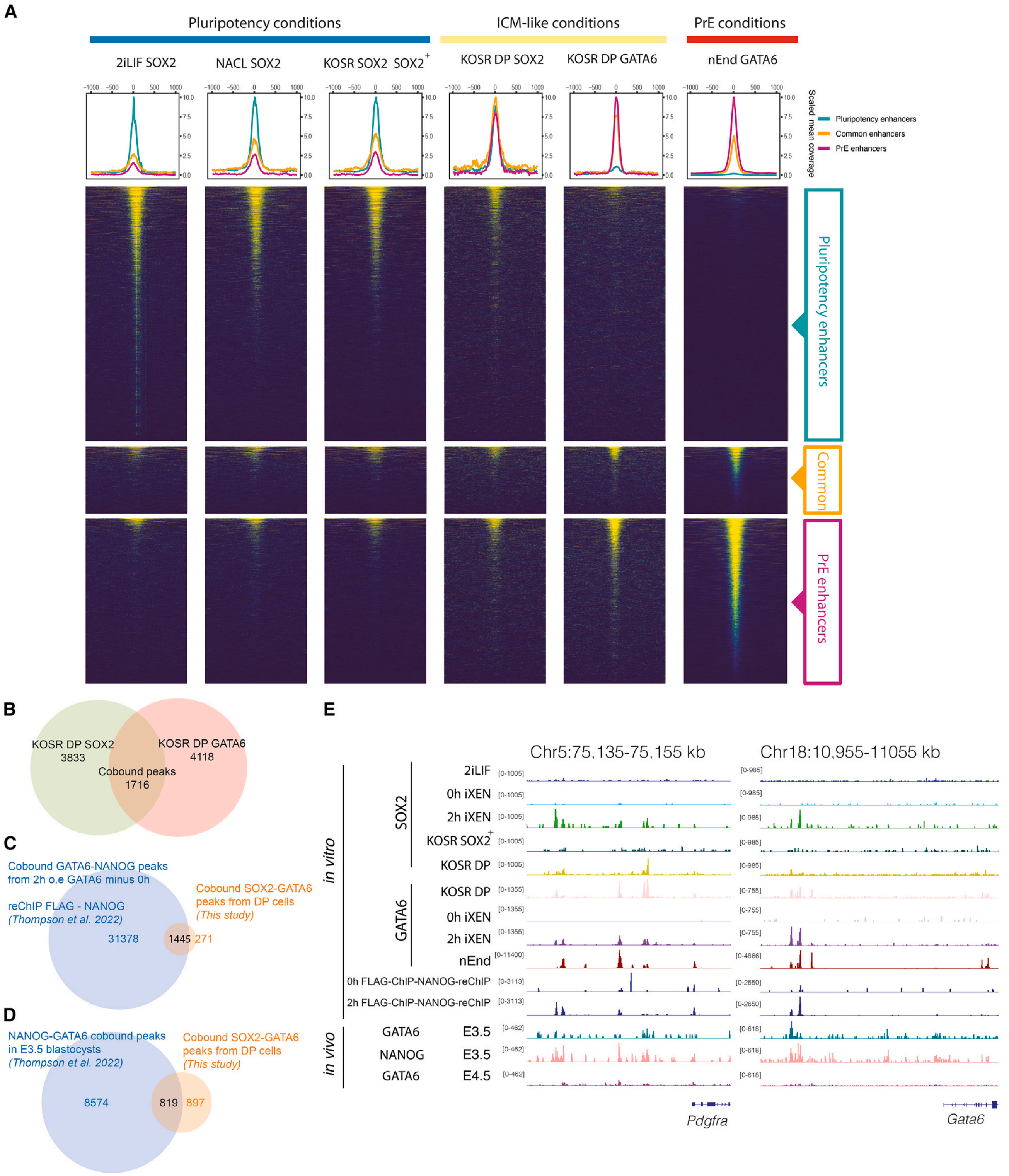

Figure 4. Reciprocal lineage priming by GATA6 and SOX2

(A) Scaled heatmaps showing SOX2 and GATA6 binding at the pluripotency, common, or PrE enhancer subsets defined previously, in the conditions: 2iLIF, NACL, KOSR sorted cells, and nEnd.

(B) Euler diagram of SOX2 and GATA6 peaks in KOSR DP cells.

(legend continued on next page)

overexpression time points for the factor GATA6 and 50%–60% for SOX2. We observe an impressive 84% overlap of our co-bound peaks with the NANOG-GATA6 co-bound peaks at 2 h based on sequential precipitation (GATA6-FLAG-NANOG) (Figures 4C and 4E), suggesting that our SOX2 and GATA6 are indeed binding at the same sites. We also observe a 48% overlap of our DP GATA6-SOX2 co-bound peaks with their NANOG-GATA6 co-bound peaks in the *in vivo* E3.5 blastocysts (Figure 4D). Moreover, assessment of these data also suggests that the preference of SOX2/GATA6 binding for PrE enhancers in our data may be a general property of cooperative binding between endoderm and epiblast determinants, as *in vivo* ICM GATA6-NANOG co-bound peaks also appear to more often bind in our endoderm enhancers (48%) compared with their presence at pluripotency ones (33%).

DISCUSSION

In this paper, we found that ICM-like expression of endoderm and epiblast TFs prepare differentiation not only for their own lineage, but for the opposing lineage as well. Cooperative binding interactions between SOX2 and GATA6 simultaneously prepare enhancers for activation, while maintaining these genes in a primed status enables rapid response in lineage bifurcation. Multipotent progenitors must maintain tight control of lineage commitment to ensure correctly proportioned embryonic development. Lineage-specific inducer genes, such as *Gata6* and *Sox2*, which are co-expressed in early ICM cells (Dietrich and Hiiragi, 2007; Morgani and Brickman, 2015), could prime later differentiation based on their ability to stabilize each other's binding and maintain cells with the ability to kick start either lineage-specific gene regulatory network.

DP cells can readily differentiate toward epiblast or PrE, while their sister cells in culture (SOX2$^+$ cells) seem to be biased toward epiblast. Transcriptionally and metabolically, DP cells also better approximate the E3.5 unsegregated ICM, whereas the SOX2$^+$ cells resemble the E4.5 epiblast. In agreement with Posfai et al. (2021), we observe that cells cultured in EPSCM have a more epiblast-like signature. Given that the ICM can sustain the co-expression of these factors over multiple cell divisions, the wiring of these cooperative interactions between cross-lineage TFs are likely to be stable; we find *in vitro* ICM-like cells can sustain this state through cell division as well. A recent study based on GATA6 over-expression describes an *in vitro* model for the ICM based on early time points of GATA6-mediated reprogramming of naive ESCs (Thompson et al., 2022). However, here we manage to capture a small population of cells that spontaneously enter this state in self-renewing culture without artificial manipulation of gene expression. The ability to sort this population at a steady state enabled us to correlate differentiation competence with TF co-occupancy. Further work is required to properly understand the ICM's environment, which in turn will lead to better ways to robustly expand these cells *in vitro*. But even as a small population, these cells seem to represent a genuine stem cell model that indefinitely maintains embryonic and extra-embryonic bipotency.

Steady-state culture pluripotency is supported by a set of enhancers that contain motifs for pluripotency factors, such as OCT4, SOX2, NANOG, KLFs, and ESRRB, while in nEnd the enhancers feature mostly GATA motifs. Common enhancers, which have H3K27ac and H3K4me1 present in both nEnd and naive cultures, are enriched for KLF motifs. While we observe GATA6 and SOX2 binding to all these enhancer sets, we find an almost identical bias in our data as that derived from the E3.5 ICM *in vivo* (Thompson et al., 2022) for PrE enhancers, as well as 2-fold increase in GATA6 motifs over SOX2 motifs. This suggests that GATA6 binds its consensus sites with a relatively high affinity and actively recruits SOX2, possibly via protein-protein interaction, rather than the other way round. This would seem to contrast its role in ESCs, where residence time data and *in vivo* imaging studies suggest that SOX2 drives OCT4 binding (Chen et al., 2014; White et al., 2016). Alternatively, this might reflect the ability of SOX2 to recognize lower affinity elements, like those recognized by SOX17, only in the presence of GATA6. Moreover, we observed the presence of SOX sites in the proximity of GATA in the PrE, but not the other way round. However, if this is the case, why should the presence of GATA6 still facilitate both endoderm and epiblast differentiation? Perhaps the

(C) Euler diagram showing the overlap of the GATA6-FLAG chromatin immunoprecipitation (ChIP) with NANOG reChIP (Thompson et al. 2022) with our SOX2-GATA6 co-bound peaks in DP cells.

(D) Euler diagram projecting the NANOG-GATA6 co-bound peaks from the *in vivo* data of the E3.5 blastocysts (Thompson et al. 2022) with the SOX2-GATA6 co-bound peaks in KOSR DP cells.

(E) Genome browser tracks of SOX2 and GATA6 comparing data from this study (2iLIF, KOSR, and nEnd tracks) with Thompson et al. (2022) (0 h and 2 h of iXEN induction, the GATA-FLAG ChIP with NANOG reChIP and the *in vivo* E3.5 and E4.5 blastocysts tracks). *Pdgfra* and *Gata6* loci. Tracks are scaled accordingly with antibody and experiment. All the *in vitro* SOX2 experiments are in the same scale, the *in vitro* GATA6 experiments are also in the same scale (apart from nEnd due to the extreme difference in PrE genes expression). FLAG-NANOG reChIP tracks are on the same scale, as well as the *in vivo* data.

relative level of free SOX2 is a key determinant of epiblast differentiation, and the presence of GATA6 titrates SOX2 away from epiblast enhancers and OCT4, maintaining a threshold concentration that can be pushed toward either endoderm or epiblast.

In hematopoietic differentiation, the co-expression of antagonistic lineage specifiers is thought to maintain progenitor populations at the apex of two lineages in a process referred to as multi-lineage priming (Graf and Enver, 2009; Hu et al., 1997). Here we find that cooperativity between GATA6 and SOX2 leads to alterations in their binding in DP cells, such that they are sitting at sites found in both lineage-specific enhancer sets. Presumably, there are either insufficient levels of these factors to drive differentiation or perhaps the expression of these two TFs in the absence of lineage determining signaling is insufficient to promote differentiation (Hamilton et al., 2019; Knudsen and Brickman, 2020). As there is little transcription of genes close to co-bound peaks, lineage-specific signaling may be required to drive transcription and occupancy by both TFs creates a situation in which the genes they regulate are in a ready response mode. That TFs bind to enhancers and prepare them for transcription in response to signaling to encode potency or remain bound following a signal to safeguard plasticity suggests that they are determinants of potential rather than actuality (Hamilton et al., 2019; Knudsen et al., 2023; Wong et al., 2023). In this instance, these factors bind together to enhancers that can drive transcription in either cell type, providing a head start for either lineage, but without triggering differentiation. Thus, multi-lineage priming may be about manipulating threshold distributions for lineage specification, exploiting antagonistic factors with interactions that can be manifest at the level of cooperative binding. In this way, these factors support cells in a precarious balance poised for either of two opposing fates that can be readily induced by alterations to signaling that promote lineage specific transcription.

EXPERIMENTAL PROCEDURES

Resource availability

Corresponding author

Further information and requests for resources and reagents should be directed to the corresponding author Joshua M Brickman joshua.brickman@sund.ku.dk.

Materials availability

Reagents generated in this study are available upon reasonable request to the corresponding author.

Data and code availability

scRNA-seq and CUT&RUN data that support the findings of this study have been deposited in NCBI GEO under accession number GSE227889 (https://www.ncbi.nlm.nih.gov/geo/query/acc.cgi?acc=GSE227889).

The full scRNA-seq analysis can be found at https://github.com/brickmanlab/riveiro-et-al-2023/.

Previously published Nowotschin et al., 2019 and Thompson et al., 2022 datasets that were used here are available in the NCBI GEO under accession numbers GSE123046 and GSE181104.

ESC culture

ESCs were generated using E14JU ESCs from the 129/Ola background. ESC lines were maintained in serum/LIF Canham et al. (2010), 2iLIF (Ying et al., 2008), NACL (Anderson et al., 2017), KOSR (Martin Gonzalez et al., 2016), EPSCM (Yang et al., 2017a), and LCDM (Yang et al., 2017b) as previously described.

Generation of SGGC ESC lines

SOX2-GFP ESCs (Anderson et al., 2017) clone SG16 was used to further target with a GATA6-mCherry construct using CRISPR-Cas technology. The construct contains mCherry tagged immediately after exon 7 of the *Gata6* locus, just before the STOP codon, plus 3,000-bp homology arms. We obtained three clones (B9, B12 and E1) that were successfully integrated.

SGGC cells were verified by performing immunostaining for SOX2, GATA6, mCherry and GFP, western blot, locus Sanger sequencing to screen for unwanted mutations generated by CRISPR, karyotyping, and Southern blot (Figures S1A–S1E). Resistance cassettes can be easily removed using Cre-mediated recombination; however, we decided to use the original clones with the resistance cassettes included. All three clones give the same reproducible results. All three clones were used for scRNA-seq. Clones B9 and B12 presented the highest amount of DP cells, so these two clones were used in all CUT&RUN, differentiation, and morula aggregation experiments. Detailed characterization of the SGGC cell line is described in the figure legends and supplemental information.

Flow cytometry and FACS

Cells were analyzed using an LSR Fortessa flow cytometer (BD Biosciences) with FACSDiva (BD Biosciences) software. Plots were generated using FCS Express 6.0 (DeNovo Software). Cells were sorted by SOX2$^+$ or DP populations using a BD FACS Aria III (BD FACSDiva Software version 8) with a 100-μm nozzle. Further details are contained in the supplemental information.

Differentiation

ESCs were cultured for four passages in KOSR prior to differentiation, isolated by FACS for DP or SOX2$^+$ expression, and seeded at the same cell density. Upon attachment, media was replaced for specific differentiation conditions. For nEnd differentiation, we plated 6×10^4 cells/cm^2 in gelatinized plates and cultured the cells in RACL media as previously described (Anderson et al., 2017; Linneberg-Agerholm et al., 2019). For Epi-like differentiation, we plated 20×10^4 cells/cm^2 in fibronectin-coated plates and cultured the cells in Epi-like media for 3 days (Hayashi et al., 2011).

For trophoblast stem cell differentiation, we plated 10×10^4 cells/cm^2 and then transferred to trophoblast stem cell medium for a total of 6 days (Tanaka et al., 1998). Antibody staining,

RT-PCR, and alkaline phosphatase are described in the supplemental information.

scRNA-seq

Cells were sorted directly into 384-well plates containing lysis buffer, which includes the first RT primer and RNase inhibitor, then immediately frozen and later processed by the MARS-seq2 protocol (Keren-Shaul et al., 2019). scRNA-seq libraries were sequenced using Illumina NextSeq500 at a median sequencing depth of 225,000 reads per single cell. Pre-processing was done using the nfcore/marsseq pipeline (Proks et al., 2023) with the following command: nextflow run nf-core/marsseq -r 1.0.3 -profile ku_sund_dangpu –with-tower –genome mm10 –velocity –input SCR_20221006/raw/samplesheet.csv –outdir/scratch/ALBA_SB2/.

Both *in vivo* (Nowotschin et al., 2019) and *in vitro* datasets were independently processed using SCANPY (v1.9.3) (Wolf et al., 2018). The MARS-seq2 *in vitro* dataset was filtered to include cells containing between 1,000 and 45,000 unique molecular identifiers (UMIs) representing between 1,400 and 7,500 genes. The reference *in vivo* dataset was filtered to exclude cells with fewer than 10 UMIs and 10 genes and to only contain cells annotated as originating from E3.5 and E4.5 mouse stages. Empty control wells labeled as zero and ERCC-genes were also discarded. The filtered *in vitro* and *in vivo* datasets contained 1,139 cells and 1,006 cells, respectively. Raw counts were then depth normalized and Log1p transformed. For downstream analysis, highly variable genes were identified and a reduced dimension UMAP representation was computed for first 30 PCAs, followed by Leiden unsupervised clustering, which estimated four clusters with a set resolution of 0.4. The top differentially expressed genes were identified using a Benjamini-Hochberg-corrected t test, with the following cutoffs ($\log_2$(fold change) > 1 and adjusted $p < 0.05$). Mapping of the *in vitro* data onto the *in vivo* mouse E3.5 and E4.5 dataset was performed using the 'ingest' function of SCANPY. PCA of this dataset, and subsequent UMAP dimension reduction, was performed using an intersection of the top highly variable genes. The χ^2 test, implemented in SciPy (Virtanen et al., 2020), was used to determine significance for differences in observed proportions of experimental conditions or clusters. The seaborn library (Waskom, 2021) was used for visualization of normalized residuals.

Time-lapse imaging and cell tracking

SGGCH2B ESC lines (SGGC lines tagged with H2B-miRFP670) were cultured in KOSR media, on 8-well slides (Ibidi) and imaged every 15 min across 72 h. mCherry, GFP, and CY5 fluorescent light channels were recorded in 5% CO_2, 20% O_2 at 37°C under a Deltavision Widefield Screening microscope. ESCs were seeded at 5,000 cells/cm^2 24 h before the beginning of the time lapse in KOSR. We performed manual cell tracking using Imaris v9.5 (Bitplane). Nuclei were segmented using the H2B marker. We measured the SOX2-GFP and GATA6-mCherry fluorescence intensities of a circular area of a 50-μm diameter inside the segmented nuclei. For each area measured, we took the median fluorescence intensity as the measure for that given data point. Intensity measurements were linked to its time point and lineage, allowing us to infer the division time for each cell that was tracked, as well as the expression level of both SOX2 and GATA6 in each time point. Only cells with completed cell cycle information were used for calculating the transition analysis. A total of 63 individual tracks of 72 h have been tracked.

CUT&RUN

KOSR, nEnd, 2iLIF, or NACL cells were grown in their respective media for at least four passages. A minimum of 100,000 cells were sorted to proceed with the CUT&RUN protocol. CUT&RUN was performed using an in house purified MNase and following the published protocol (Janssens and Henikoff, 2019). Library preparation was performed following this published protocol (Liu, 2019).

Reads were pre-processed using Cutadapt (Martin, 2011), Bowtie2 (Langmead and Salzberg, 2012), PICARD 2, SAMtools (Li et al., 2009), BEDtools (Quinlan and Hall, 2010), and DeepTools (Ramírez et al., 2016). All downstream data analysis was performed using BEDtools, SAMtools, DeepTools, Fluff (Georgiou and van Heeringen, 2016), Integrative Genome Viewer (IGV 2.16.0) (Robinson et al., 2011), SEACR (Meers et al., 2019), HOMER (Heinz et al., 2010), and RStudio (RStudio, 2016). Peaks were called using SEACR with the parameters: 'relaxed' for TFs and 'Stringent' for histone marks. Peaks were called against a negative IgG control, generated in each experiment for each condition. Further details can be found in the supplemental methods.

Chimera assays

For chimera assays, H2B-miRFP670 tagged clones SGGCH2B B9.A and B12.B were used. Cells were sorted for SOX2$^+$ or DP as previously described. Further details about morula aggregation protocol can be found in supplemental methods. Animal work was carried in accordance with European legislation. All work was authorized by and carried out under Project License 2018-15-0201-01520 and 2023-15-0201-01513 issued by the Danish Regulatory Authority.

SUPPLEMENTAL INFORMATION

Supplemental information can be found online at https://doi.org/10.1016/j.stemcr.2023.12.002.

ACKNOWLEDGMENTS

We thank Paul van Dieken and Gelo de la Cruz for flow cytometry and FACS support and expertise. We thank Michaela M. Rothová, Helen Neil, and Magali Michaut for technical expertise and support with the scRNA-seq and CUT&RUN sequencing. We thank Javier Martin Gonzalez, Ricardo Alonso Laguna Barraza, and all the transgenics platform facility for technical assistance and providing mouse embryos. We thank Jutta Bulkescher and the reNEW Imaging Platform for training, technical expertise, support, and the use of microscopes. We thank Jose Alejandro Herrera Romero and Sarah Louise Lundregan for bioinformatics advice. Last, we thank the entire Brickman laboratory, especially Molly P. Lowndes and Robert A. Bone, for critical discussion. Work in the Brickman laboratory was supported by Lundbeck Foundation (R198-2015-412, R370-2021-617 and R400-2022-769), Independent Research Fund Denmark (DFF-8020-00100B, DFF-0134-00022B, and DFF-2034-00025B), and the Danish National

Research Foundation (DNRF116). A.R.R. and M.Pe. were supported by Lundbeck Foundation PhD studentships (R208-2015-2872 and R286-2018-1534). The Novo Nordisk Foundation Center for Stem Cell Medicine (reNEW) is supported by the Novo Nordisk Foundation, grant number NNF21CC0073729, and previously NNF17CC0027852.

AUTHOR CONTRIBUTIONS

A.R.R., J.A.M., and M.L.A. performed and analyzed experiments; M.Pe., M.Pr., and N.S. analyzed experiments; J.M.B. supervised the project; A.R.R. and J.M.B. designed the project and wrote the paper with input from all other authors.

DECLARATION OF INTERESTS

We declare no competing interests.

Received: November 6, 2023
Revised: December 10, 2023
Accepted: December 11, 2023
Published: January 11, 2024

REFERENCES

Anderson, K.G.V., Hamilton, W.B., Roske, F.V., Azad, A., Knudsen, T.E., Canham, M.A., Forrester, L.M., and Brickman, J.M. (2017). Insulin fine-tunes self-renewal pathways governing naive pluripotency and extra-embryonic endoderm. Nat. Cell Biol. *19*, 1164–1177.

Boroviak, T., Stirparo, G.G., Dietmann, S., Hernando-Herraez, I., Mohammed, H., Reik, W., Smith, A., Sasaki, E., Nichols, J., and Bertone, P. (2018). Single cell transcriptome analysis of human, marmoset and mouse embryos reveals common and divergent features of preimplantation development. Dev. Camb. Engl. *145*, dev167833.

Calo, E., and Wysocka, J. (2013). Modification of Enhancer Chromatin: What, How, and Why? Mol. Cell *49*, 825–837.

Canham, M.A., Sharov, A.A., Ko, M.S.H., and Brickman, J.M. (2010). Functional Heterogeneity of Embryonic Stem Cells Revealed through Translational Amplification of an Early Endodermal Transcript. PLoS Biol. *8*, e1000379.

Chen, J., Zhang, Z., Li, L., Chen, B.-C., Revyakin, A., Hajj, B., Legant, W., Dahan, M., Lionnet, T., Betzig, E., et al. (2014). Single-Molecule Dynamics of Enhanceosome Assembly in Embryonic Stem Cells. Cell *156*, 1274–1285.

Coronado, D., Godet, M., Bourillot, P.-Y., Tapponnier, Y., Bernat, A., Petit, M., Afanassieff, M., Markossian, S., Malashicheva, A., Iacone, R., et al. (2013). A short G1 phase is an intrinsic determinant of naïve embryonic stem cell pluripotency. Stem Cell Res. *10*, 118–131.

Dietrich, J.-E., and Hiiragi, T. (2007). Stochastic patterning in the mouse pre-implantation embryo. Dev. Camb. Engl. *134*, 4219–4231.

Garcia-Gonzalo, F.R., and Izpisúa Belmonte, J.C. (2008). Albumin-Associated Lipids Regulate Human Embryonic Stem Cell Self-Renewal. PLoS One *3*, e1384.

Genet, M., and Torres-Padilla, M.-E. (2020). The molecular and cellular features of 2-cell-like cells: a reference guide. Dev. Camb. Engl. *147*, dev189688.

Georgiou, G., and van Heeringen, S.J. (2016). fluff: exploratory analysis and visualization of high-throughput sequencing data. PeerJ *4*, e2209.

Graf, T., and Enver, T. (2009). Forcing cells to change lineages. Nature *462*, 587–594.

Hamilton, W.B., Mosesson, Y., Monteiro, R.S., Emdal, K.B., Knudsen, T.E., Francavilla, C., Barkai, N., Olsen, J.V., and Brickman, J.M. (2019). Dynamic lineage priming is driven via direct enhancer regulation by ERK. Nature *575*, 355–360.

Hayashi, K., Ohta, H., Kurimoto, K., Aramaki, S., and Saitou, M. (2011). Reconstitution of the Mouse Germ Cell Specification Pathway in Culture by Pluripotent Stem Cells. Cell *146*, 519–532.

Heinz, S., Benner, C., Spann, N., Bertolino, E., Lin, Y.C., Laslo, P., Cheng, J.X., Murre, C., Singh, H., and Glass, C.K. (2010). Simple Combinations of Lineage-Determining Transcription Factors Prime cis-Regulatory Elements Required for Macrophage and B Cell Identities. Mol. Cell *38*, 576–589.

Hu, M., Krause, D., Greaves, M., Sharkis, S., Dexter, M., Heyworth, C., and Enver, T. (1997). Multilineage gene expression precedes commitment in the hemopoietic system. Genes Dev. *11*, 774–785.

Janssens, D., and Henikoff, S. (2019). CUT&RUN: Targeted in Situ Genome-wide Profiling with High Efficiency for Low Cell Numbers. protocols.io.

Keren-Shaul, H., Kenigsberg, E., Jaitin, D.A., David, E., Paul, F., Tanay, A., and Amit, I. (2019). MARS-seq2.0: an experimental and analytical pipeline for indexed sorting combined with single-cell RNA sequencing. Nat. Protoc. *14*, 1841–1862.

Knudsen, T.E., and Brickman, J.M. (2020). Can a Cell Put Its Arms around a Memory? Cell Stem Cell *26*, 609–610.

Knudsen, T.E., Hamilton, W.B., Proks, M., Lykkegaard, M., Linneberg-Agerholm, M., Nielsen, A.V., Perera, M., Malzard, L.L., Trusina, A., and Brickman, J.M. (2023). A bipartite function of ESRRB can integrate signaling over time to balance self-renewal and differentiation. Cell Syst. *14*, 788–805.e8.

Langmead, B., and Salzberg, S.L. (2012). Fast gapped-read alignment with Bowtie 2. Nat. Methods *9*, 357–359.

Leese, H.J. (2012). Metabolism of the preimplantation embryo: 40 years on. Reproduction *143*, 417–427.

Li, M., and Belmonte, J.C.I. (2017). Ground rules of the pluripotency gene regulatory network. Nat. Rev. Genet. *18*, 180–191.

Li, H., Handsaker, B., Wysoker, A., Fennell, T., Ruan, J., Homer, N., Marth, G., Abecasis, G., and Durbin, R.; 1000 Genome Project Data Processing Subgroup (2009). The Sequence Alignment/Map format and SAMtools. Bioinformatics *25*, 2078–2079.

Linneberg-Agerholm, M., Wong, Y.F., Herrera, J.A.R., Monteiro, R.S., and Anderson, K.G.V. (2019). Naïve human pluripotent stem cells respond to Wnt, Nodal and LIF signalling to produce expandable naïve extra-embryonic endoderm. Development *146, dev180620*.

Liu, N. (2019). Library Prep for CUT&RUN with NEBNext® UltraTM II DNA Library Prep Kit for Illumina® (E7645). *protocols.io*.

Martin, M. (2011). Cutadapt removes adapter sequences from high-throughput sequencing reads. EMBnet. j. *17*, 10.

Martin Gonzalez, J., Morgani, S.M., Bone, R.A., Bonderup, K., Abelchian, S., Brakebusch, C., and Brickman, J.M. (2016). Embryonic Stem Cell Culture Conditions Support Distinct States Associated with Different Developmental Stages and Potency. Stem Cell Rep. *7*, 177–191.

Meers, M.P., Tenenbaum, D., and Henikoff, S. (2019). Peak calling by Sparse Enrichment Analysis for CUT&RUN chromatin profiling. Epigenet. Chromatin *12*, 42.

Morgani, S.M., and Brickman, J.M. (2015). LIF supports primitive endoderm expansion during pre-implantation development. Development *142*, 3488–3499.

Morgani, S.M., Canham, M.A., Nichols, J., Sharov, A.A., Migueles, R.P., Ko, M.S.H., and Brickman, J.M. (2013). Totipotent Embryonic Stem Cells Arise in Ground-State Culture Conditions. Cell Rep. *3*, 1945–1957.

Morgani, S., Nichols, J., and Hadjantonakis, A.-K. (2017). The many faces of Pluripotency: *in vitro* adaptations of a continuum of *in vivo* states. BMC Dev. Biol. *17*, 7.

Nichols, J., and Smith, A. (2011). The origin and identity of embryonic stem cells. Development *138*, 3–8.

Nowotschin, S., Setty, M., Kuo, Y.-Y., Liu, V., Garg, V., Sharma, R., Simon, C.S., Saiz, N., Gardner, R., Boutet, S.C., et al. (2019). The emergent landscape of the mouse gut endoderm at single-cell resolution. Nature *569*, 361–367.

Perera, M., Nissen, S.B., Proks, M., Pozzi, S., Monteiro, R.S., Trusina, A., and Brickman, J.M. (2022). Transcriptional Heterogeneity and Cell Cycle Regulation as Central Determinants of Primitive Endoderm Priming. Elife 11, e78967.

Pietenpol, J.A., and Stewart, Z.A. (2002). Cell cycle checkpoint signaling:: Cell cycle arrest versus apoptosis. Toxicology *181–182*, 475–481.

Posfai, E., Schell, J.P., Janiszewski, A., Rovic, I., Murray, A., Bradshaw, B., Yamakawa, T., Pardon, T., El Bakkali, M., Talon, I., et al. (2021). Evaluating totipotency using criteria of increasing stringency. Nat. Cell Biol. *23*, 49–60.

Proks, M., Herrera, J.A.R., Sedzinski, J., and Brickman, J.M. (2023). nf-core/marsseq: systematic pre-processing pipeline for MARS-seq experiments. 2023.06.28.546862.

Ramírez, F., Ryan, D.P., Grüning, B., Bhardwaj, V., Kilpert, F., Richter, A.S., Heyne, S., Dündar, F., and Manke, T. (2016). deepTools2: a next generation web server for deep-sequencing data analysis. Nucleic Acids Res. *44*, W160–W165.

Riveiro, A.R., and Brickman, J.M. (2020). From pluripotency to totipotency: an experimentalist's guide to cellular potency. Development *147*, dev189845.

Robinson, J.T., Thorvaldsdóttir, H., Winckler, W., Guttman, M., Lander, E.S., Getz, G., and Mesirov, J.P. (2011). Integrative genomics viewer. Nat. Biotechnol. *29*, 24–26.

Skene, P.J., and Henikoff, S. (2017). An efficient targeted nuclease strategy for high-resolution mapping of DNA binding sites. Elife *6*, e21856.

Tanaka, S., Kunath, T., Hadjantonakis, A.-K., Nagy, A., and Rossant, J. (1998). Promotion of Trophoblast Stem Cell Proliferation by FGF4. Science *282*, 2072–2075.

Thompson, J.J., Lee, D.J., Mitra, A., Frail, S., Dale, R.K., and Rocha, P.P. (2022). Extensive co-binding and rapid redistribution of NANOG and GATA6 during emergence of divergent lineages. Nat. Commun. *13*, 4257.

Virtanen, P., Gommers, R., Oliphant, T.E., Haberland, M., Reddy, T., Cournapeau, D., Burovski, E., Peterson, P., Weckesser, W., Bright, J., et al. (2020). SciPy 1.0: fundamental algorithms for scientific computing in Python. Nat. Methods *17*, 261–272.

Waskom, M. (2021). seaborn: statistical data visualization. J. Open Source Softw. *6*, 3021.

White, M.D., Angiolini, J.F., Alvarez, Y.D., Kaur, G., Zhao, Z.W., Mocskos, E., Bruno, L., Bissiere, S., Levi, V., and Plachta, N. (2016). Long-Lived Binding of Sox2 to DNA Predicts Cell Fate in the Four-Cell Mouse Embryo. Cell *165*, 75–87.

Wolf, F.A., Angerer, P., and Theis, F.J. (2018). SCANPY: large-scale single-cell gene expression data analysis. Genome Biol. *19*, 15.

Wong, Y.F., Kumar, Y., Proks, M., Herrera, J.A.R., Rothová, M.M., Monteiro, R.S., Pozzi, S., Jennings, R.E., Hanley, N.A., Bickmore, W.A., and Brickman, J.M. (2023). Expansion of ventral foregut is linked to changes in the enhancer landscape for organ-specific differentiation. Nat. Cell Biol. *25*, 481–492.

Yang, J., Ryan, D.J., Wang, W., Tsang, J.C.H., Lan, G., Masaki, H., Gao, X., Antunes, L., Yu, Y., Zhu, Z., et al. (2017a). Establishment of mouse expanded potential stem cells. Nature *550*, 393–397.

Yang, Y., Liu, B., Xu, J., Wang, J., Wu, J., Shi, C., Xu, Y., Dong, J., Wang, C., Lai, W., et al. (2017b). Derivation of Pluripotent Stem Cells with In Vivo Embryonic and Extraembryonic Potency. Cell *169*, 243–257.e25.

Ying, Q.L., Wray, J., Nichols, J., Batlle-Morera, L., Doble, B., Woodgett, J., Cohen, P., and Smith, A. (2008). The ground state of embryonic stem cell self-renewal. Nature *453*, 519–523.

Quinlan, A.R., and Hall, I.M. (2010). BEDTools: a flexible suite of utilities for comparing genomic features. Bioinformatics. *26*, 841–842.

Stem Cell Reports, Volume *19*

Supplemental Information

Transcription factor co-expression mediates lineage priming for embryonic and extra-embryonic differentiation

Alba Redó-Riveiro, Jasmina Al-Mousawi, Madeleine Linneberg-Agerholm, Martin Proks, Marta Perera, Nazmus Salehin, and Joshua M. Brickman

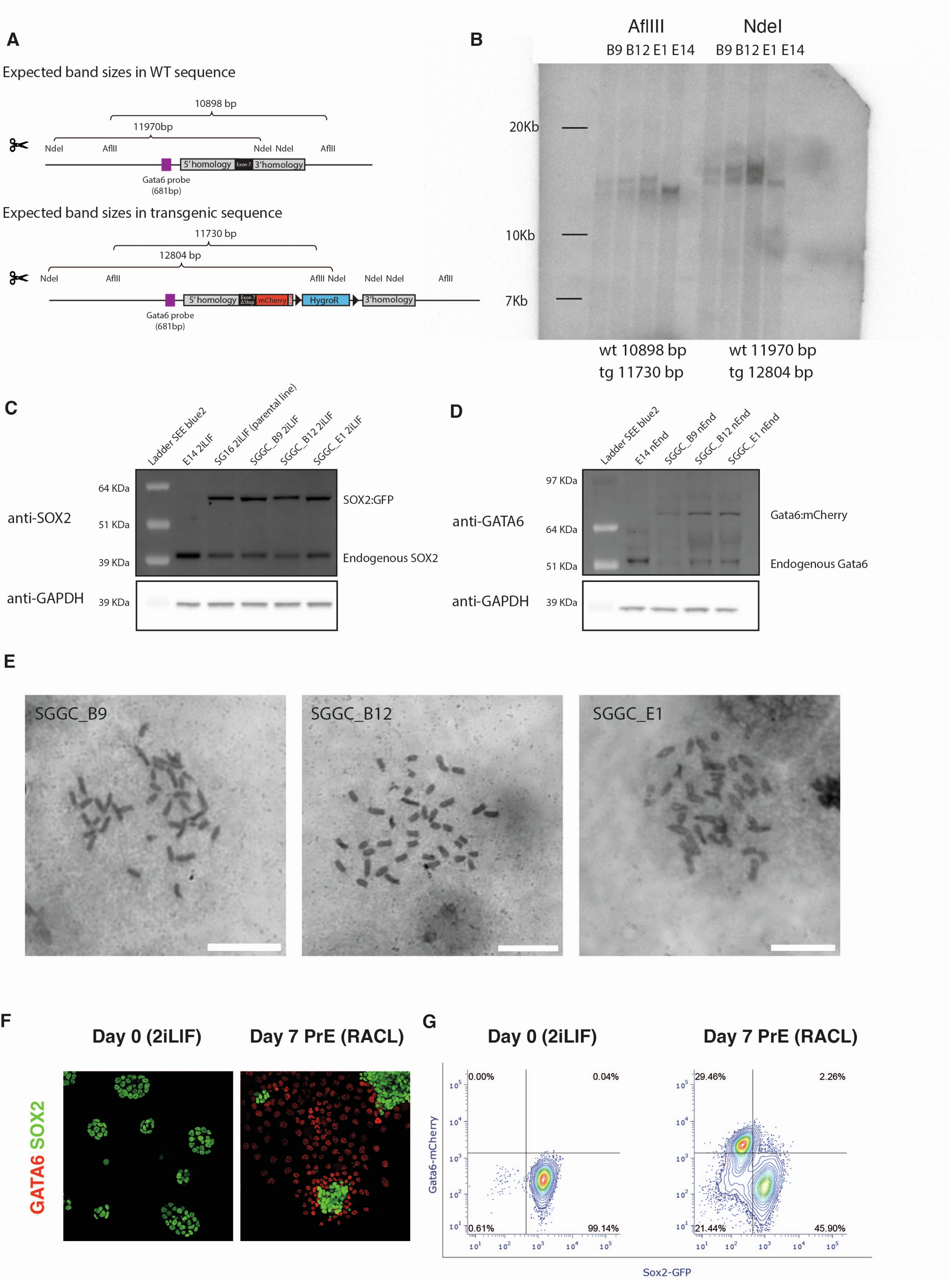

A
Expected band sizes in WT sequence
10898 bp
11970bp
NdeI
AflII
NdeI NdeI
AflII
5'homology
Exon 7
3'homology
Gata6 probe (681bp)
Expected band sizes in transgenic sequence
11730 bp
12804 bp
NdeI
AflII
AflII NdeI
NdeI NdeI
AflII
5' homology
mCherry
HygroR
3'homology
Gata6 probe (681bp)
B
AflIII
NdeI
B9 B12 E1 E14
B9 B12 E1 E14
20Kb
10Kb
7Kb
wt 10898 bp
tg 11730 bp
wt 11970 bp
tg 12804 bp
C
Ladder SEE blue2
E14 2iLIF
SG16 2iLIF (parental line)
SGGC_B9 2iLIF
SGGC_B12 2iLIF
SGGC_E1 2iLIF
anti-SOX2
64 KDa
51 KDa
39 KDa
SOX2:GFP
Endogenous SOX2
anti-GAPDH
39 KDa
D
Ladder SEE blue2
E14 nEnd
SGGC_B9 nEnd
SGGC_B12 nEnd
SGGC_E1 nEnd
anti-GATA6
97 KDa
64 KDa
51 KDa
Gata6:mCherry
Endogenous Gata6
anti-GAPDH
39 KDa
E
SGGC_B9
SGGC_B12
SGGC_E1
F
Day 0 (2iLIF)
Day 7 PrE (RACL)
GATA6 SOX2
G
Day 0 (2iLIF)
Day 7 PrE (RACL)
0.00%
0.04%
0.61%
99.14%
29.46%
2.26%
21.44%
45.90%
Gata6-mCherry
Sox2-GFP

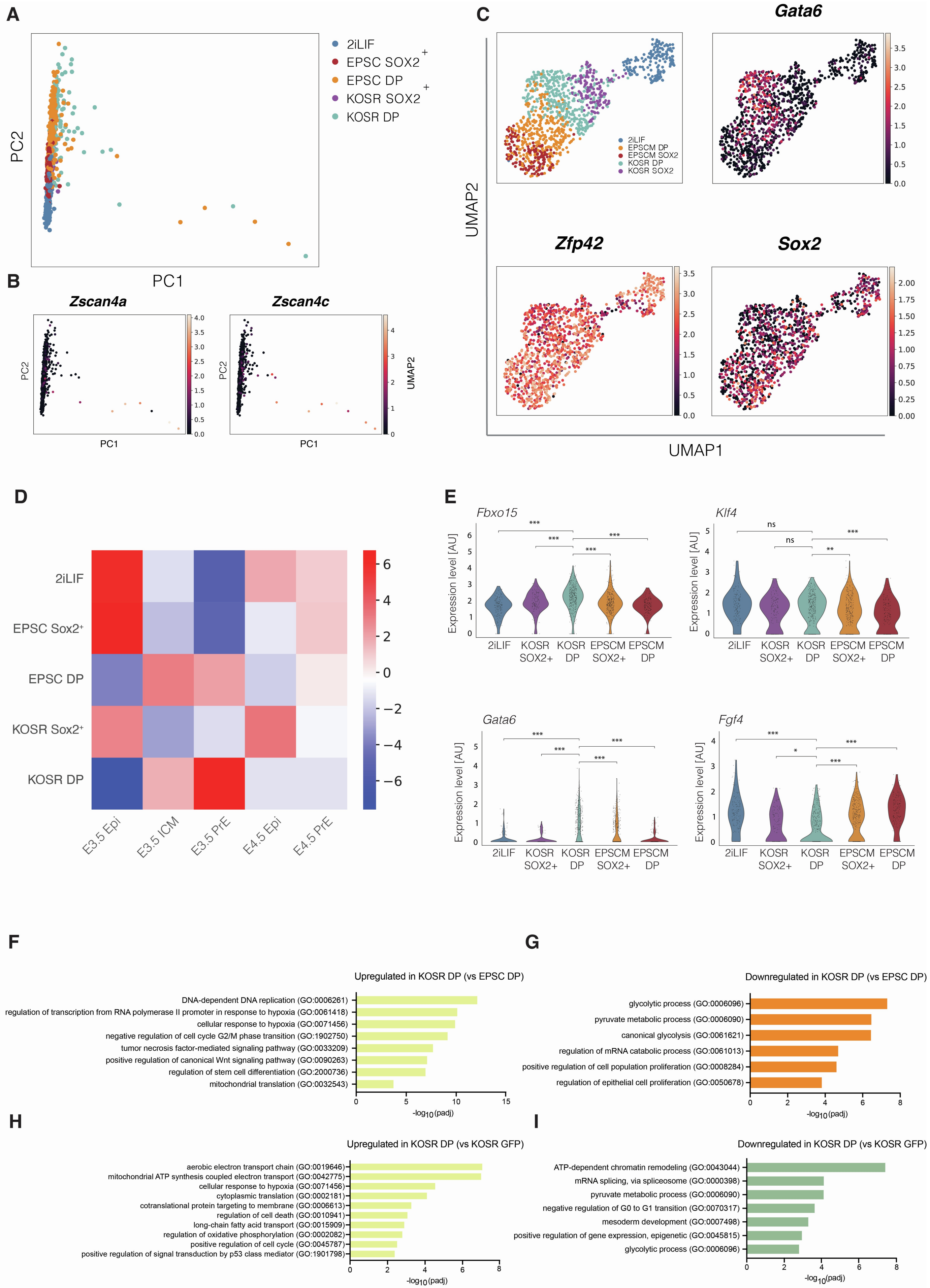
A
2iLIF
EPSC SOX2+
EPSC DP
KOSR SOX2+
KOSR DP
PC2
PC1
B
Zscan4a
Zscan4c
PC2
PC1
UMAP2
C
Gata6
Zfp42
Sox2
2iLIF
EPSCM DP
EPSCM SOX2
KOSR DP
KOSR SOX2
UMAP2
UMAP1
D
2iLIF
EPSC Sox2+
EPSC DP
KOSR Sox2+
KOSR DP
E3.5 Epi
E3.5 ICM
E3.5 PrE
E4.5 Epi
E4.5 PrE
E
Fbxo15
Klf4
Gata6
Fgf4
Expression level [AU]
2iLIF
KOSR SOX2+
KOSR DP
EPSCM SOX2+
EPSCM DP
F
Upregulated in KOSR DP (vs EPSC DP)
DNA-dependent DNA replication (GO:0006261)
regulation of transcription from RNA polymerase II promoter in response to hypoxia (GO:0061418)
cellular response to hypoxia (GO:0071456)
negative regulation of cell cycle G2/M phase transition (GO:1902750)
tumor necrosis factor-mediated signaling pathway (GO:0033209)
positive regulation of canonical Wnt signaling pathway (GO:0090263)
regulation of stem cell differentiation (GO:2000736)
mitochondrial translation (GO:0032543)
-log10(padj)
G
Downregulated in KOSR DP (vs EPSC DP)
glycolytic process (GO:0006096)
pyruvate metabolic process (GO:0006090)
canonical glycolysis (GO:0061621)
regulation of mRNA catabolic process (GO:0061013)
positive regulation of cell population proliferation (GO:0008284)
regulation of epithelial cell proliferation (GO:0050678)
-log10(padj)
H
Upregulated in KOSR DP (vs KOSR GFP)
aerobic electron transport chain (GO:0019646)
mitochondrial ATP synthesis coupled electron transport (GO:0042775)
cellular response to hypoxia (GO:0071456)
cytoplasmic translation (GO:0002181)
cotranslational protein targeting to membrane (GO:0006613)
regulation of cell death (GO:0010941)
long-chain fatty acid transport (GO:0015909)
regulation of oxidative phosphorylation (GO:0002082)
positive regulation of cell cycle (GO:0045787)
positive regulation of signal transduction by p53 class mediator (GO:1901798)
-log10(padj)
I
Downregulated in KOSR DP (vs KOSR GFP)
ATP-dependent chromatin remodeling (GO:0043044)
mRNA splicing, via spliceosome (GO:0000398)
pyruvate metabolic process (GO:0006090)
negative regulation of G0 to G1 transition (GO:0070317)
mesoderm development (GO:0007498)
positive regulation of gene expression, epigenetic (GO:0045815)
glycolytic process (GO:0006096)
-log10(padj)

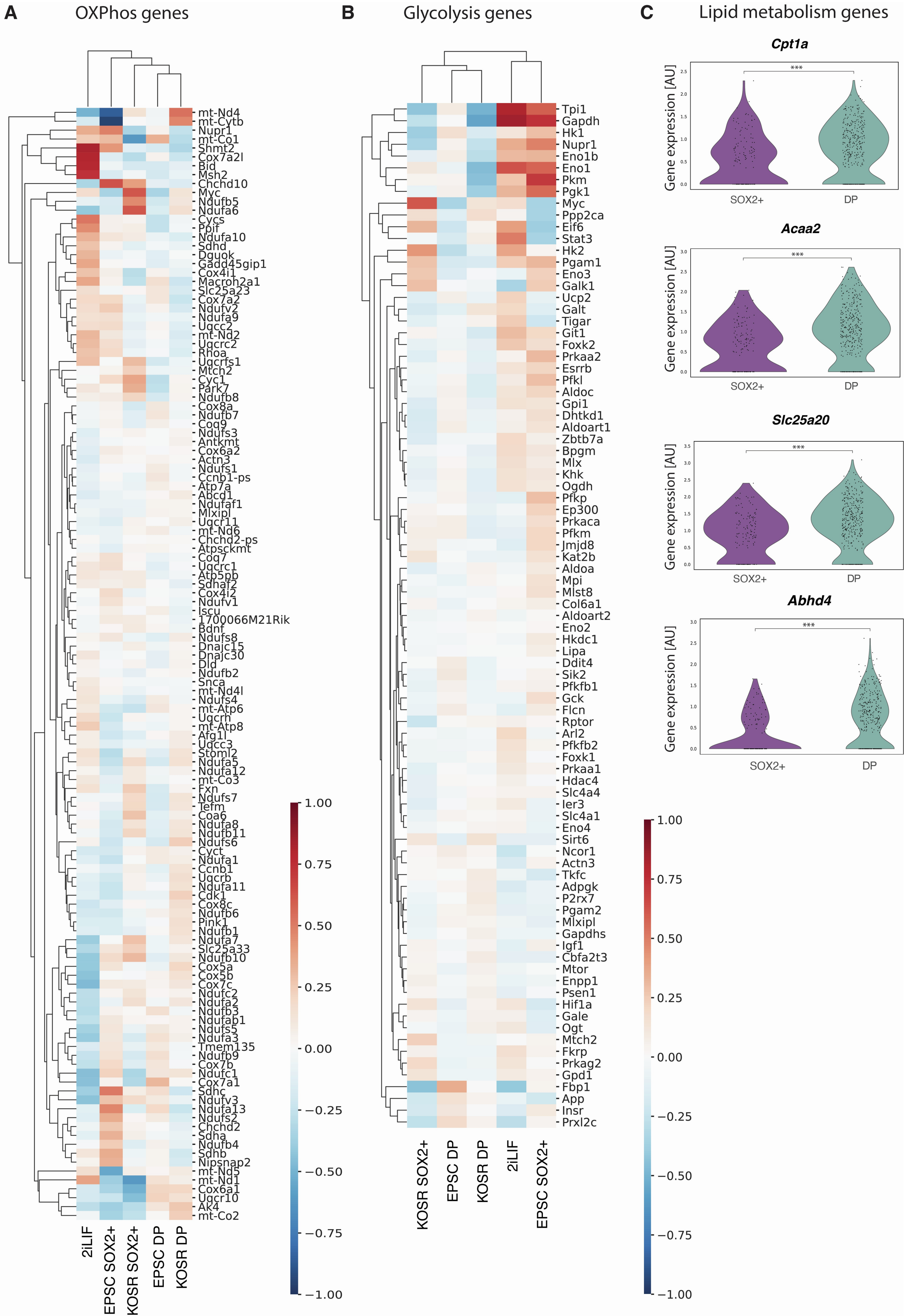
A
OXPhos genes
mt-Nd4
mt-Cytb
Nupr1
mt-Co1
Shmt2
Cox7a2l
Bid
Msh2
Chchd10
Myc
Ndufb5
Ndufa6
Cycs
Ppif
Ndufa10
Sdhd
Dguok
Gadd45gip1
Cox4i1
Macroh2a1
Slc25a23
Cox7a2
Ndufv2
Ndufa9
Uqcc2
mt-Nd2
Uqcrc2
Rhoa
Uqcrfs1
Mtch2
Cyc1
Park7
Ndufb8
Cox8a
Ndufb7
Coq9
Ndufs3
Antkmt
Cox6a2
Actn3
Ndufs1
Ccnb1-ps
Atp7a
Abcd1
Ndufaf1
Mlxipl
Uqcr11
mt-Nd6
Chchd2-ps
Atpsckmt
Coq7
Uqcrc1
Atp5pb
Sdhaf2
Cox4i2
Ndufv1
Iscu
1700066M21Rik
Bdnf
Ndufs8
Dnajc15
Dnajc30
Dld
Ndufb2
Snca
mt-Nd4l
Ndufs4
mt-Atp6
Uqcrh
mt-Atp8
Afg1l
Uqcc3
Stoml2
Ndufa5
Ndufa12
mt-Co3
Fxn
Ndufs7
Tefm
Coa6
Ndufa8
Ndufb11
Ndufs6
Cyct
Ndufa1
Ccnb1
Uqcrb
Ndufa11
Cdk1
Cox8c
Ndufb6
Pink1
Ndufb1
Ndufa7
Slc25a33
Ndufb10
Cox5a
Cox5b
Cox7c
Ndufc2
Ndufa2
Ndufb3
Ndufab1
Ndufs5
Ndufa3
Tmem135
Ndufb9
Cox7b
Ndufc1
Cox7a1
Sdhc
Ndufv3
Ndufa13
Ndufs2
Chchd2
Sdha
Ndufb4
Sdhb
Nipsnap2
mt-Nd5
mt-Nd1
Cox6a1
Uqcr10
Ak4
mt-Co2
2iLIF
EPSC SOX2+
KOSR SOX2+
EPSC DP
KOSR DP
1.00
0.75
0.50
0.25
0.00
−0.25
−0.50
−0.75
−1.00
B
Glycolysis genes
Tpi1
Gapdh
Hk1
Nupr1
Eno1b
Eno1
Pkm
Pgk1
Myc
Ppp2ca
Eif6
Stat3
Hk2
Pgam1
Eno3
Galk1
Ucp2
Galt
Tigar
Git1
Foxk2
Prkaa2
Esrrb
Pfkl
Aldoc
Gpi1
Dhtkd1
Aldoart1
Zbtb7a
Bpgm
Mlx
Khk
Ogdh
Pfkp
Ep300
Prkaca
Pfkm
Jmjd8
Kat2b
Aldoa
Mpi
Mlst8
Col6a1
Aldoart2
Eno2
Hkdc1
Lipa
Ddit4
Sik2
Pfkfb1
Gck
Flcn
Rptor
Arl2
Pfkfb2
Foxk1
Prkaa1
Hdac4
Slc4a4
Ier3
Slc4a1
Eno4
Sirt6
Ncor1
Actn3
Tkfc
Adpgk
P2rx7
Pgam2
Mlxipl
Gapdhs
Igf1
Cbfa2t3
Mtor
Enpp1
Psen1
Hif1a
Gale
Ogt
Mtch2
Fkrp
Prkag2
Gpd1
Fbp1
App
Insr
Prxl2c
KOSR SOX2+
EPSC DP
KOSR DP
2iLIF
EPSC SOX2+
1.00
0.75
0.50
0.25
0.00
−0.25
−0.50
−0.75
−1.00
C
Lipid metabolism genes
Cpt1a
Gene expression [AU]
***
SOX2+
DP
Acaa2
Gene expression [AU]
***
SOX2+
DP
Slc25a20
Gene expression [AU]
***
SOX2+
DP
Abhd4
Gene expression [AU]
***
SOX2+
DP

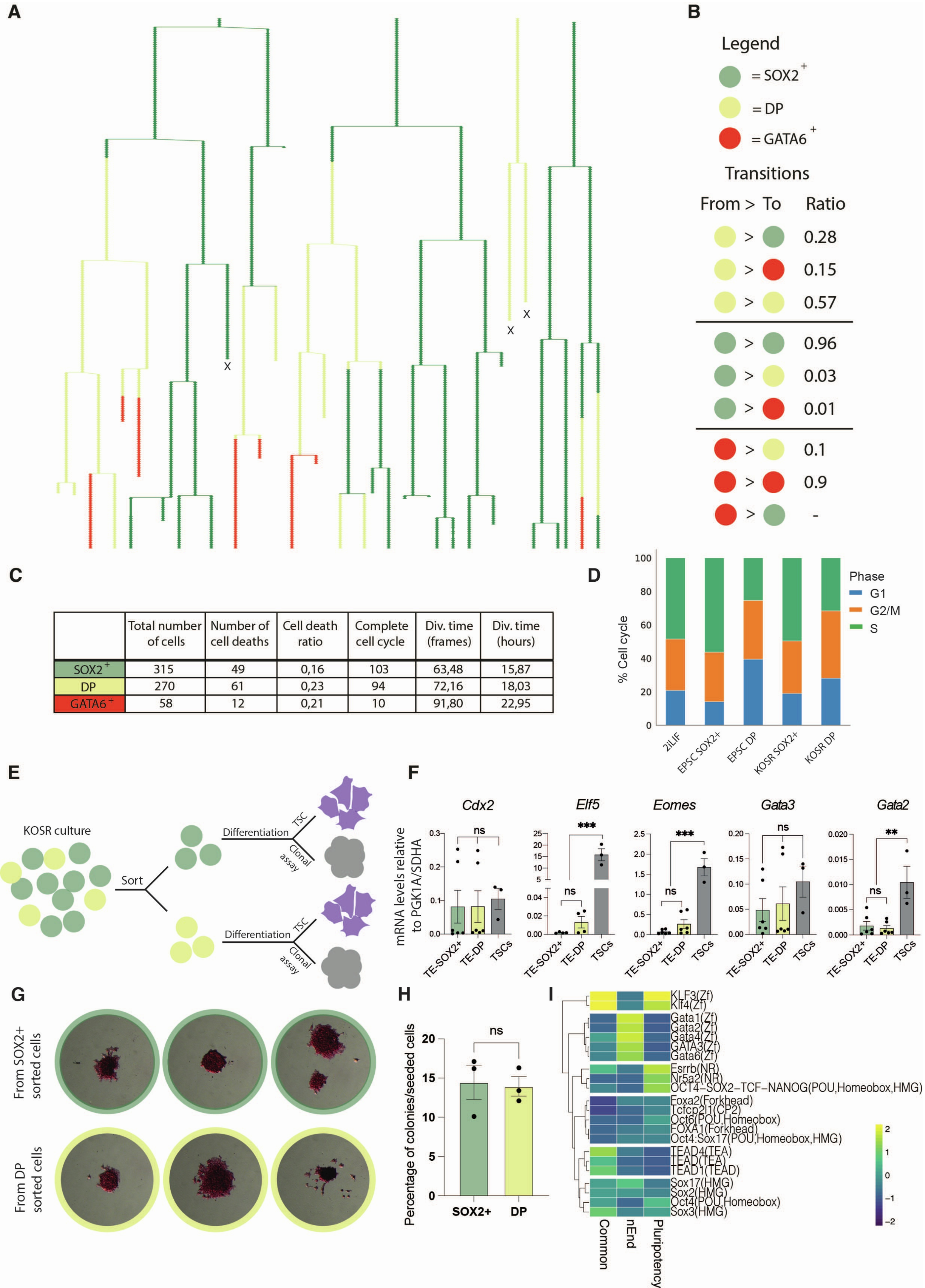

	Total number of cells	Number of cell deaths	Cell death ratio	Complete cell cycle	Div. time (frames)	Div. time (hours)
SOX2+	315	49	0,16	103	63,48	15,87
DP	270	61	0,23	94	72,16	18,03
GATA6+	58	12	0,21	10	91,80	22,95

Supplemental Figure Legends

Fig. S1. Generation of a post-translational double reporter for SOX2 and GATA6. A. Schematic drawing of the WT and transgenic sequences at the *Gata6* locus, including the Gata6 probe used for the Southern blot and the predicted band sizes when cut with enzymes NdeI and AflIII. **B.** Southern blot of the external probe (marked in A) for the Gata6-mCherry construct. 3 correctly targeted SGGC clones (B9, B12 & E1). **C.** Western blot for SOX2 and GAPDH showing two SOX2 bands (endogenous and GFP-tagged) in all the double reporter lines and in the parental line SG16 in 2iLIF conditions. **D.** Western blot for GATA6 and GAPDH showing 2 GATA6 bands (endogenous and mCherry-tagged) in all the double reporter lines in nEnd conditions. **E.** Bright field images of the correct karyotyping of the 3 SGGC clones. Scale bar = 15µm. Modal number for the 3 clones is 40 chromosomes: SGGC B9 at 77% (7/9 spreads) SGGC B12 at 75% (6/6 spreads) and SGGC E1 at 83% (5/6 spreads). **F.** Representative images of the SGGC lines in 2iLIF (naive ESCs) and at day 7 of PrE differentiation (RACL media). **G.** Representative flow cytometry plots of the SGGC cell line in 2iLIF and day 7 of PrE differentiation (RACL media).

Fig. S2. Cell populations identified with the SOX2 and GATA6 double reporter. A. PCA showing the first and second principal components. PC1 separates 2CLCs from the rest. **B.** Expression of 2C genes (*Zscan4c*, *Zscan4d)* projected on PCA plot shown in S2A. **C.** UMAP of single cell RNA-seq of sorted populations (top left) overlain with expression of selected markers (*Gata6, Zfp42* and *Sox2*). **D.** Heatmap showing normalized residuals post-$\chi 2$ test comparing differences in proportions of the sorted cells mapped to *in vivo* scRNA-seq of pre-implantation blastocysts (Nowotschin et. al 2019). Color scale represents the normalized deviation of observed from expected proportions. **E.** Violin plots depicting 4 selected genes from each relevant stage; E3.5 ICM, E3.5 Epi, E3.5 PrE and E4.5 Epi. The plot shows normalized and log-transformed counts. Wilcoxon statistical test used. ns = non-significant * = p-value < 0.05 ** = p-value <0.01 and *** = p-value <0.001. **F-G.** GO term enrichment for KOSR DP cells compared with EPSCM DP cells. **H-I.** GO term enrichment of KOSR DP compared with KOSR $SOX2^+$.

Fig. S3. Metabolic signatures present in DP SOX2 and GATA6 expressing ESCs. A. Heatmap showing oxidative phosphorylation genes expression in the different media conditions. **B**. Heatmap showing glycolysis genes. **C.** Violin plots with different examples of lipid metabolism genes distribution in DP or $SOX2^+$ cells in KOSR media. Mann-Whitney U statistical test. * = p-value 0.01, *** = p-value 0.001

Fig S4. Interconversion and differentiation of SOX2 and GATA6 expressing ESCs. A. Representative dendrogram of 4 initial tracked cells with its descendants (from Video S1). A total of 63 individual tracks of 72h were tracked. The x marks cell death. **B.** Ratios of transitions from one cell state to another. **C.** Table with quantification of the timelapse data. **D.** Distribution of the cell cycle phases in different sorted populations based on scRNA-seq data. For each cell, its cell cycle phase was estimated using CellCycleScoring function from Seurat. The barplot depicts cell cycle phase proportion for different cell stages. **E.** Schematic of TSC differentiation and clonal assay after sorting KOSR cells by FACS. **F.** Relative mRNA levels of TSC markers of the sorted $SOX2^+$ (green bar) or DP (yellow bar) sorted populations after 6 days of TSC differentiation. 2 individual clones were used. Grey bars show control values from Trophoblast stem cells derived from post-implantation embryos. Columns show mean ±

standard error of the mean (SEM). Statistics show unpaired t-tests. ns = non-significant * = p-value < 0.05 ** = p-value <0.01 and *** = p-value <0.001. **G.** Representative images of AP-stained colonies after 1 week of clonal growth from SOX2+ sorted cells (green frame) or DP sorted cells (yellow frame). **H.** Quantification of the AP staining from the clonal assay. **I.** Heatmap of selected motifs across enhancer subsets defined in Figure 3B, with a cutoff of p-value < 0.0005. Scale shows -log10(p-value).

Tables:

Table 1 SOX2 and GATA6 binding. Table shows a list of the closest epiblast genes to the cobound peaks for SOX2 and GATA6 in DP cells. It also shows how many SOX2 and GATA6 motifs we find in these regions. Genes colored in yellow are differentially upregulated in DP over SOX2$^+$ cells in the scRNA-seq. The ones colored in green are differentially upregulated in SOX2$^+$ over DP cells.

Table 2 Characterization of DP SOX2 and GATA6 cobound regions. Table shows a list of the closest PrE genes to the cobound peaks for SOX2 and GATA6 in DP cells. It also shows how many SOX2 and GATA6 motifs we find in these regions. Genes colored in yellow are differentially upregulated in DP over SOX2$^+$ cells in the scRNA-seq. The ones colored in green are differentially upregulated in SOX2$^+$ over DP cells.

Table 3 Comparison of this study to Thompson *et al. 2022*. Table compares SOX2 and GATA6 binding peaks from our study with the SOX2 and GATA6 peaks of the GATA6 overexpression time course from Thompson *et al.* 2022.

Supplemental experimental procedures

Generation of SGGC ESC lines

SOX2:GFP ESCs were plated onto a gelatinised 6-well plates in serum/LIF and lipofected with the linearized Gata6:mCherry-Hygromycin plasmid and the CRISPR plasmid using Lipofectamine2000 (ThermoFisher) following the manufacturer's instructions. After 16 h incubation, the cells were transferred onto 10cm dishes with fresh medium supplemented with Hygromycin to select for colonies successfully integrated. SOX2:GFP ESCs (Anderson et al., 2017) (specifically clone SG16) was used to further target with a Gata6:mCherry construct using CRISPR-Cas technology. The construct contains mCherry tagged immediately after exon 7 of the Gata6 locus, just before the STOP codon and the construct has 3000bp homology arms. We obtained 3 clones (B9, B12 and E1) that were successfully integrated. Clone B9 was generated using CRISPR guide 1 (GCTCTGGCCCTGGCC), which cuts at the last 20nt of the coding sequence of Gata6, and clones B12 and E1 were generated using CRISPR guide 2 (GCACAGAAATCACGCATCGA), which cuts 150 bp after the STOP codon (See Fig. 1A).

SGGC cells were verified by performing immunostaining for SOX2, GATA6, mCherry and GFP, western blot, locus sequencing to screen for unwanted mutations generated by CRISPR, karyotyping and Southern blot. Resistance cassettes can be easily removed using Cre-mediated recombination, however we decided to use the original clones with the resistance cassettes included. All 3 clones give the same reproducible results. All 3 clones were used for the MARSeq scRNA-seq. Clones B9 and B12 presented the highest amount of double positive cells, so these 2 clones were used in all the CUT&RUN, differentiations and morula aggregation experiments.

Karyotyping

SGGC ESCs were expanded until 50-60% confluency, after which they were incubated for 1h in medium containing 10 µg/ml Colcemid (Sigma Aldrich). The medium was collected for separate disposal, and without a PBS washing step, 2 ml of trypsin were added. When the first cells started to detach, the trypsin was inactivated by adding medium. Pelleted cells were resuspended in 2.5 ml hypotonic solution (0.56 % (w/v) KCl and incubated at RT for exactly 6 min. 1 ml of fixative (75 % (v/v) methanol, 25 % (v/v) acetic acid) was added, followed by 1 wash in 1 ml of fixative and 30 min incubation at 4°C. After 2 washes in fixative, cells were carefully resuspended in 200µl fixative and spread onto pre-cleaned poly-L-lysine coated glass slides. Chromosomes were stained for 30 min in filtered 10% Giemsa pH 7.2 solution and imaged using a 63x Oil objective on an inverted Olympus microscope.

Southern blot

Southern Blotting was used to test SGGC clones for correct gene targeting as previously described (Southern, 2006). Gata6:mCherry construct integration was confirmed using both an internal (mCherry) and an external probe. DNA for the external probe was cut using AflIII and NdeI. The DNA for mCherry internal probe was cut using HindIII (Fig. S1A-B). Hybridization of the probe happened at 60°C O/N. The blots were left in the exposure cassette for 48h and developed using a high-resolution Typhoon scanner.

Flow cytometry and FACS

Cells were washed in PBS and brought to single cell suspension using Accutase. Cells were resuspended in 10% FCS/PBS with DAPI (1:10,000) to exclude dead cells. Cells were analyzed using a LSR Fortessa flow cytometer (BD Biosciences) and the FACSDiva (BD Biosciences) software. Cells were sorted by SOX2$^+$ or DP by FACS using a BD FACS Aria III (BD Biosciences) with a 100µm nozzle and 20psi sheath pressure. The gate between positive and negative populations were set based on the negative population of control ESCs (both E14 and parental line SG16). A forward scatter (FSC) and side scatter (SSC) was used to define a homogeneous population, FSC-H/FSC-W gates were used to exclude doublets, and dead cells were excluded based on DAPI staining. Plots were generated using FCS Express 6.0 (DeNovo Software).

Alkaline Phosphatase Staining

ESCs were plated at clonal density and cultured for 7 days. Alkaline phosphatase staining was carried out as per manufacturer's instructions. Colonies were imaged using a Leica M165 C microscope.

ESC and embryo immunostaining

ESCs were cultured in 8-wells slides (Ibidi) and immunostaining was carried out as previously described (Canham et al., 2010). Primary antibodies are listed in the resource table. Secondary antibodies used are from the Alexa Fluor series (Molecular Probes, ThermoFisher), all 1:2000. Blastocysts were stained by anti-CDX2 (BioGenex MU392A-UC) used at 1:200. Both for blastocyst and E6.5 embryos anti-GATA6 (Cell Signalling Technologies, 5851) was used at 1:200. Both mESCs and embryos were imaged using a confocal Leica SP8.

Quantitative PCR with reverse transcription (RT-qPCR)

Total RNA was collected using the RNeasy Mini Kit (Qiagen). 1µg of total RNA was used for first strand synthesis using SuperScript III reverse transcriptase according to the manufacturer's

instructions. cDNA corresponding to 10ng total RNA was used for RT–qPCR analysis using the Roche LC480 and target amplification was detected with the Universal Probe Library system.

scRNA-seq

Cells were sorted directly into 384-well plates containing lysis buffer which includes the first RT primer and RNase inhibitor, immediately frozen and later processed by the MARS-seq2 protocol (Keren-Shaul et al., 2019). scRNA-seq libraries were sequenced using an Illumina NextSeq500 at a median sequencing depth of 225,000 reads per single cell. Pre-processing was done using nfcore/marsseq pipeline (Proks et al., 2023) with following command: nextflow run nf-core/marsseq -r 1.0.3 -profile ku_sund_dangpu –with-tower --genome mm10 --velocity --input SCR_20221006/raw/samplesheet.csv --outdir /scratch/ALBA_SB2/ .

Time-lapse imaging and cell tracking

SGGCH2B ESC lines were cultured in KOSR media, on 8-well slides (ibidi) and imaged every 15 minutes across 72h. mCherry, GFP and CY5 fluorescent light channels were recorded in 5% CO_2, 20% O_2 at 37°C under a Deltavision Widefield Screening microscope. ESCs were seeded at 5000 cells/cm^2 24h before the beginning of the time lapse in KOSR. We performed manual cell tracking using Imaris v9.5 (Bitplane). Nuclei were segmented using the H2B marker tagged with far red. We measured the SOX2-GFP and GATA6-mCherry fluorescence intensities of a circular area of 50 µm diameter inside the segmented nuclei. For each area measured, we took the median fluorescence intensity as the measure for that given data point. Intensity measurements were linked to its time point and lineage, allowing us to infer the division time for each cell that was tracked, as well as the expression level of both SOX2 and GATA6 in each time point. Only cells with completed cell cycle information were used for calculating the transition analysis. A total of 63 individual tracks of 72h have been tracked.

CUT&RUN

KOSR, nEnd, 2iLIF or NACL cells were grown in their respective media for at least 4 passages. KOSR and nEnd cells were sorted by FACS using a BD FACS Aria III. From KOSR we sorted SOX2$^+$ and DP cells. From nEnd we sorted GATA6$^+$ cells. A minimum of 100,000 cells were sorted to proceed with the CUT&RUN protocol. CUT&RUN was performed using an in house purified MNase and following the published protocol (Janssens and Henikoff, 2019). Library preparation was performed following this published protocol (Liu, 2019). The quality of the CUT&RUN libraries was assessed using an Agilent Fragment Analyzer and quantified using Qubit. Sequencing was performed on an Illumina NextSeq 550, up to 48 uniquely barcoded samples were pooled on a High Output flow cell. Samples are sequenced using paired end sequencing. Antibodies used: Sox2-Active Motif 39843, Gata6-Cell Signaling 5851, H3K27ac Abcam-ab4729, H3K4me1 Abcam-ab8895. All used at 1:100 concentration.

Downstream analysis of CUT&RUN

Reads were trimmed to 21bp with Cutadapt (Martin, 2011), mapped to the GRCm38/mm10 mouse reference genome with Bowtie 2 (Langmead and Salzberg, 2012), with options -X 1000 –no- mixed –no-discordant. Only uniquely mapped reads with MAPKQ>10 were kept using SAMtools v1.9 (Li et al., 2009). Bam files were sorted, deduplicated with Picard v2.9.1 and indexed. Bam files for TFs were subset to contain a maximum fragment length of 150 bp, whereas for histone modifications, all reads were considered. Bedgraphs were generated using bedtools v2.27.1. (Ar and Im, 2010). Bigwig files were generated with bamCoverage

v3.2.0 from DeepTools (Ramírez et al., 2016) and the RPGC option (-bs=1 –normalizeUsing RPKM). Peaks were called using SEACR (Meers et al., 2019), parameters; 'relaxed' for transcription factors and 'Stringent' for histone marks. Peaks were called against a negative IgG control, generated in each experiment for each condition. GREAT was used to annotate peaks to nearest genes and perform GO enrichment (McLean et al., 2010). Motif analysis was performed on select genomic regions using HOMER findMotifsGenome.pl with options -size 75 -mask (Heinz et al., 2010). Genome browser tracks were visualized using Gviz/Fluff (Hahne and Ivanek, 2016). Global TF occupancy across enhancer subsets was plotted by first calculating the coverage at each enhancer summit +/- 1 kB using Deeptools computeMatrix (Ramírez et al., 20) and visualized in R using custom scaling that equalized TF signal across enhancer subsets to assesses the relative TF distribution on enhancers within each condition.

Chimera assays

For chimera assays, H2B:miRFP670 tagged clones SGGCH2B B9.A and B12.B were used. Cells were sorted for $SOX2^+$ or DP as previously described. Using an aggregation needle (Type DN-09, BLS Ltd., Hungary), 10 to 12 wells were made in 35mm tissue culture Petri dishes. The wells were covered with microdrops of KOSM and mineral oil. The plate was left in the incubator for 1h at 37°C and 5% CO2 to buffer the medium to the appropriate pH. Live morulae (E2.5) were flushed from the uterus of superovulated C57BL/6NRj female mice by the transgenics platform facility and cultured in KSOM.

DP or $SOX2^+$ sorted cells were placed at the bottom of the wells and 1 morula was added on top.

Embryos were either cultured for 3 days *in vitro* to the equivalent of E4.5 *in vivo* or transferred to pseudopregnant CD1 females (RjOrl:SWISS) (12 weeks old) for further development, and embryos were harvested at E6.5. Animal work was carried in accordance with European legislation. All work was authorized by and carried out under Project License 2018-15-0201-01520 and 2023-15-0201-01513 issued by the Danish Regulatory Authority.

qPCR primers

Gata6F	GGTCTCTACAGCAAGATGAATGG
Gata6R	TGGCACAGGACAGTCCAAG
Gata4 F	TTCGCTGTTTCTCCCTCAAG
Gata4 R	CAATGTTAACGGGTTGTGGA
Gata3 F	TTATCAAGCCCAAGCGAAG
Gata3 R	TGGTGGTGGTCTGACAGTTC
Dab2 F	TCTCAGCCTGCATCTTCTGA
Dab2 R	TTTGCTCATCTGGATAGTCATCAT
Nanog F	cctccagcagatgcaagaa
Nannog R	gcttgcacttcatcctttgg
Oct4 F	GTTGGAGAAGGTGGAACCAA
Oct4 R	CTCCTTCTGCAGGGCTTTC
Oct6 F	catttttcgtttcgttttaccc
Oct6 R	gagcgcagaccctctctg
Otx2 F	ggcctcactttgttctgacc
Otx2 R	aaatcaacttgccagaatcca
Pdgfra F	AAGACCTGGGCAAGAGGAAC
Pdgfra R	GAACCTGTCTCGATGGCACT
Sox7 F	gcggagctcagcaagatg
Sox7 R	gggtctcttctgggacagtg
Sox17 F	CACAACGCAGAGCTAAGCAA
Sox17 R	CGCTTCTCTGCCAAGGTC
Cdx2 F	CACCATCAGGAGGAAAAGTGA
Cdx2 R	CTGCGGTTCTGAAACCAAAT
Elf5 F	GCAGCTCTGCAGCATGAC
Elf5 R	TTCAGCATCATTGAAAAAGGAG
Eomes F	AAGCTCAAGAAAGGAAACATGC
Eomes R	ACCGGCACCAAACTGAGA
Gata2 F	CTAGGAGTTTGGCTGAAAGGG
Gata2 R	CTCTGCCCAGATTGCCACTG

Antibodies

ANTIBODY	BRAND	# ID	CONCENTRATION	USE
SOX2	Active motif	39843	1:1000	CUT&RUN
GATA6	Cell Signaling	5851	1:1000	CUT&RUN, IF
H3K27ac	Abcam	ab4729	1:1000	CUT&RUN
H3K4me1	Abcam	ab8895	1:1000	CUT&RUN
SOX2	Santa Cruz	sc17320	1:200	IF
CDX2	BioGenex	MU-392A-UC	1:200	IF
